# Feedback circuits are numerous in embryonic gene regulatory networks and offer a stabilizing influence on evolution of those networks

**DOI:** 10.1186/s13227-023-00214-y

**Published:** 2023-06-16

**Authors:** Abdull Jesus Massri, Brennan McDonald, Gregory A. Wray, David R. McClay

**Affiliations:** Department of Biology, Duke University, Box 90338, Durham, NC 27708 USA

**Keywords:** Gene regulatory networks, Feedback circuits, Evolutionary mechanism, Embryonic specification, Sea urchin development

## Abstract

The developmental gene regulatory networks (dGRNs) of two sea urchin species, *Lytechinus variegatus (Lv)* and *Strongylocentrotus purpuratus (Sp),* have remained remarkably similar despite about 50 million years since a common ancestor. Hundreds of parallel experimental perturbations of transcription factors with similar outcomes support this conclusion. A recent scRNA-seq analysis suggested that the earliest expression of several genes within the dGRNs differs between *Lv* and *Sp*. Here, we present a careful reanalysis of the dGRNs in these two species, paying close attention to timing of first expression. We find that initial expression of genes critical for cell fate specification occurs during several compressed time periods in both species. Previously unrecognized feedback circuits are inferred from the temporally corrected dGRNs. Although many of these feedbacks differ in location within the respective GRNs, the overall number is similar between species. We identify several prominent differences in timing of first expression for key developmental regulatory genes; comparison with a third species indicates that these heterochronies likely originated in an unbiased manner with respect to embryonic cell lineage and evolutionary branch. Together, these results suggest that interactions can evolve even within highly conserved dGRNs and that feedback circuits may buffer the effects of heterochronies in the expression of key regulatory genes.

**Supplementary Information:**

The online version contains supplementary material available at 10.1186/s13227-023-00214-y.

## Background

Developmental gene regulatory networks (dGRNs) are models of transcriptional and signaling circuits that pattern embryos, direct specification and differentiation of cells, and guide morphogenesis. Mutations in the genes and regulatory elements that encode these critical developmental processes are major drivers of phenotypic diversification because these segments of the genome play direct roles in producing morphology and a wide variety of other organismal traits [1–3]. Yet dGRNs also represent highly resilient systems, compensating for diverse environmental perturbations and mutations to produce consistent developmental outcomes. This resiliency poses a conundrum: how is it possible for critical developmental mechanisms to evolve and thereby alter organismal traits if the organization of regulatory interactions has been selected to buffer perturbations so efficiently?

**Fig. 1:**
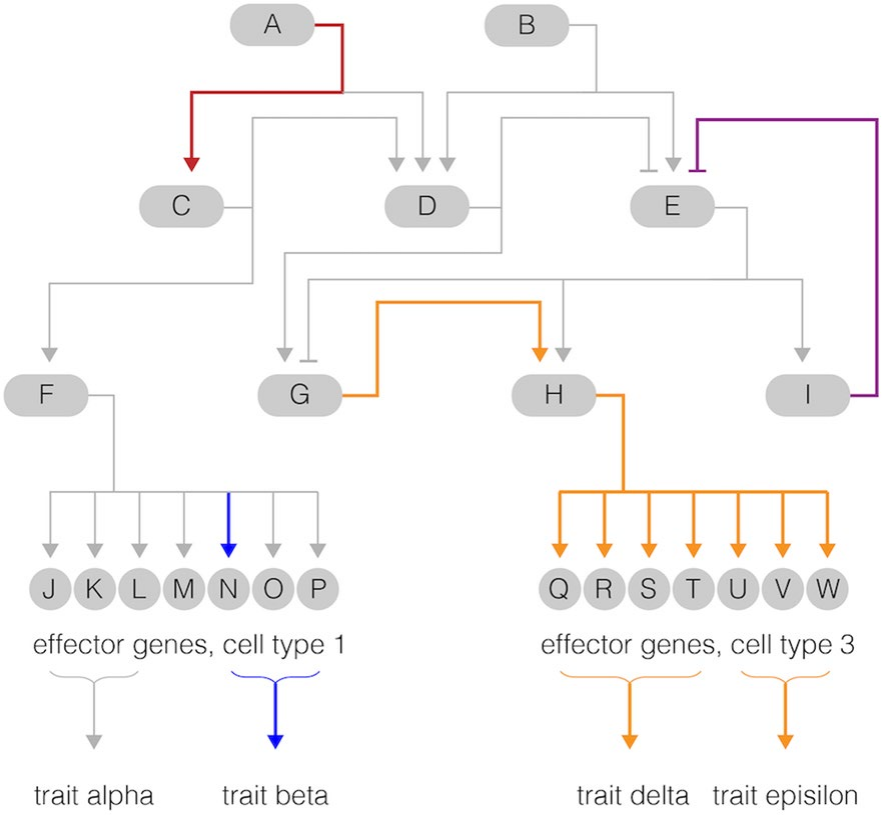
Evolutionary changes in a hypothetical Gene Regulatory Network (GRN). The consequences of altering an interaction within a GRN can differ enormously, depending on local context. Here, ovals represent genes and arrows represent the activity of that gene product as transcriptional activation (—>) or inhibition ( -|) of another gene. Altering a connection near the beginning of the GRN (red arrow) is more likely to have widespread effects than a connection that involves a single gene at the periphery of the network (blue arrow). Genes encoding regulators near the periphery are in a position to alter functionally related sets of genes in a coordinated manner (gold arrows) that might change a single trait without altering others. Interactions that provide feedback inhibition (purple arrow) or feedback activation can stabilize expression if changes evolve elsewhere in the GRN

Comparing dGRNs among species provides some clues. An important general finding is that mutations commonly influence the expression and function of effector genes at the periphery or "end" of the dGRN [3, 4] (Fig. 1**,** blue arrow). Effector genes encode a wide variety of enzymes, transporters, structural proteins, and other proteins that execute morphogenesis, physiology, and the specialized functions of differentiated cells. Numerous case studies demonstrate that changing the regulation of a single gene encoding an effector protein can alter a trait in an adaptive way [5–9]. This fits intuition, since mutations at the periphery of a dGRN will likely impact just one or a few traits because of their topological position within the network. The resulting low degree of pleiotropy should allow natural selection to operate efficiently.

Yet it is clear from comparing more distantly related species that it is also possible for changes to evolve deep within dGRNs, in some cases involving even the earliest and most fundamental patterning events in embryogenesis (Fig. 1, red arrow). Mutations that alter dGRNs in such profound ways are likely to have a larger and broader phenotypic impact than those occurring at the periphery. Such mutations are much less likely to be net neutral or beneficial, because so many later developmental events depend on the successful execution of early ones. Often genes at this level of a regulatory circuit are referred to as “master regulators”. How evolutionary changes arise deep within dGRNs is thus not at all clear. One possibility is that exceedingly rare mutations are able to alter a fundamental interaction early in a dGRN without wreaking havoc on later developmental processes, while even rarer mutations might in addition alter an organismal trait in a beneficial way. In this way, a dGRN could occasionally change neutrally or even adaptively. Identifying such mutations would be a challenge, however, given their inherent rarity and the likelihood of becoming obscured by more frequent changes at the periphery of the dGRN.

Another possibility is that the structure of the dGRN could facilitate evolutionary change. Feedback circuits are particularly interesting candidates because they can stabilize gene expression from genetic and environmental perturbations [8, 10–14]. For instance, a feedback regulatory circuit could tune the expression of a gene up or down in response to over- or under-production of a downstream gene [15] (Fig. 1, purple arrow). Perhaps the most straightforward way feedbacks could facilitate evolutionary changes deep within a dGRN is by limiting pleiotropy: a mutation might change the expression of a key regulatory molecule but the expression of most downstream genes might be largely buffered, due in part to feedback circuits, thereby limiting the impact of the mutation to just one or a few organismal traits. Other types of interactions within dGRNs could also potentially provide buffering, including mutual reinforcement, mutual exclusion, and parallel inputs [15], but here we focus primarily on feedback circuits.

One way to gain a better understanding of how dGRNs evolve is to compare well-defined networks in multiple species. The sea urchins *Strongylocentrotus purpuratus*, *Lytechinus variegatus* and *Paracentrotus lividus* (henceforth, *Sp Lv,* and *Pl*) provide a valuable opportunity. These species diverged ~ 40–50 million years ago [16] and their dGRNs have been studied extensively. In 2006, the *Sp* genome was published and accompanying papers annotated 639 transcription factors [17–20] and 182 genes involved in commonly used embryonic signal transduction pathways [21–23]. Of those 821 genes, 81 are included in published dGRNs of endomesoderm and ectoderm in the three species most commonly studied [24–26, 44–51]. These were chosen for study based on expression early in development, and expression in spatially restricted and/or temporally specific patterns. Many of the remaining 740 genes are expressed ubiquitously, or in some cases, not expressed during embryonic development. Assembly of dGRNs was based on perturbation of a transcription factor or signal with consequential changes observed in other genes or signals among the remaining 80 genes. Where comparisons have been conducted between the three species, both the territorial expression, and perturbation outcomes have been reported to be similar. This suggests that the dGRNs of these three species are highly conserved despite nearly 50 million years separation from common ancestors.

Nonetheless, conservation in dGRNs is occasionally broken. A prominent example can be found in the sea urchin genus *Heliocidaris* [27]. The dGRN of *Heliocidaris tuberculata*, while not studied in the same detail as that of *Sp* and *Lv*, expresses network genes examined in spatially and temporally similar patterns as *Sp, Lv,* and *Pl* [28–33]. In contrast, its close relative *H. erythrogramma* contains several prominent changes in its dGRN, not just at the periphery but also in some of the earliest interactions [28, 34, 35]. These changes likely represent adaptations to a highly modified life history that evolved in < 10 million years [27].

What allows for changes within a dGRN, particularly when this follows a much longer period of prior conservation? We hypothesize that feedback provides part of the answer. By limiting the impact of mutations to specific parts of the overall network, feedback regulation may provide a level of GRN stability that allows changes to accumulate in dGRNs over evolutionary time. Here we present a detailed comparison of the dGRNs of *Sp* and *Lv* based on published datasets and with a dataset obtained from a dense scRNA-seq analysis [36]. That dataset was used to extract time of first expression of the 81 genes in multiple cell lineages in *Lv*. Examination and comparison of these datasets identify feedback inputs and evolutionary changes in the time of first embryonic gene expression when compared to datasets from *Sp* and to a more limited extent, to *Pl*. We used the dataset from Materna, et al., [37], using Nanostring to approximate first expression in *Sp,* and data from Gildor and Ben-Tabou de-Leon [38], that approximates time of first expression of 25 network genes in the *Pl* embryo. This comparison allowed us to predict the likely source of evolutionary change where those were detected. The comparison reveals a number of heterochronies in networks that otherwise are extremely similar. When timing of first expression is carefully considered in both *Sp* and *Lv* dGRNs*,* a number of feedback circuits are present that were not previously recognized. These, based on known properties of feedback circuits, we conclude, are likely to provide stability to the dGRNs, even after introduction of a number of timing changes over time as revealed by the evolutionary introduction of many heterochronies.

## Results

A dense temporal scRNA-seq analysis of the first 24 h of *Lv* development was computationally strengthened using Waddington-OT [39]. This tool takes all genes expressed, and their expression level, by a cell at time point 1 and asks, at timepoint 2, which cell(s) most closely match that cell. The match continues through all the timepoints resulting in a lineage relationship that reflects the strongest lineage trajectory among all cells in the dataset. A control involves drop-out measurements in which cells at one timepoint are dropped out to determining the probability that the same trajectory would result. This control is important because if timepoints are too far apart, the trajectory probabilities become far less significant. The dataset parameters were optimized in this way, and the probability of reaching lineage endpoints reached 100% by 16–18 h of the 24 h temporal sequence.

This dataset is publicly available (see Methods) and was used to approximate time of first expression of the 81 genes in the published dGRNs (Additional file 1: Fig. S1, Additional file 5: Table S1). The accuracy of the first expression times was independently assessed for 25% of the 81 genes in the *Lv* dGRN dataset using qPCR (Additional file 6: Table S2). This separate approach was in strong agreement with the scRNA-seq-obtained data providing support for the notion that the scRNA-seq dataset can be mined for approximations of time of first expression of a gene. As these data were obtained, we realized that some of the time points differed relative to first expression in *Sp* as recorded by Nanostring [37]. This was surprising to us because after two decades of research on dGRNs in both *Sp* and *Lv,* there had been a consensus that the two dGRNs were extremely similar. They both incorporated the same signals, used the same transcription factors, and perturbations of each of the genes tested resulted in very similar outcomes. This prompted us to carefully evaluate the relationship between the two dGRNs in those two species separated by about 50 million years from a common ancestor. The data reveal a number of heterochronies in timing of initial zygotic gene expression between *Sp* and *Lv*. Some of these are large relative to the duration of embryonic development. We also re-examined the topology of the dGRNs of the two species and observe numerous feedback circuits in both species, many of which have not been previously recognized. Gene expression data from a third species, *Pl *[38]*,* were used to infer the polarity of heterochronies. The analysis focuses on the four major cell lineages that are specified prior to gastrulation: ectoderm, endoderm, endomesoderm, and skeletogenic mesoderm.

### Updated dGRN models incorporate time of first zygotic expression

**Fig. 2:**
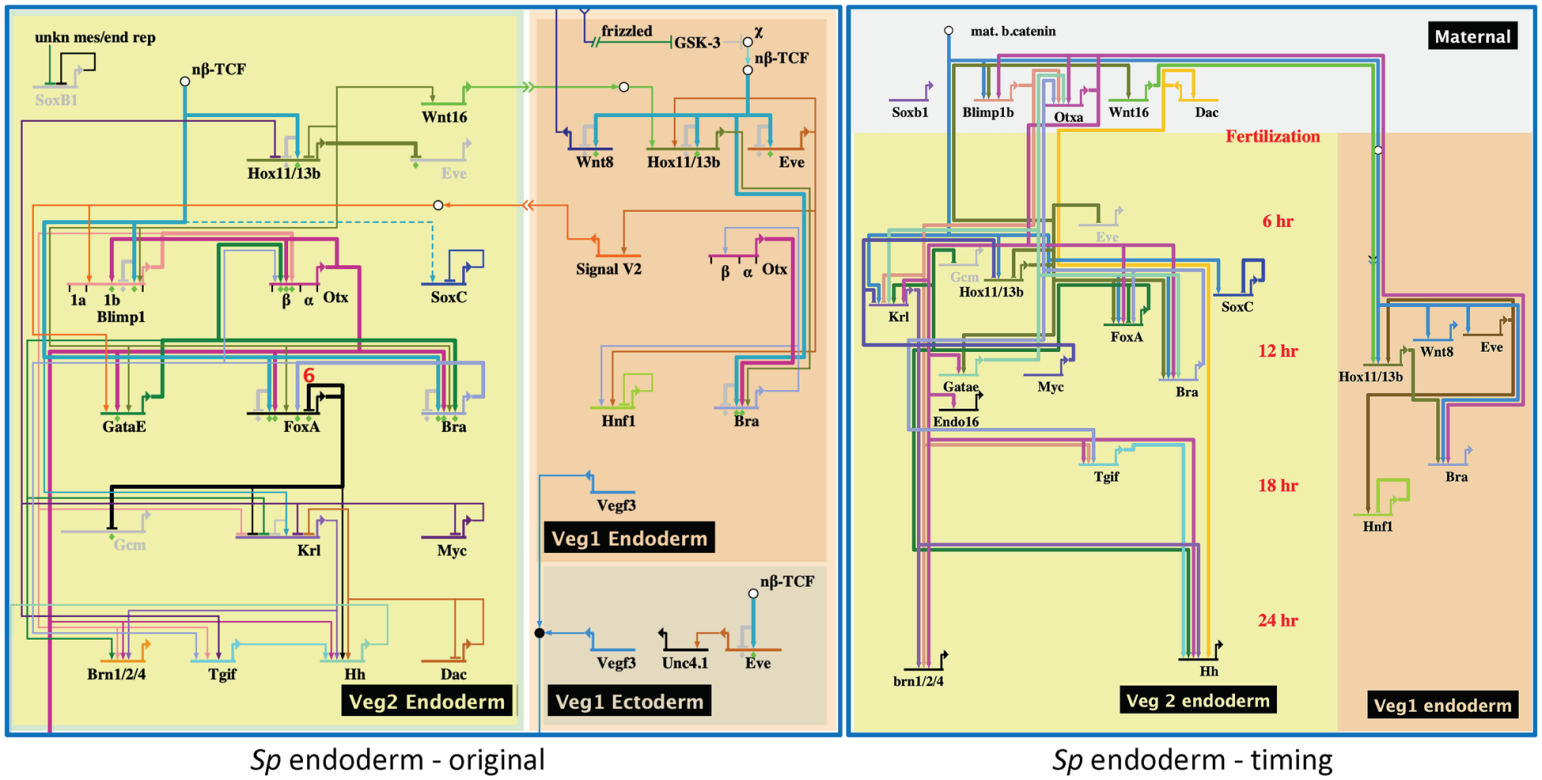
Classic and updated dGRNs of the endoderm of *Sp.* On the left is the classic model of the endoderm dGRN using the graphic program BioTapestry [61] (modified after [26]). On the right is an updated version of that GRN with each gene placed along the time line from top to bottom to reflect its time of first expression. Time of first expression is from [37]. Note, when timing is considered, the early specification of endoderm is largely compressed into a 7-h period of the first 24 h of development

The data from the nanostring treatment of *Sp* gene expression over time [37] were used to extract time of first expression of genes in that species. These times were compared with the published dGRN models. Figure 2 (left) shows the most recently published *Sp* endoderm dGRN model [40]. The graphic of that endoderm model focused on node relationships with less attention toward the precise timing of expression of the contributing transcription factors. We decided to re-draw the *Sp* dGRN with inclusion of timing of first expression in the dGRN model. We did this with no change in the network connections as they were already worked out in detail [40–45] and continue to serve as a reference for comparison with other echinoderm species [38, 46–53]. The updated model (Fig. 2, right) shows the same *Sp* endoderm dGRN model, updated to reflect timing of first expression (hpf shown in red). Note the compression of most interactions to an interval between approximately 6 and 15 hpf. This compression is also evident in updated dGRN models for the skeletogenic mesoderm (Additional file 2: Fig. S2A), endomesoderm (Additional file 2: Fig. S2B), and ectoderm (Additional file 2: Fig. S2C) of *Sp*. The temporal topology reveals that specification of each of the early lineages occurs over a compressed time period following a period dominated by expression of maternal transcripts. After the early zygotic pulse of specification, fewer additions to the network occur as morphogenesis approaches.

For the dGRN of *Lv*, we turned to our recent scRNA-seq analysis [36] and generated a dataset recording time of first zygotic expression (Additional file 5: Table S1, Additional file 1: Fig. S1). That dataset reflects two hours—the last hour during which expression of the gene in question occurs in few, if any, cells of a lineage, and the first hour of increase. Additional file 1: Fig. S1 shows graphs of expression the 81 genes over time. At each time point along the X axis, the number of cells expressing that gene is shown as a dot and the level of expression in that cell is shown on the Y axis. First expression time points were assigned to all 81 genes using these plots. The sensitivity of scRNA-seq may be lower than that of Nanostring, so we carried out a temporal analysis of 19 genes in the dGRN using qPCR (Additional file 6: Table S2). For 18 of the 19 genes assayed, the timing of first expression by qPCR, was within 1 h of the time first detected by scRNA-seq. The single exception was separated by 2 h. We conclude that scRNA-seq has sufficient sensitivity to provide an approximation of time of initial zygotic expression for genes in each lineage.

Additional file 7: Table S3 presents time of first expression for the 81 dGRN genes in *Sp* and *Lv*. Because the two species develop at different temperatures, we normalized developmental time of *Lv* to that of *Sp* (see Methods).

### Heterochronies are observed when time of first expression of *Sp* and *Lv* genes are compared

**Fig. 3:**
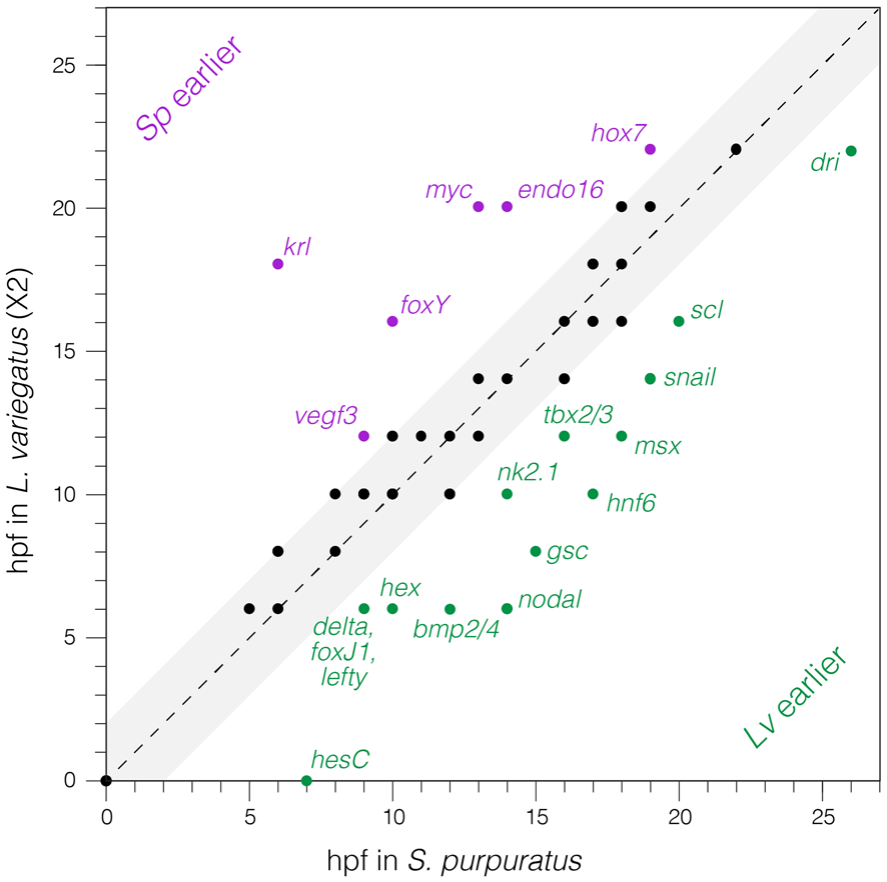
Time of first transcription of 81 genes in *Sp* vs *Lv.* The time of transcriptional activation of *Sp* is recorded along the X axis and time of gene activation of *Lv* along the Y axis. (The *Lv* times are doubled to normalize developmental progression based on temperature differences in culture.) The black circles are genes that are expressed at the same time (within ± 2 h) in both species, given that allowance for temperature normalization. The circles above or below the gray area on either side of the diagonal line represent genes that are heterochronically expressed between the two species. Those circles above the line are genes that are activated earlier in *Sp*, relative to time of activation in *Lv.* Circles below the line represent genes that are activated earlier in *Lv* relative to *Sp.* The circle at 0,0 represents 20 genes that are expressed maternally in both species. The gray box represents 2 h above and below the diagonal line. Genes within that box are considered to be expressed at the same developmental times in both species allowing for natural variation and/or sensitivity of detection to account for deviations from the diagonal line

To account for possible differences in the sensitivity of the assays used to measure expression, and for natural variation in developmental timing, we scored a gene as heterochronic only if the recorded time of first expression differed by more than ± 2 h in the comparison. Twenty-one of the 81 genes had temporal shifts of first expression differing by more than two hours (Additional file 7: Table S3). Of those, five genes are expressed in more than one lineage of the GRNs. The time of first expression could only record the lineage in which the first expression occurred (since the Materna et al., database was for whole embryos), and for each of the five, in situ analysis shows the same lineage in both species expresses the gene first. For example, *dri* is first expressed in the micromeres (skeletogenic lineage) and is expressed later in the endoderm. Whether a heterochrony in expression of *dri* exists in the endoderm is unknown given that the Materna database does not separate lineages. To illustrate the first expression comparison, Fig. 3 plots the relative time of first expression of the 81 GRN genes to indicate the variation in times. *Sp* gene expression times are recorded along the x-axis and *Lv* times along the Y axis. Each gene is represented by a circle; circles that lie along the black diagonal line represent genes that are initially expressed at the same normalized time in the same territory. Black circles that lie within the light gray box differ by less than ± 2 h between species, and represent genes that are not considered to be heterochronic based on a conservative estimate of experimental variation and detection limits. Circles above and below the gray box represent genes that are heterochronic by our criteria: those above the box are expressed earlier in *Sp* relative to *Lv*, while those below the box are expressed earlier in *Lv* relative to *Sp*.

Of the genes in the comparison, 20 are maternally expressed in both species. These are indicated by the single black circle at 0 hpf (Fig. 3). The remaining genes were first expressed zygotically in both species (Additional file 7: Table S3). Of these, 20 differ in time first expression by more than 2 h, with 6 expressed significantly earlier in *Sp,* and 14 significantly earlier in *Lv.* Altogether, 24.7% of the genes measured were expressed heterochronically relative to the other species.

Several further observations are noteworthy. First, the expression of 10 genes showed timing differences of greater than 4 h between species and 1 differed by 12 h of normalized expression. Given that most first times of zygotic expression within the dGRN occur prior to 20 hpf, heterochronies of ≥ 4 h represent proportionally large shifts in developmental timing. Some of these timing shifts are so large that a gene expressed upstream of a gene it controls in one species, is expressed *after* the downstream gene (see below). This necessitates a change in the topology of the dGRN models as described below. Second, the heterochronies are not concentrated in a limited portion of the dGRN; instead, they affect genes in each of the embryonic territories examined and each territory contains genes with heterochronies of opposite sign. Given our current understanding of interactions within the dGRN, there is no obvious way in which one or two changes near the top of the dGRN could account for all or even most of the observed heterochronies. Third, the same gene can be heterochronic in one territory but not in another (e.g., *onecut* is maternal in both and later heterochronic in ectoderm). This observation is fully consistent with the dGRN model, in that distinct sets of transcription factors typically regulate transcription of a given gene in different territories and/or at different times, allowing for cell type-specific changes in the timing of expression.

### Heterochronies in the dGRN are not biased with regard to evolutionary branch

To learn more about the evolutionary history of the heterochronies, we turned to a third species, *Paracentrotus lividus* (*Pl*). This species is phylogenetically slightly more distantly related than *Sp* and *Lv* are to each other, providing a useful outgroup for comparison. We drew on a qPCR dataset from *Pl* that includes 22 of the dGRN genes [38]. Because *Pl* develops at an optimal temperature that is different from *Sp* and *Lv*, we again normalized developmental time to *Sp* developmental time (based on the normalized estimations of that dataset with *Sp* [38]. Additional file 8: Table S4 shows a comparison of normalized earliest expression for the 22 dGRN genes in the *Pl* study along with the same genes in *Sp* and *Lv*. One gene (*blimp1b*) was excluded because it is maternally expressed in *Lv and Sp,* and while *blimp1b* has a higher level of initial expression in the *Pl* dataset relative to the other 21 genes, the authors do not indicate it to be maternally expressed. Of the remaining 21 genes, 9 are heterochronic in at least one species. We used a simple parsimony model to infer the polarity of gene expression differences in these cases. Of the 9 heterochronic genes, 2 were uniquely different in *Sp,* 2 uniquely different in *Pl,* and 5 uniquely different in *Lv.* Thus, at least for this subset of dGRN genes, evolutionary changes in the timing of expression appear to be distributed more or less randomly, as might be expected.

### Heterochronies necessitate topology updates to the dGRNs of *Sp* and *Lv*

**Fig. 4:**
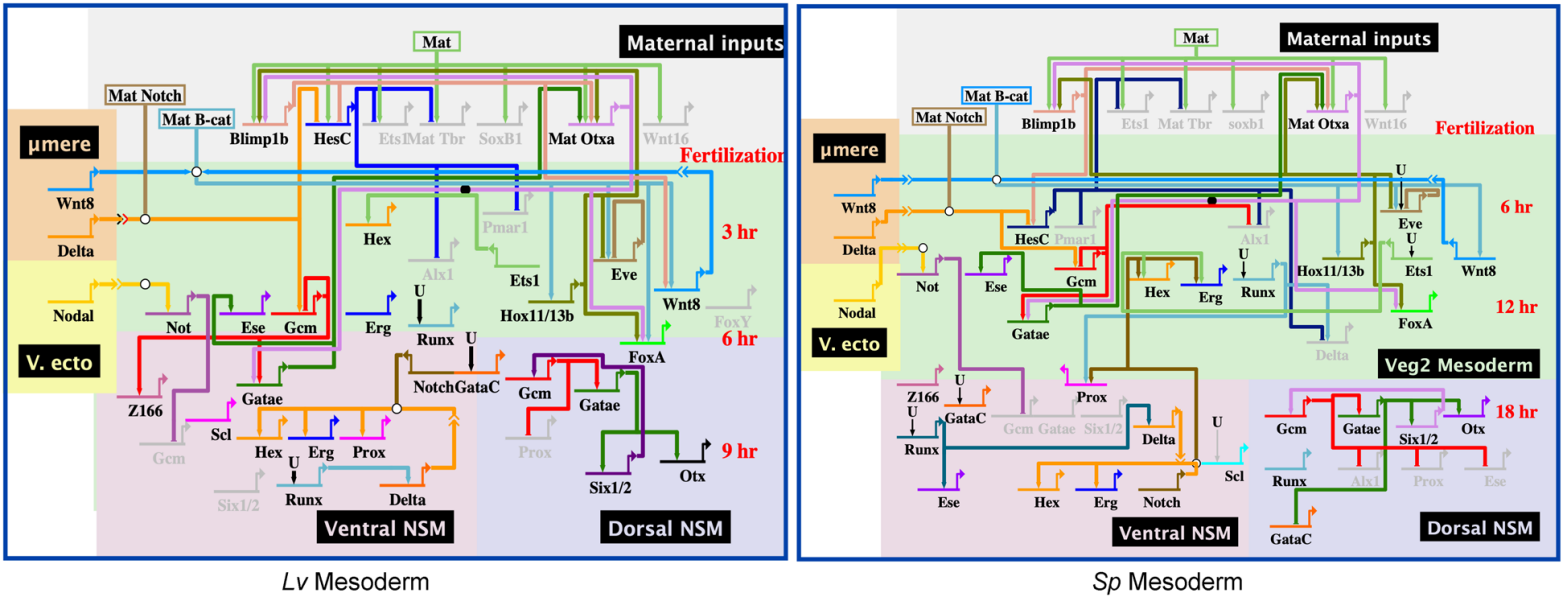
Mesodermal dGRNs of *Lv* and *Sp* reflecting time of first expression. Edges (positive or negative inputs to other genes) are unchanged from the large bank of perturbation data that established the dGRNs. Time of development is shown in red to the side of each dGRN. Maternally expressed genes are in the gray area at the top. Early specification is in green, and later the mesoderm is subdivided into ventral and dorsal GRNs as a consequence of Nodal signaling [62]

The discovery that some heterochronies in gene expression alter the order of first expression for genes that share an edge (i.e., interact) prompted us to update the topology of dGRN models so that *Lv* and *Sp* models could be compared. Figure 4 shows the updated mesoderm dGRN models for *Lv* and *Sp* with placement of the genes based on timing of first expression (top of GRN = earliest) with all connections retained according to the original dGRNs. Additional file 3: Fig. S3A-C shows similarly updated dGRN models for ectoderm, endoderm, and skeletogenic cells. Within these dGRNs a number of functional subcircuits exist including feedback inputs.

**Fig. 5:**
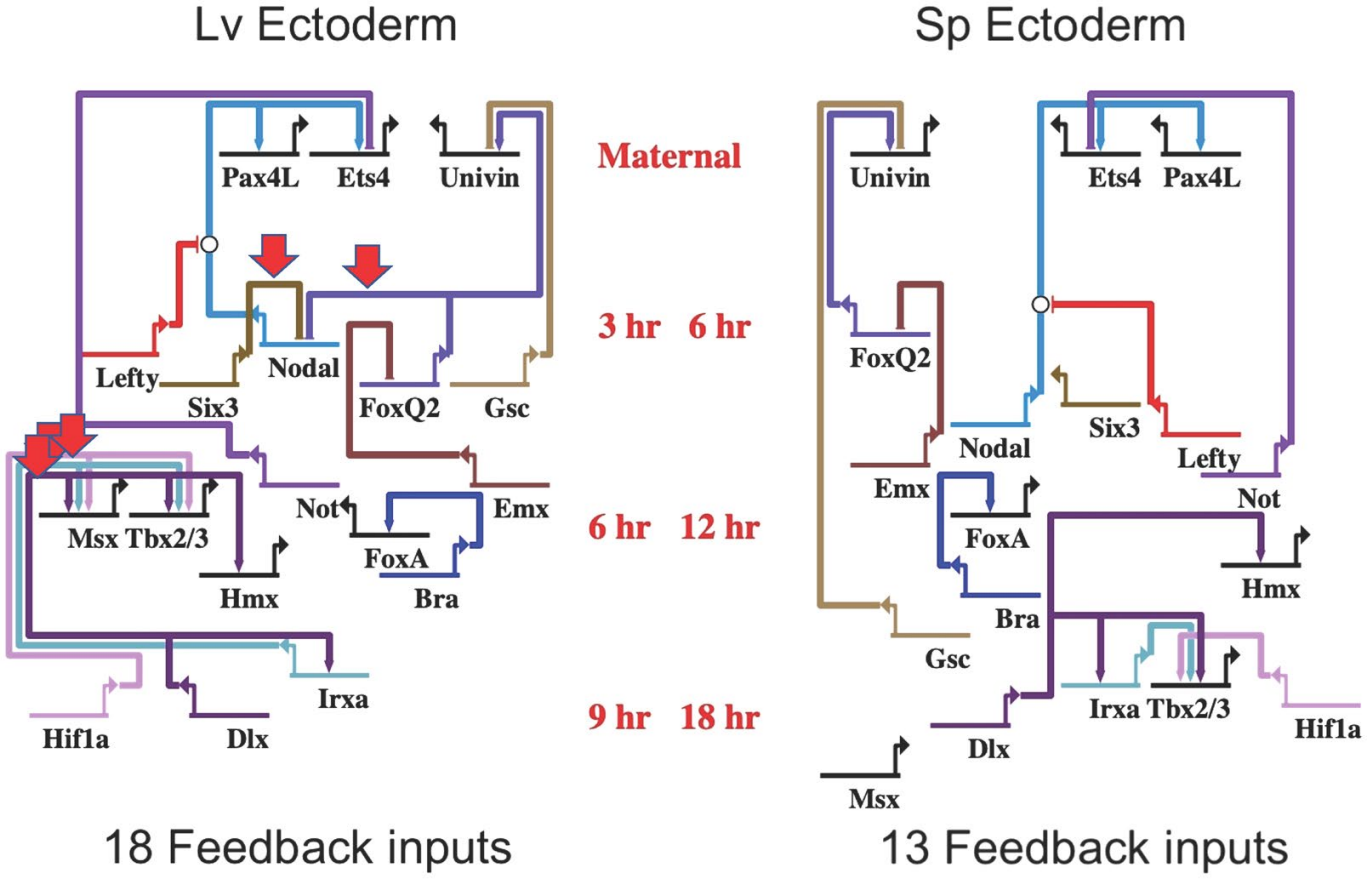
Inferred feedback inputs in *Lv* and *Sp* ectoderm during the first 9 and 18 h of specification. Based on time of first expression recorded in Additional file 5: Table S1, the feedback inputs emerged for every gene that had an experimentally based input into the regulation of a gene first expressed *earlier.* In comparing the two species most feedback inputs are conserved (13/18) though several feedbacks differ because of heterochronic shifts in gene expression (red arrows). Heterochronic shifts in expression of *foxQ2, six3,* and *msx* account for a difference of 5 feedback inputs between the two ectoderm dGRNs

We paid special attention to the feedback circuits because of the well understood stabilizing influence they provide to circuits of all kinds [8, 11, 12]. Figure 5 shows just the inferred feedbacks in the updated ectoderm dGRN model in both species. A number of these were previously published in dGRN models (e.g., negative feedback from *not* to e*ts4* and positive feedback from *bra* to *foxA* in ectoderm). Heterochronic shifts in expression of *foxQ2, six3,* and *msx* account for a difference of 5 feedback inputs between the two dGRNs. Other inferred feedbacks were not noted in previous dGRN models (e.g., positive feedback from *nodal* to *ets4* in ectoderm, here inferred to be present in both species). Additional file 4: Figs. S4A-C show the inferred feedback inputs for the other embryonic lineages of *Lv* and *Sp*. Altogether, there are 50 feedback inputs in the updated *Lv* dGRN models between fertilization and gastrulation, and 44 in the updated *Sp* dGRNs (Additional file 9: Table S5). In contrast, the earlier *Sp* GRN models contained a total of 24 feedback inputs. Even the expanded number of feedbacks in the updated dGRN models is very likely an undercount for both species, since the networks are incomplete.

Some interesting general results emerge from these updated dGRNs. First, the 44 feedback inputs of *Sp* and the 50 of *Lv* represent about 28% of all the connections in the two dGRN models. Second, the feedback inputs are distributed across different embryonic territories in both species, with proportions among territories fairly consistent between species (Additional file 9: Table S5). Interestingly, the territories containing a greater variety of cell types also contain more feedbacks. Together, these observations suggest that a moderately high proportion of interactions within the dGRN involve feedback circuitry and these occur in multiple cell lineages of the embryo. Third, positive feedbacks outnumber negative feedbacks by about 2–3 times in both species (Additional file 7: Table S3).

## Discussion

These data support the notion that GRNs are internally stabilized even though over evolutionary time temporal changes in gene expression occur. How those changes spread through the population and are subject to selection is not known though many possibilities exist in terms of establishing better fitness. At the network level, the stabilization can result from a number of factors and here it is shown that a larger number of stabilizing feedback inputs exist than had been appreciated previously.

We chose to define a heterochrony as a difference of greater than ± two hours in first expression of a gene when comparing two species. A 2003 paper by Bolouri and Davidson helps explain the necessity of including a generous variation in normal timing of first expression [54]. Enhancer occupancy of a downstream gene by a transcription factor depends on a number of molecular events beginning with the regulatory activation of that upstream transcription factor. Initiations per unit time vary as do several steps in post-transcriptional processing. Accumulation of protein products varies depending on the number of mRNAs available for translation and the protein turnover rate. Consequently, there is no canonically specific step time between the initial activation of the upstream gene and the initial activation of the downstream gene whose expression is controlled by the upstream gene. Other variables affect each repeat of the same experiment. Consequently, it is necessary to allow for that variation. Here we chose a 4-h window, which is about 15% of the time from fertilization to the beginning of gastrulation in *Sp*. This is arbitrary but we believe is a conservative estimate of natural variation in dGRN activity*.* In the Bolouri and Davidson paper, an average step time for *Sp* was estimated to be three hours, or roughly 9 regulatory steps allowed in GRNs between fertilization and gastrulation. Species differences of greater than ± 2 h are considered to be heterochronies. Figure 3 illustrates that 61/81 genes fit within the ±2-h window and 20 (24.7%) of the genes are heterochronically activated in the two species comparison.

Additional variables could exist but the nature of transcriptional control limits those. For example, suppose a downstream mRNA accumulates to a very high level while its controlling transcription factor is relatively rare. The possibility exists that the downstream gene is misinterpreted as being initiated prior to the upstream gene. However, this is unlikely since it is the initial accumulation of the upstream transcription factor and not the steady state level of that factor that matters in step time control [54]. The earlier study comparing 25 genes from *Pl* with the same genes expressed in *Sp* is also pertinent here [38]. That study carefully measured times of first expression of 25 dGRN genes in *Pl,* and also followed the dynamics of expression and compared those expression profiles between *Pl* and *Sp.* They found some differences in relative time of first expression but the dynamics of expression over time were quite similar. Again, as concluded earlier, even though the general temporal profiles were similar, the Bolouri and Davidson analysis indicate an important consideration is the early events of regulation following time of first expression (54). The Gildor and Ben-Tabou de Leon study was a valuable resource for the comparison made here between *Sp* and *Lv* since 22 (of the 25) genes in their dataset could be compared with *Lv*. This enabled a parsimonious likelihood call as to where a heterochrony likely arose. And as might be expected, some heterochronies likely arose in each of the three species. Gildor and Ben-Tabou de Leon later showed that heterochronies are present in a comparison between sea stars and *Pl* separated by about 500 million years since a common ancestor [55], and we showed evidence of heterochronies in a comparison of *Lv* with a cidaroid species separated by about 200 million years [56]. In each of those cases other evidence also shows a partial remodeling of the dGRNs [57, 58]. The degree to which heterochronies contributed to the network remodeling is unknown but their presence in both cases at least suggests that they may be a factor that buffers a GRN from change for a long period of time, and oddly enough, could contribute to the eventual evolutionary change when it occurs as below.

Returning to the question raised in the introduction, how is it possible for GRNs to remain so stable for millions of years yet occasionally undergo changes that revolutionize development? We gave the example of the two *Heliocidaris* species that are separated by only about 5–10 million years from a common ancestor yet one develops indirectly in the same way that *Sp, Lv,* and *Pl* develop, while the other, (*Heliocidaris erythrogramma (He),* has no micromeres, and reaches the juvenile stage in three days without going through a planktonic larval stage. Though less well studied, the dGRN of *H. tuberculata (Ht)* seems very similar to the *Lv/Sp/Pl* dGRNs, while the dGRN of *He* is very different from that of *Ht.* One possible way a dramatic change in dGRNs could happen in a relatively short evolutionary time period could be that accumulations of heterochronies, absorbed by dGRNs due to stabilizing feedback inputs and other mechanisms, nevertheless strains the network at one or more nodes in the network over evolutionary time. These strains could be tolerated for long periods of time because of the internal stabilizing features of the network until a very unlikely mutation event occurs that dramatically changes the dGRN. That mutation could provide an opportunity to relax the strains allowing more new connections than would otherwise occur with that single mutation. Of course, this is speculation, but how might that have happened with *He* in its divergence from *Ht?* The egg of *He* is huge relative to *Ht,* the egg of the planktotrophic species. The increase in the size of the *He* egg likely occurred due to selection over an extended time under conditions where nutritional resources for adults were adequate but resources for embryos were limited. As the egg increased in size a number of other changes had to occur. These include possible changes in concentration of maternal proteins and/or mRNA, altered timing of cell cycles, alterations in cell number before zygotic expression was initiated, changes in localized deposition of maternal factors, and/or other changes, any one of which could augment stress on the dGRN. The switch from planktotrophic to lecithotrophic need not have occurred all at once, and indeed there are extant examples of facultative lecithotrophs [59], which, although rare, exhibit changes in direct vs indirect development that correlate with egg size. Preliminary evidence indicates that the structure of the *Ht* and *He* dGRNs are significantly different from one another. One way that could occur in the relatively brief time separating these two species evolutionarily, is a series of silent changes (including heterochronies) that accumulate in the dGRN over time that helped revolutionize how *He* develops once the switch from indirect to direct development occurred.

The 81 genes in this analysis are only a small part of the control system driving early development of the sea urchin. There is little doubt that many additional temporal differences between *Lv, Sp,* and *Pl* are present and yet the adults of the three species closely resemble each other despite about 50 million years separation. Network structure begins with cis and trans regulation of transcription factor expression. Changes in either have an impact on GRN structure. As the networks assemble during development many other factors contribute. Here we show that a number of stabilizing feedback subcircuits exist during early development. Recently, another analysis of the same GRN models suggests that stabilizing circuits or “kernels” (many of which include feedback), are included as part of GRN structure and it is proposed that these also contribute to stabilization and conservation of GRNs by providing efficient circuit structures to accomplish a network “job” [60]. That job might be to assure activation of a suite of necessary genes at a given time. It could be a circuit that leads to a necessary divergence in the network inherited by daughter cells, or it could be any of the many other jobs accomplished by a GRN.

While the heterochronic changes between *Lv* and *Sp* alter the architecture of the GRN models due to gene placement, that topology shift does not necessarily alter the relationship between the nodes. In other words, a temporal change in expression of Gene A in *Lv* might be heterochronic to the expression of Gene A in *Sp,* but the relationship gene A to gene B could remain the same (e.g., gene A is an upstream activator of B in one species but provides a positive feedback input to Gene B in the other species). There were 11 cases where gene A is upstream of Gene B in one species (red arrows in Fig. 5 and Additional file 4: Figs. S4A-C), but provides a feedback input of the same sign to Gene B in the other species. In each of these latter cases there were earlier inputs into Gene B indicating that genes other than Gene A were the initial activators, and when the heterochronic event occurred, the Gene A feedback input provided an expression maintenance, booster or repression function.

## Conclusions

This analysis reports on dGRNs of *Lv* and *Sp,* two species that have been extensively studied. Those perturbation analyses provide a strong measure of confidence that the known node connections between the two species are correct. While many of the connections could actually be indirect given that cis-regulatory analyses are incomplete, the data presented here support the probability that many of those inputs lead to a feedback function. Additionally, the data reveal that a number of heterochronic changes have occurred in networks over time, even though the function of those networks remains conserved for extended periods.

## Methods

### Embryo spawning and culture

Adult *Lytechinus variegatus* sea urchins were spawned by injecting 1 ml 0.5 M KCl intracoelomically. Unfertilized eggs were washed in artificial sea water (ASW), resuspended, and fertilized by a single male’s sperm. Following fertilization, eggs were washed in ASW to remove excess sperm and reared at 23 ˚C in glass dishes at low density. Large embryo batches were cultured in beakers with constant stirring.

### RNA extraction and qPCR measurements

Embryos at each sampled timepoint were lysed using a tissue homogenizer, and RNA extraction was performed using a PureLink RNA Mini Kit (Invitrogen, Cat. #12183018A). RNA quality was assessed using a Nanodrop. Genomic DNA contamination in the RNA samples was removed using a TURBO DNA-free Kit (Invitrogen, Cat. #AM1907). The RNA from each sample was reverse transcribed into cDNA using an AffinityScript cDNA Synthesis Kit (Agilent, Cat. #600,559). cDNA quality was assessed using a Nanodrop.

Real-time qPCR was used to assess expression levels of 19 key GRN genes during early *Lv* development (see table below for primers and genes). The assays included three replicates for each timepoint listed above. qPCR amplification reactions were run on a LightCycler 96 system (Roche) using a QuantiNova SYBR Green PCR Kit (Qiagen, Cat. #208,054) in 96 well PCR plates. Each 20 µL reaction contained 10 µL of master mix, 1 µL each of the forward and reverse primers (at 14 µM), 2 µL cDNA (at ~ 500 ng/µL), and 6 µL water. Sample plates were run in the thermocycler for the following cycle: 2 min heat activation at 95 ˚C, followed by 50 cycles of 5 s denaturation at 95 ˚C and 10 s combined annealing and extension at 60 ˚C.

Raw expression data from the qPCR runs were assessed using basic statistical tools in the R software environment.

### Primers for qPCR

**Table:** 

Gene name	Forward primer	Reverse primer
Ets	ACTATGAGAAGCTGAGCCGC	TTGCCGAGTTCTCAGTCGTC
Eve	TGGACTCGACAACCACTTCG	GACGACAAGCCCTATACCCG
FoxA	TTCATCCTGACGCTGGCAAT	CGTTAGGGTCTCCGTTCTCG
FoxB	TGAGCGCCCGACAAACTTAT	TTTACTGCCTTGGCCTCTGG
GATAc	CATGTTCCTGCGGCTTATGC	ATTCTCGGCCCTCTGTTGTG
Gcm	TCACAGCCTCACAAGACGAC	TGCCATCGTACCCGTAATCG
Gsc	CGGATTCTGACGGGCTAGAC	GTGAACGGGATAGCGAGAGG
Hnf6	CGAAAGAAATCGCAGCGAGG	ACTTCCACATCCGCCTGAAG
Blimp1b	GATTGCATCCCGACCTCGAT	CGGAAAATCCTTGAGGGGCT
Msp130	TGTTGGAGGTCGTCGACTTG	TTTAGCGAGCCAACATCCGT
Nkx2.1	GCCACGCAATCCATGACATC	GCTTGGCTTTGAGGGTGTTG
Nodal	GACATGGCAGACCTCCTTCC	ACGGGTGGTCGAAGGTAGTA
Not	TAAATTCCACCTGCCCGCTT	GGCGATGGATGGAAGACGAT
Pks1	TCTGCATCACACACCAGGAC	ACGCACAAGAGGTCTCCAAG
Pmar1	CCGTCGAGCCTCTCTTGTTT	CACGACGCCCAACTTCTTTG
SoxB1	TCTATCCCAGGCATGACCCA	TCCTGCGTTGTCCTCTTGAC
SoxE	GACAAGGAGAAGCAGCCCTT	CAAGGTGGAGCGGTTGTTTG
Tbr	AGGCGTCAGTTTACCTCTGC	CCACTGGTTCGGATCACACA
Wnt8	TCAGCGGGAAACTCATCGAC	CTGATGCCGATGGTCAGGTT

### Identifying time of first zygotic expression

Expression data shown for *Sp* in Additional file 7: Table S3 are taken from [37]. Those data were generated on the Nanostring nCounter platform and consist of hourly time points from fertilization to 48 hpf. Time of first zygotic expression was considered to be the hour at which an upward inflection of more than 10% occurred relative to background, and continued in successive timepoints. Background counts ranged from about 2% to upwards of 30% of the final level of expression, depending on the gene. For background, any count larger than 3% of the highest level of expression of a gene was considered to be maternal expression by the authors.

Data for *Lv* were taken from [36]. These data were generated on the 10X Chromium scRNA-seq platform and consist of hourly timepoints from 2 through 16 hpf, as well as 18, 20, and 24 hpf. Waddington-OT [39] was employed to optimize lineage trajectories thereby assisting in identifying time of first zygotic expression in each of the included lineages. Time of first zygotic expression was considered to be the time point at which more than 10% of the cells in a lineage at that time expressed the gene in question and was exhibited as an inflection point with later time points in that lineage continuing to rise (Additional file 1: Fig. S1, Additional file 5: Table S1). Genes expressed above background at the 2-h timepoint are scored as maternal. This approach was supported by qPCR measurements of 18 of the genes in the dataset. Each scored inflection points within an hour of the scRNA-seq approach (Additional file 6: Table S2). One gene in that dataset was not used (*pks1*) because its background suggested maternal expression. Another (*Blimp1b*) is maternally expressed in both *Sp* and *Lv,* and later both have an inflection point within 2 h of each other. Expression data for *Pl* were taken from [38]. Those data were generated as bulk qPCR measurements taken at 1–2 h intervals from fertilization to 30 hpf. Time of first zygotic expression was considered to be the time point at which a sigmoid function of the data increased using spiked known amounts of GFP as the control [38].

Adults of the three species studied here occur in disjunct ranges and different water temperatures, so laboratory cultures are reared at different temperatures to ensure normal development. For the data sets used culture temperatures were: *Sp* 15˚C, *Lv* 23˚C, and *Pl* 18˚C. Thus, it was necessary to correct for temperature differences during analysis. In what follows, *Lv* and *Pl* development times were both converted into *Sp* developmental time to allow for direct comparison. Prior experience indicated that *Lv* develops about 2X as fast as *Sp* at normal rearing temperatures (cleavage times, mesenchyme blastula stage and initiation of invagination of the archenteron occur in half the time in *Lv* at 23 °C relative to *Sp* grown at 15 °C). The scatterplot based on this conversion shows that most times of first zygotic expression lie close to a line of slope = 1 (Fig. 3), indicating that a 2X rate correction is fairly accurate overall. Times of first zygotic expression for *Lv* were therefore multiplied by 2.0. Growth rate of *Pl* relative to *Sp* used the calculations from [38] where it was concluded that the overall rate difference between *Pl* and *Sp* as 1.3X, so times of first zygotic expression for *Pl* were multiplied by 1.3. The dataset from *Pl* was the smallest of the three so for comparison purposes the genes in that dataset were compared against the same genes from *Sp* and *Lv.* Times of first zygotic expression for all species in *Sp* developmental time are shown in Additional file 8: Table S4.

### Identifying expression heterochronies

Based on Additional file 7: Table S3 and Fig. 3, time of first zygotic expression is compared between *Sp* and *Lv*. Genes of the two species initially expressed within ± 2 h of each other are considered to be expressed at the same time with the ± 2 h used to account for batch variation, or variation in sensitivity to detection by either nanostring, scRNA-seq or qPCR. As seen in Fig. 3, allowing for the variation resulted in 60/81 genes in the dataset to be similar in time of first expression. Comparative differences in time of first expression of a gene that were greater than 2 h (20/81 genes) are considered gene expression heterochronies.

### Inferring feedback inputs in the dGRN

The dGRNs of *Sp* and *Lv* were identified over many years based on a large number of perturbation experiments. The general approach involves knocking down gene A and measuring the expression of gene B (Fig. 1). If expression of gene B is reduced or eliminated, gene A is likely directly or indirectly necessary for activating expression of gene B (Fig. 1); alternatively, if expression of gene B increases (with gene A knockdown), gene A is considered to directly or indirectly repress expression of gene B. If the expression of gene B is not affected, there is likely no direct or indirect interaction between them (more specifically, no interaction that influences the expression of gene B at the developmental times measured). Carrying out additional perturbation experiments and applying this straightforward logic expands the network of interactions and genes modeled by the dGRN. One element of inferring network topology, however, was not always considered in prior studies, however, namely the possibility that gene A might act through positive or negative feedback. Careful attention of the time of first zygotic expression can identify such cases. Consider a situation where gene A affects the expression of gene B, but gene A is first expressed *after* gene B. In such cases, gene A must provide a feedback input to the earlier expressed Gene B; furthermore, the initial activator of gene B must be a gene other than gene A (Fig. 1). Both positive (maintaining or boosting) and negative (repressing) feedback inputs can be inferred using this logic. Based on updated times of first zygotic expression, this criterion was applied to the dGRNs of *Sp* and *Lv* to infer positive and negative feedback inputs and for likely origin of heterochronies. Data from *Pl* were incorporated to infer likely origin of the heterochrony. Where the time of first zygotic gene expression was similar in *Pl* and *Sp*, the change in timing, if present, was inferred to have occurred on the branch leading to *Lv* (*e.g.*, *FoxQ2*). Conversely, where *Pl* and *Lv* show similar first expression, the change in timing was inferred to have occurred on the branch leading to *Sp* (*e.g.*, *Hex*). Expression data for *Pl* are available for fewer dGRN genes than the other two species, so this comparison was only possible for 22 genes.

## Supplementary Information

**Additional file 1: Figure S1.** Data used for selecting time of first expression of *Lv* dGRN genes. Each of the 81 genes in the dGRN were graphed at each of the 19 time points of that analysis (X axis) Each graph provides the cells expressing that gene at each hour (each cell = dot), and the level of expression by each cell (Y axis). The table was constructed by measuring all cells at each time point since that was the nanostring approach used in the *Sp* analysis. Further refinement of this method is possible for each lineage but not reflected here. The times selected as earliest times of expression are given in Additional file 5: Table S1.

**Additional file 2: Fig. S2 A**. The original *Sp* dGRN model of skeletogenic cells and an updated version reflecting time of first expression. The updated version on the right has been simplified by removing genes that have not been independently verified and the several differentiation genes are combined. **B**. The original *Sp* dGRN model of endomesoderm and an updated version reflecting time of first expression. The updated version is shown on the right. Mat = maternal, U = unknown activator, Oral NSM (non-skeletal mesoderm) is considered the same as larval ventral NSM and Aboral NSM is considered the same as larval dorsal NSM. **C** The original *Sp* dGRN model of ectoderm and an updated version reflecting timing of first expression. The original *Sp* dGRN model [63](left) reflected a number of ectodermal territories. The updated timing dGRN model (right) is simplified to reflect only the dorsal and ventral regions of ectoderm that are subdivided as a consequence of Nodal signaling [51]

**Additional file 3: Fig. S3 A**. Ectodermal dGRNs of Sp and Lv redrawn to reflect timing of first expression. Earliest time points are at the top and normalized hours post-fertilization are indicated on the right side of the dGRN models. Maternal genes are in the gray area at the top of each dGRN. The light yellow area shows the generalized early expression of all ectoderm. The purple and light green regions show the subregions that are further specified following Nodal signaling that initiates those regional separations [51]. **B**. Endodermal dGRNs of *Sp* and *Lv* redrawn to reflect timing of first expression. Earliest time points are at the top and normalized hours post-fertilization is indicated on the right side of the GRN models. Maternal genes are in the gray areas. At sixth cleavage an equatorial division separates the veg1 endoderm (light orange) from the veg2 endoderm cells (light yellow). These two regions are then specified somewhat differently. Time of development for both species is in red to the right of each GRN model. **C**. Skeletogenic mesenchyme dGRNs of *Sp* and *Lv* redrawn to reflect timing of first expression. Earliest time points are at the top and normalized hours post-fertilization is indicated on the right side of the GRN models. Maternally expressed genes are in the gray area. The dGRN models are simplified to show inputs into multiple differentiation genes. The *Lv* skeletogenic mesenchyme model includes *snail* and *twist* which were identified and through perturbation studies included in that GRN model [64, 65]. Connections in the *Sp* GRN model are identical to the *Lv* model except *snail* and *twist* were not tested in *Sp*

**Additional file 4: Fig. S4 A** Feedback inputs in the updated *Lv* and *Sp* endoderm dGRNs. The diagrams show only the feedback circuits. The red arrows indicate feedbacks that are unique to one of the two species. **B**. Feedback inputs in the updated *Lv* and *Sp* mesoderm. The diagrams show the feedback inputs only in the two dGRNs. The red arrows indicate feedbacks unique to one of the two species. **C** Feedbacks in the updated *Lv* and *Sp* skeletogenic cells. The diagrams show the 10 and 7 feedbacks in the two species. The red arrow indicates a feedback input that is unique to *Lv.* Two other feedbacks in *Lv* are *snail* and *twist* inputs into *alx1*. These genes are not incorporated into the *Sp* dGRN models

**Additional file 5: Table S1.** Time of first expression of 81 dGRN genes in *Lv.* Shown are two time points for each gene, the first time point is the last hour of expression of background, and the second time point is the first upward inflection. **Fig. S1** illustrates graphs showing cells and level of genes expressed by those cells. All 81 genes were subjected to this analysis for time point selection**.**

**Additional file 6: Table S2.** qPCR of a selection of the dGRN genes. This table shows the 20 genes selected for qPCR and the Cq results at each hour. Highlighted in yellow are the times identified as approximating the inflection point of expression. These times are included in the comparison of **Table S3** and all are within an hour of the identified scRNA-seq inflection time points.

**Additional file 7: Table S3.** Comparison of Times of first expression of 81 dGRN genes in *Sp* and *Lv.* Data from [37] were used to identify time of first expression of *Sp* genes. Data from [36] were used to approximate time of first expression of *Lv* genes. A qPCR analysis provided an independent assessment for 19 of the 81 *Lv* genes. To normalize the times of first expression due to difference in temperature of culture, the time of first expression of *Lv* is 2X since *Lv* reaches each stage of development up to gastrulation in half the time needed for *Sp* to reach that same stage (see Methods).

**Additional file 8: Table S4.** Timing of first expression of three species reveals the likely origin of a heterochrony. Data from [38] for 21 genes with timing of first expression normalized to approximate *Sp* rate of development. Genes in red are expressed at times that differ in *Sp* relative to the other two species; yellow are genes uniquely different in *Pl;* genes uniquely different in *Lv.* Each colored is most likely the species in which the heterochrony originated

**Additional file 9: Table S5.** Summary of feedback circuits in the *Sp* and *Lv* dGRNs. The feedbacks recorded in the original *Sp* dGRNs are summarized as the “classic” models. After consideration of timing of first expression the dGRN models for *Sp* and *Lv* show significant increases the presence of positive and negative feedback circuits.

## Data Availability

Published data used is referenced and those references contain access to the data used. Additional data here are present in the supplementary information of this paper.
