# Supplementary figures and images for: Feedback circuits are numerous in embryonic gene regulatory networks and offer a stabilizing influence on evolution of those networks
Source: EvoDevo. 2023 Jun 16;14:10. doi: 10.1186/s13227-023-00214-y (PMC10273620; doi:10.1186/s13227-023-00214-y)

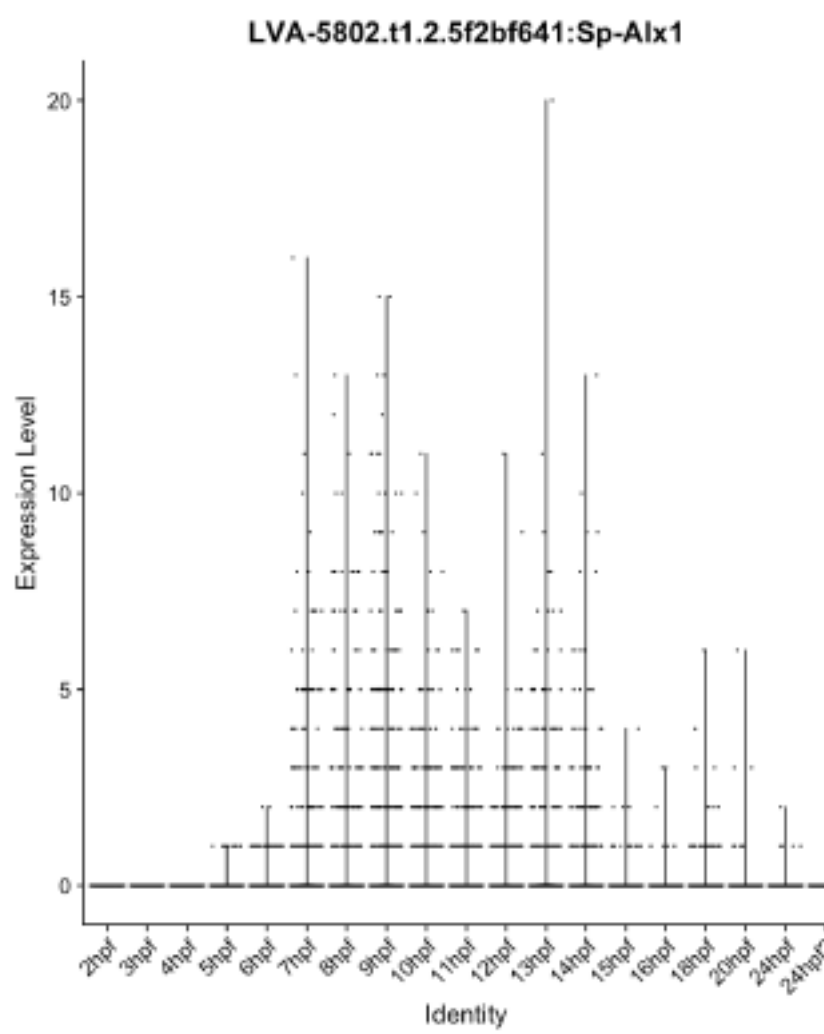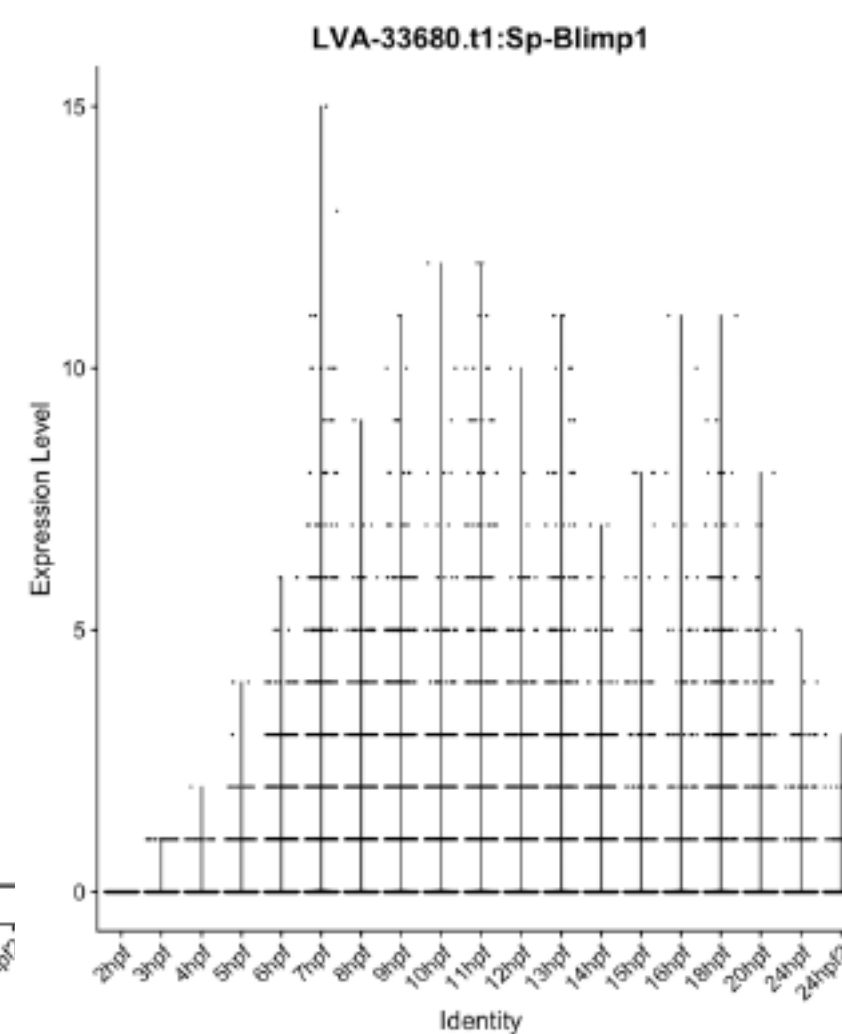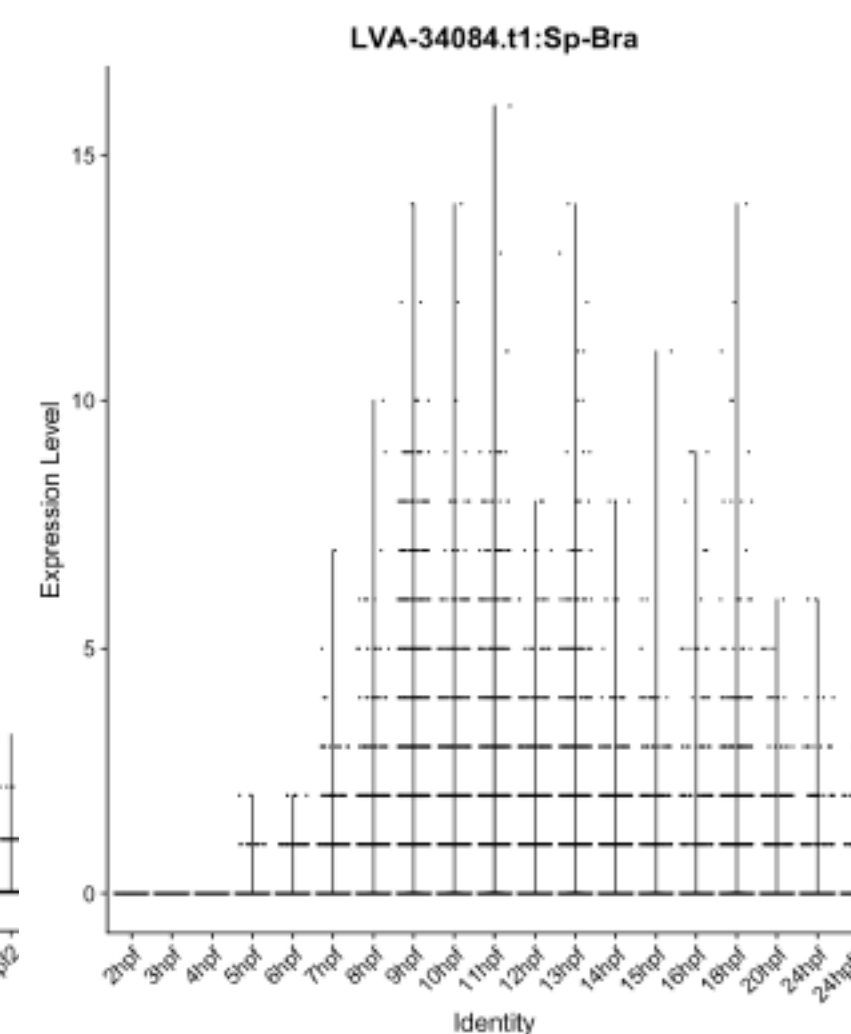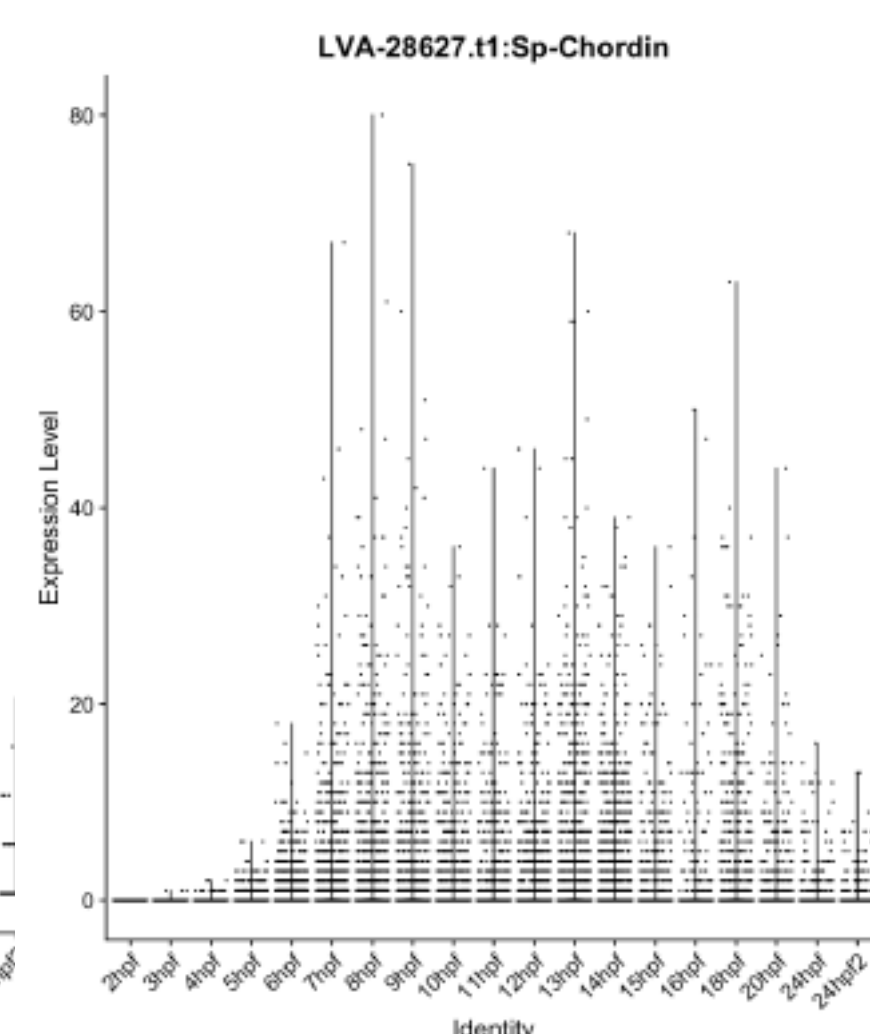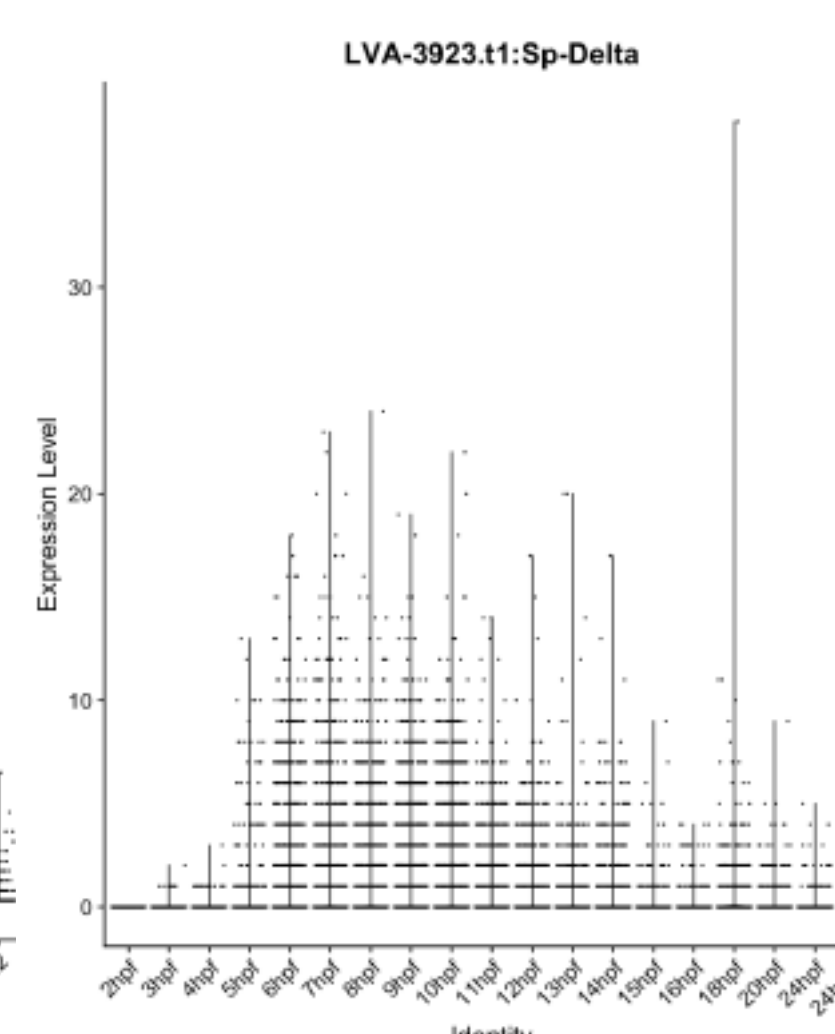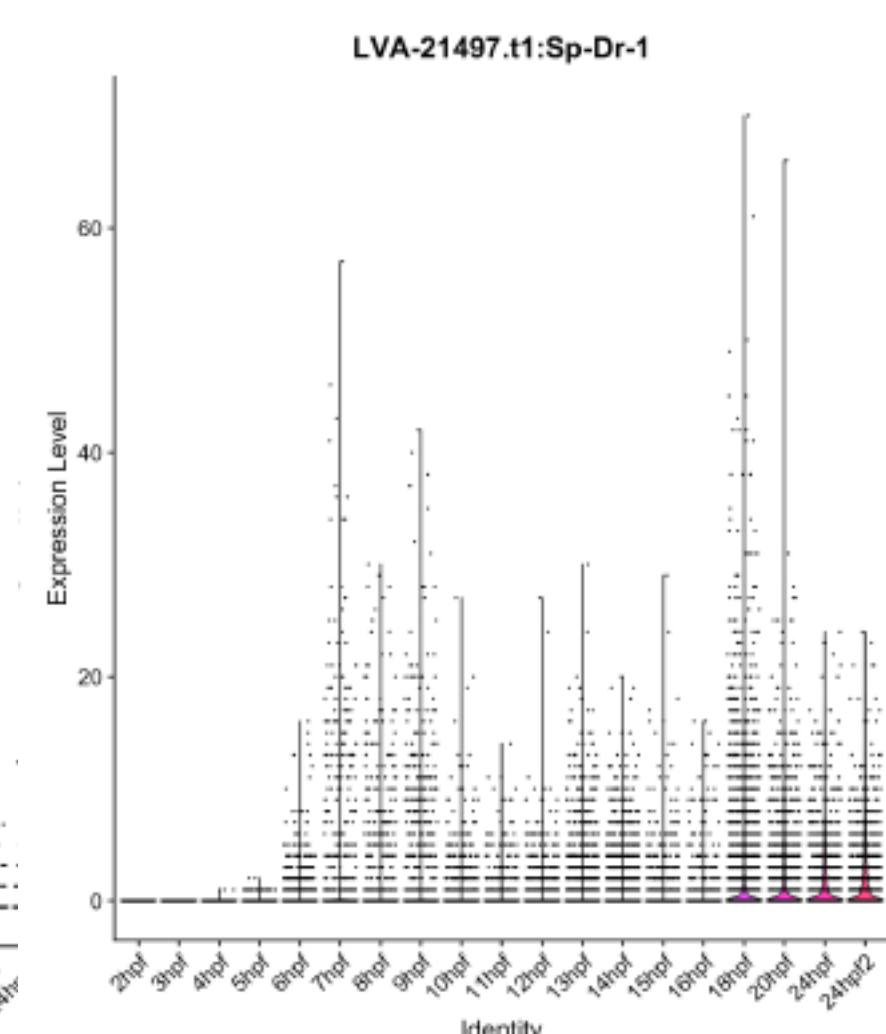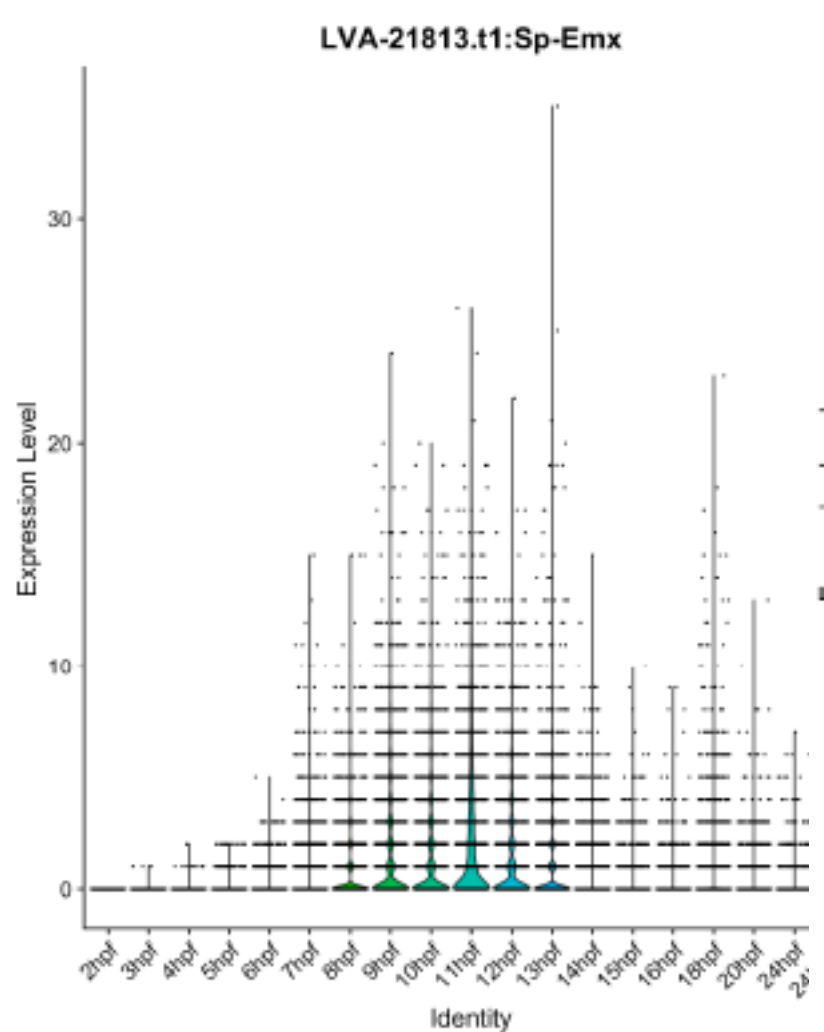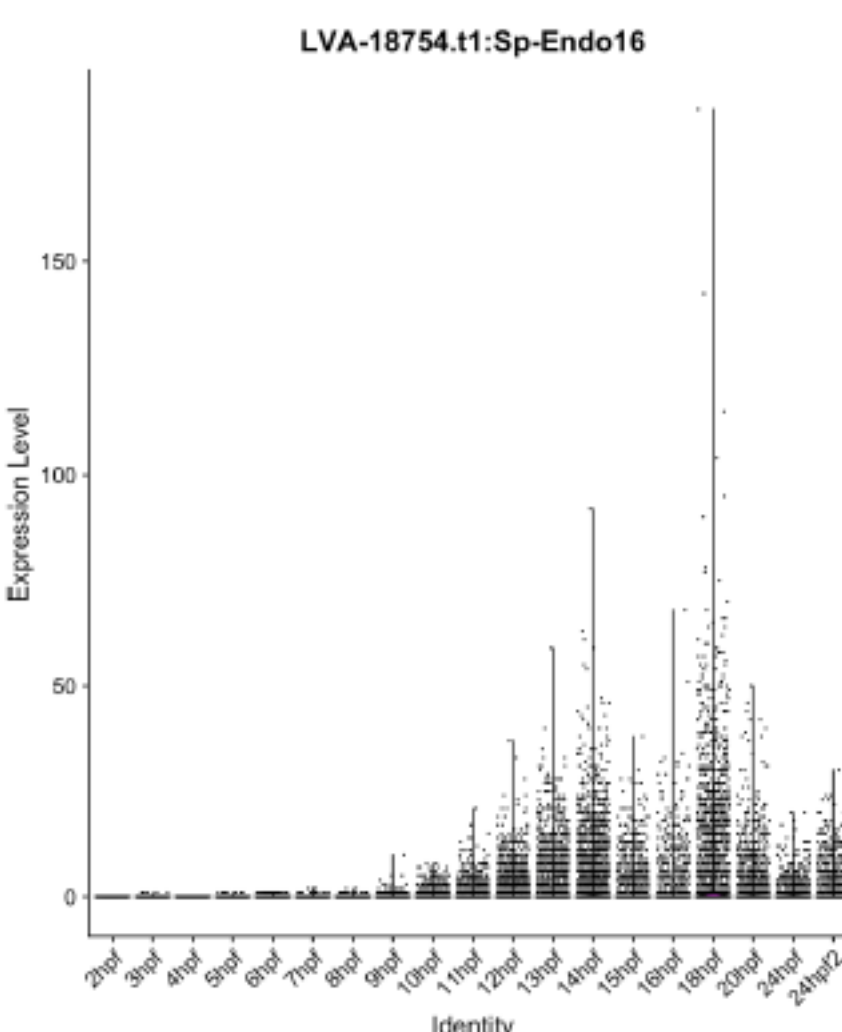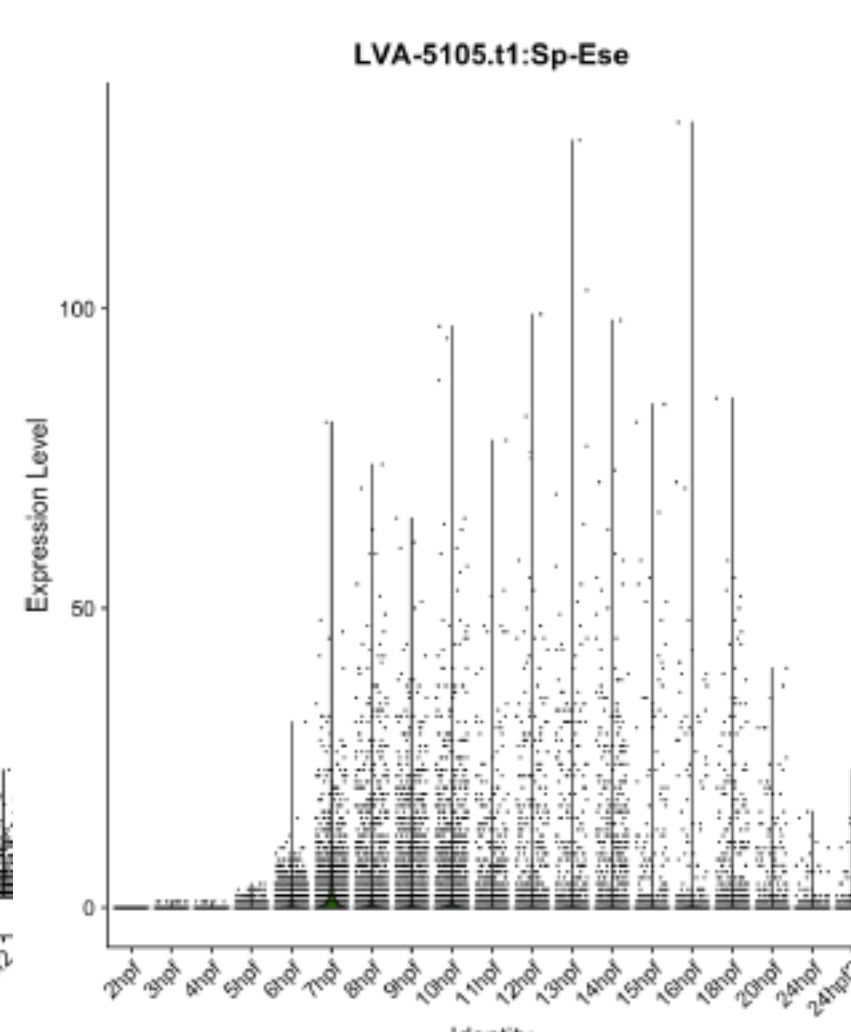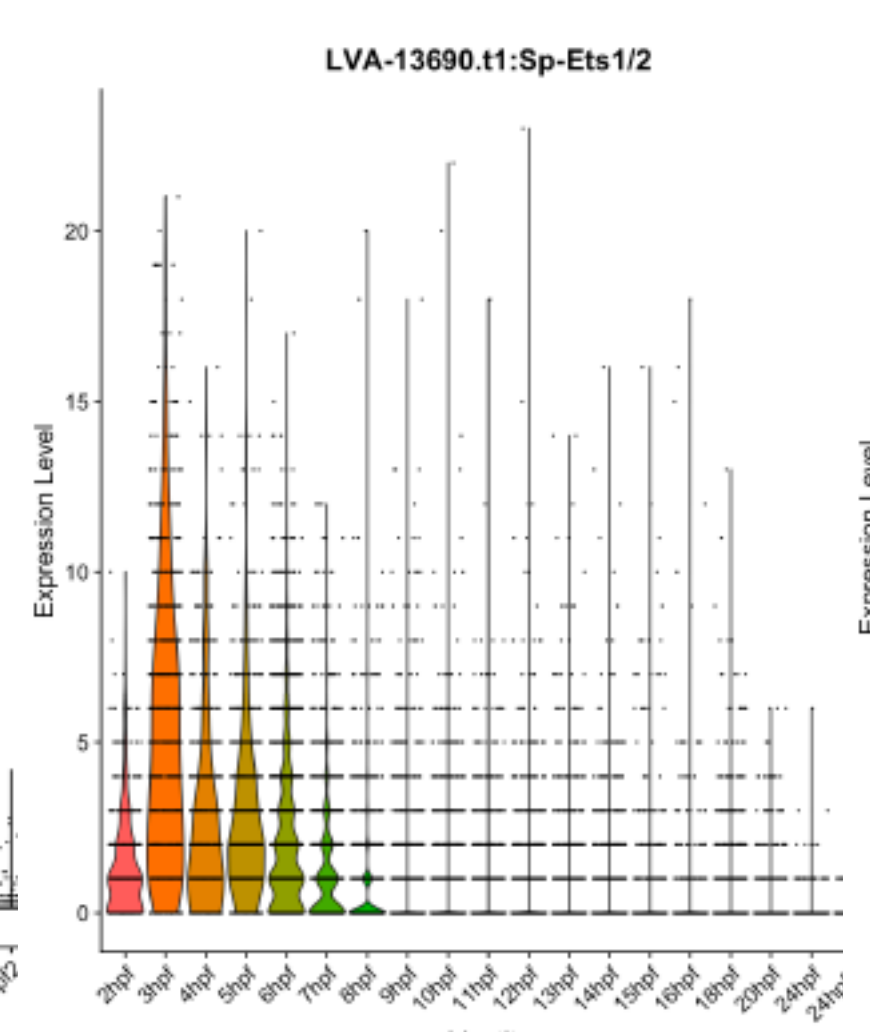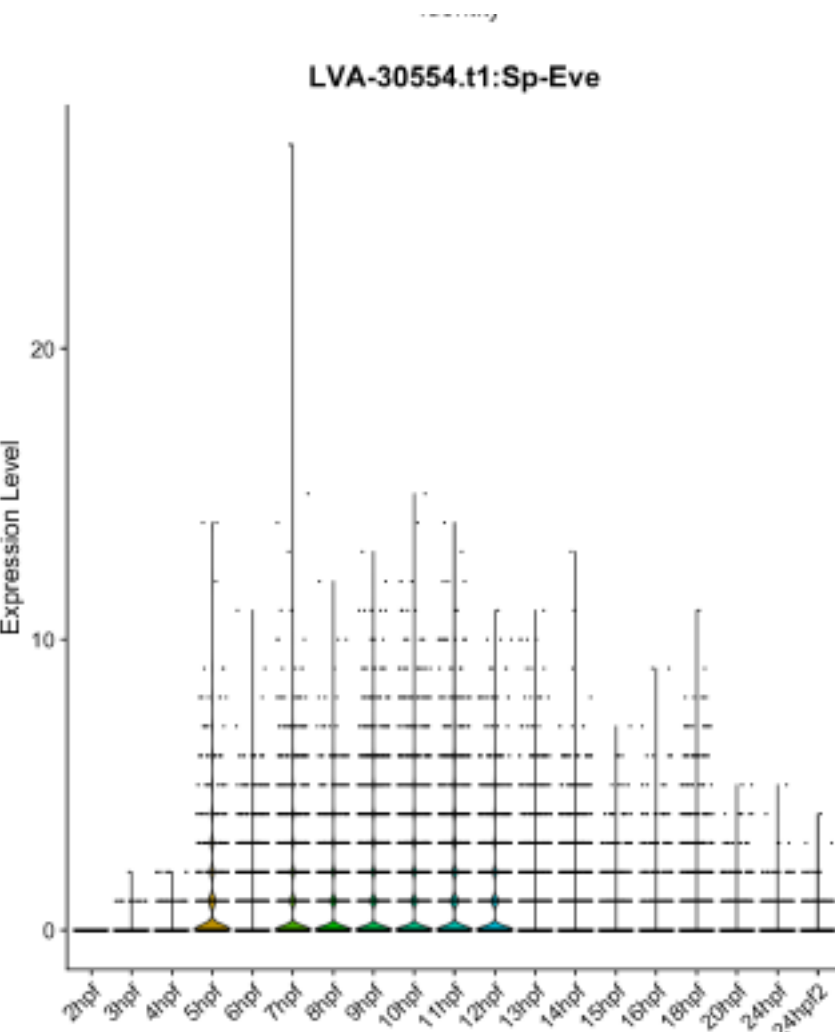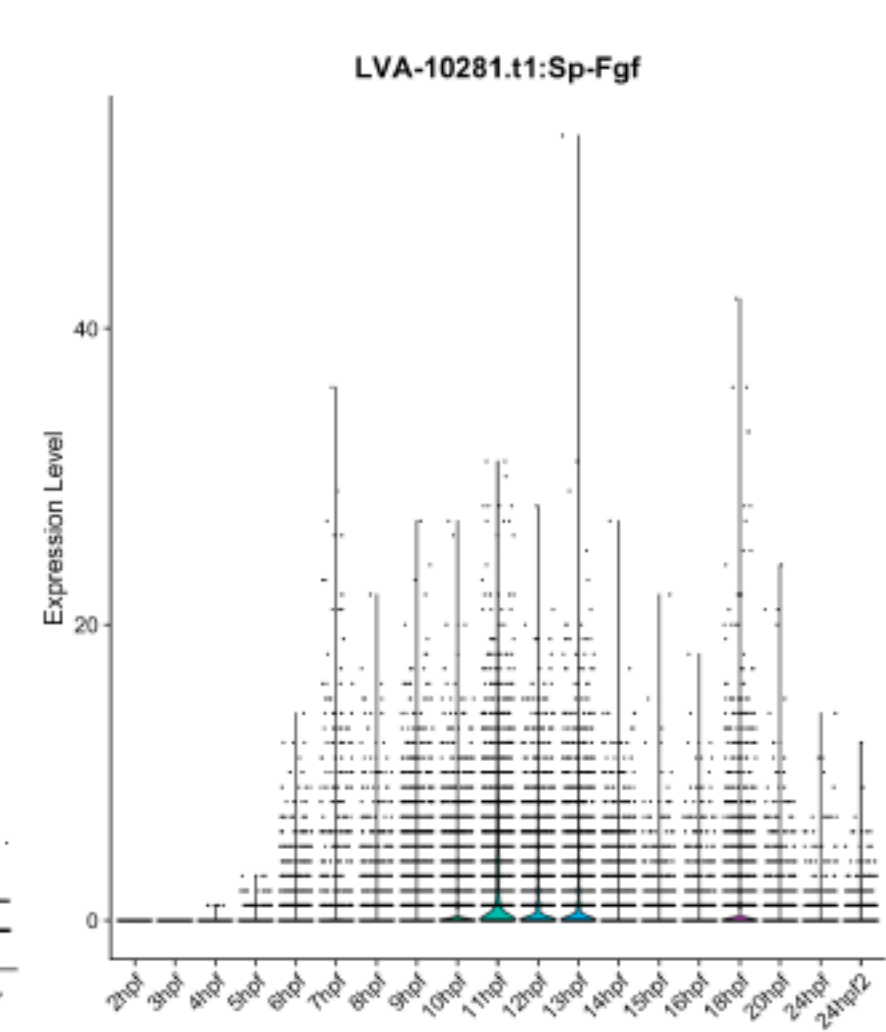

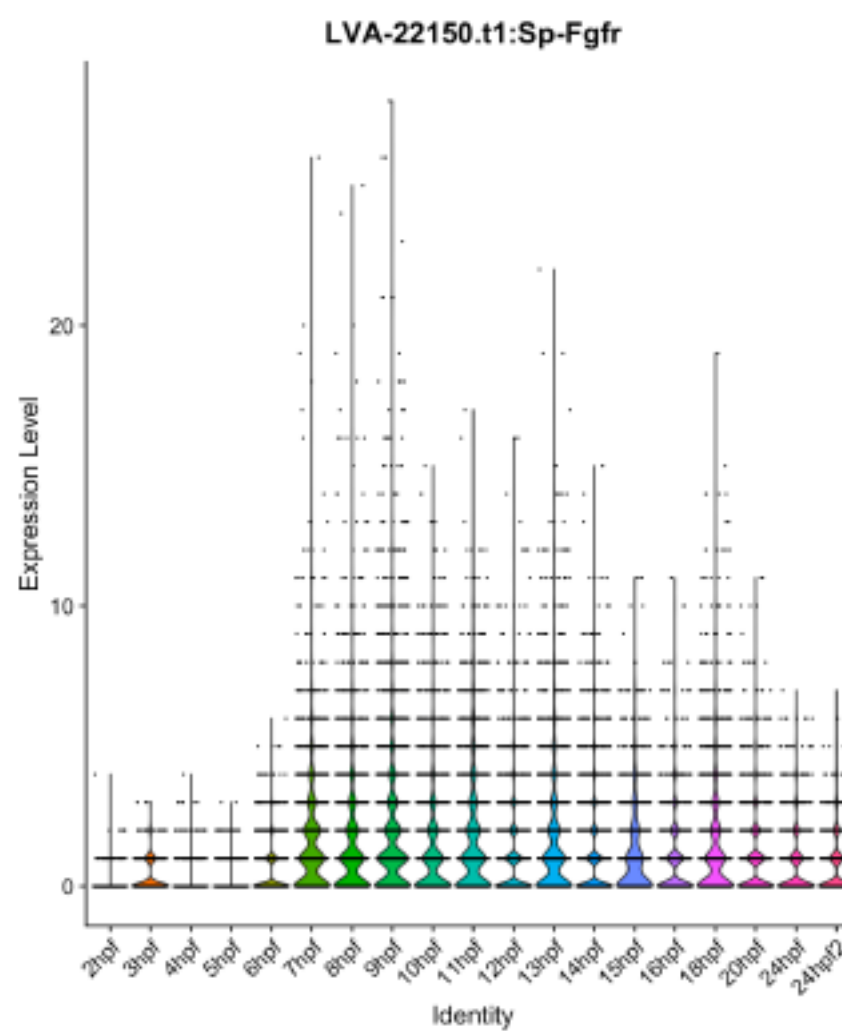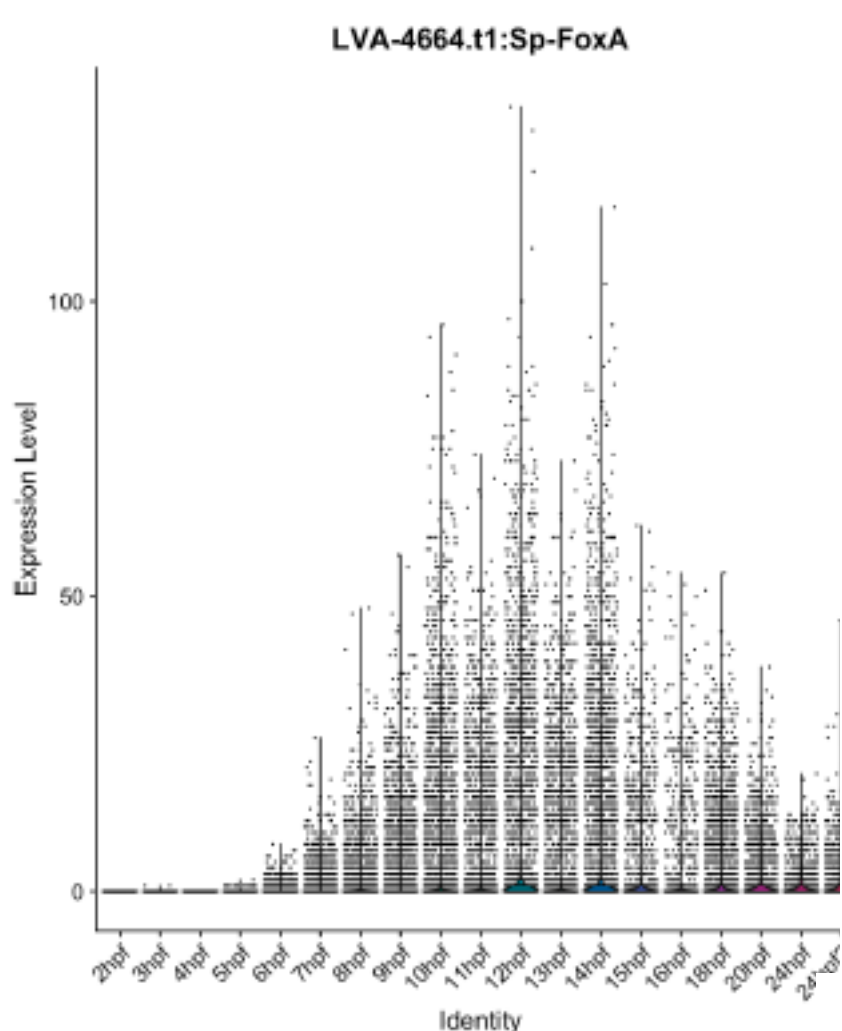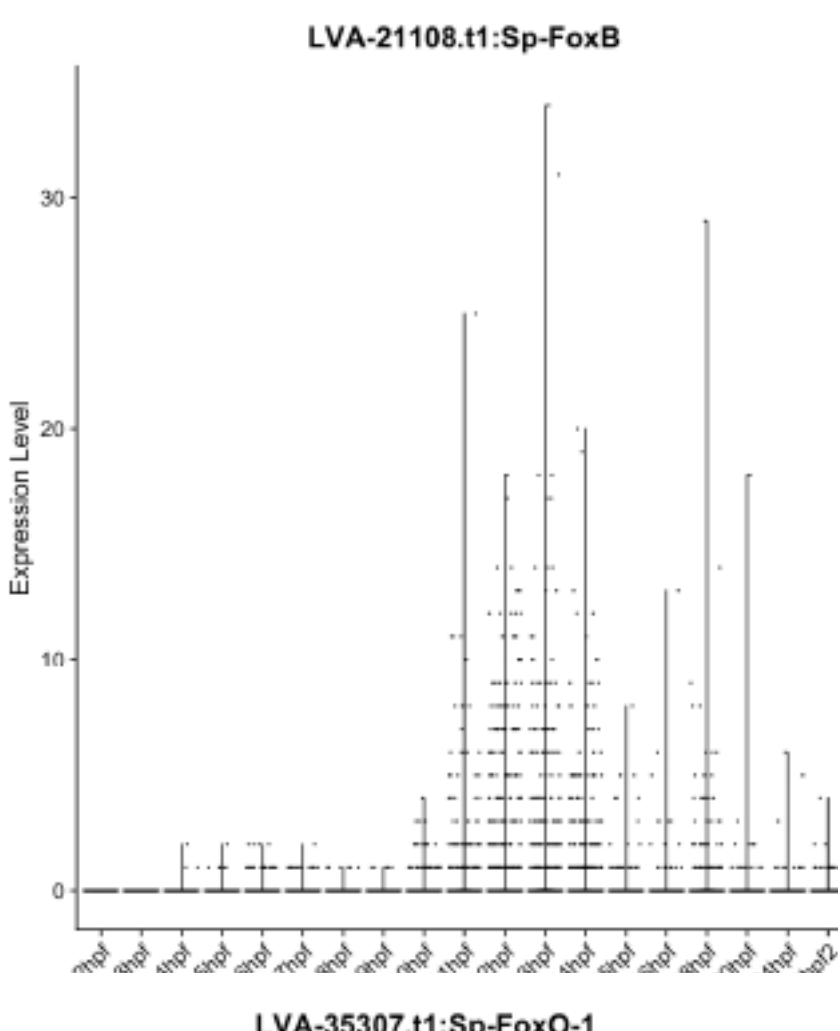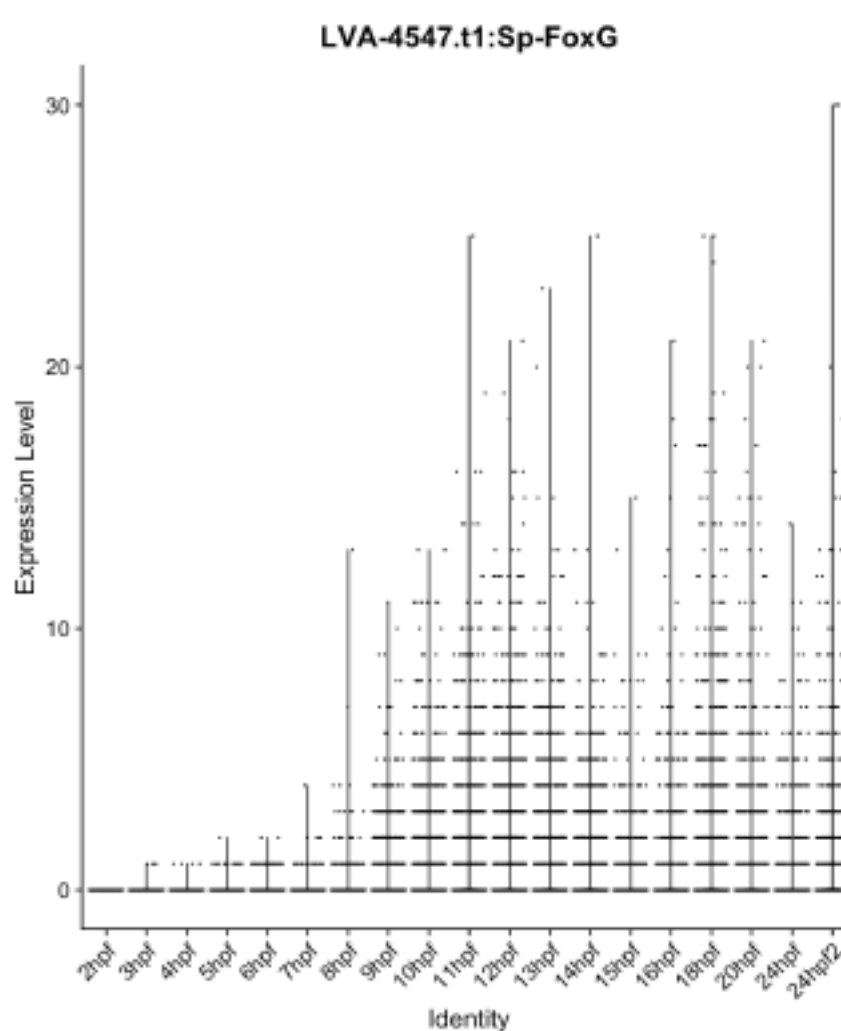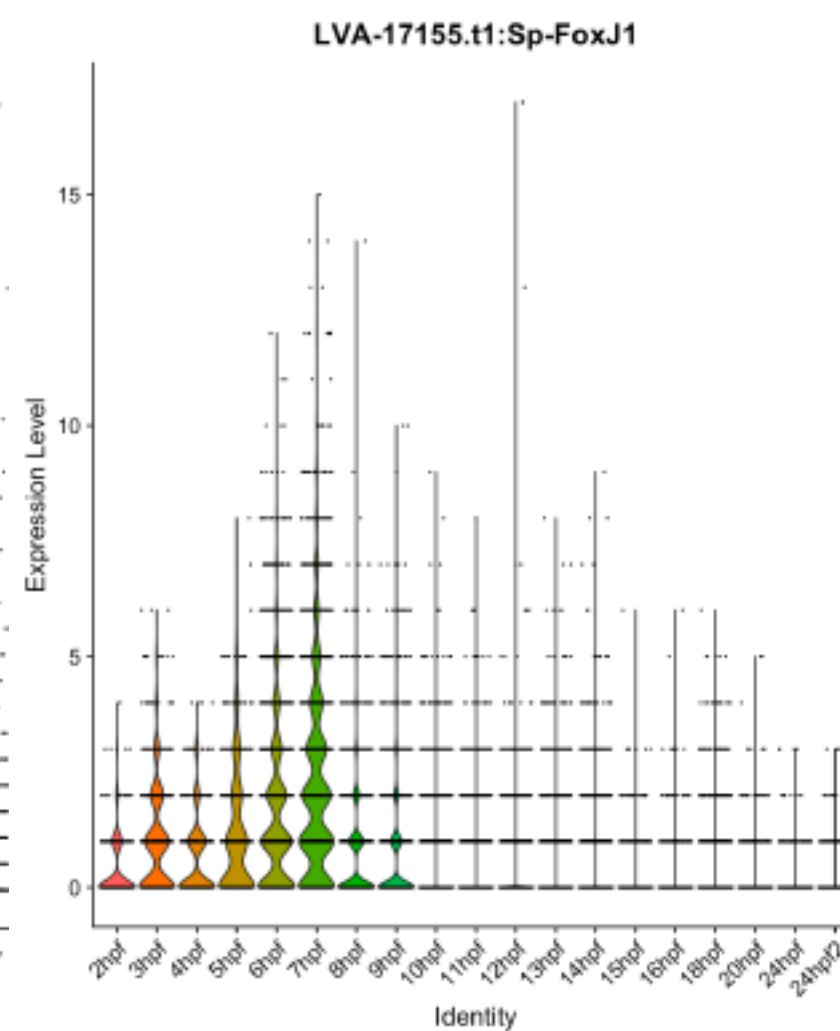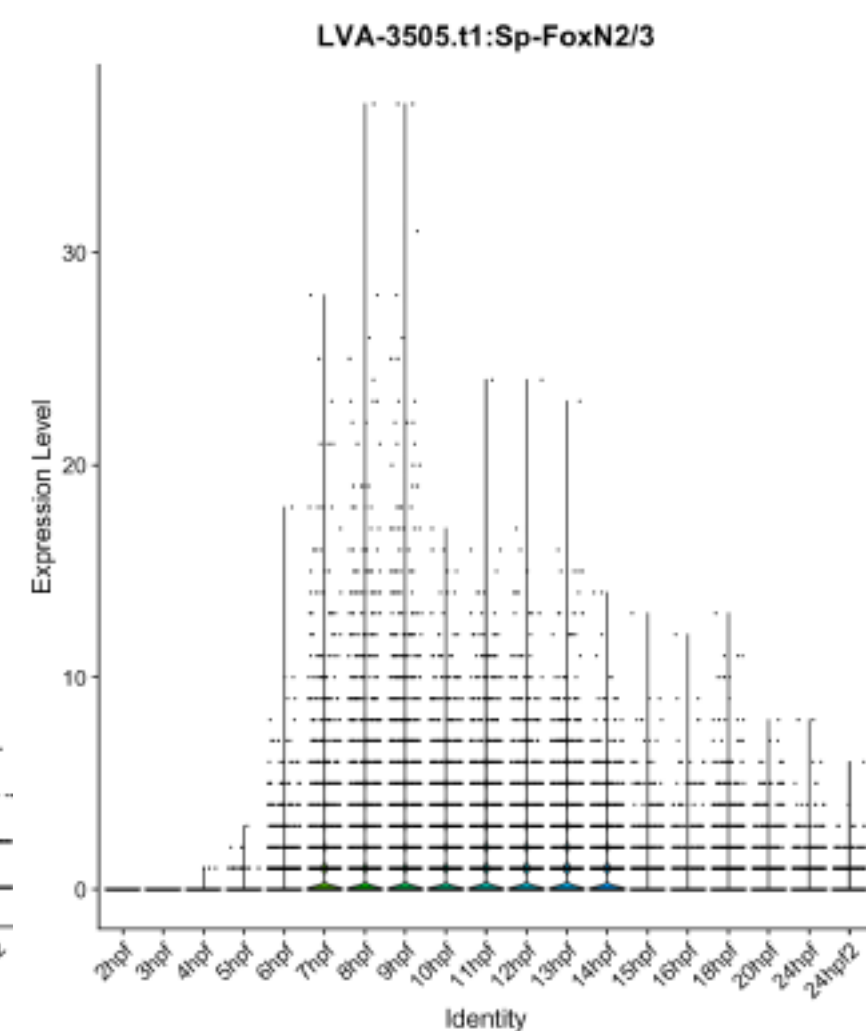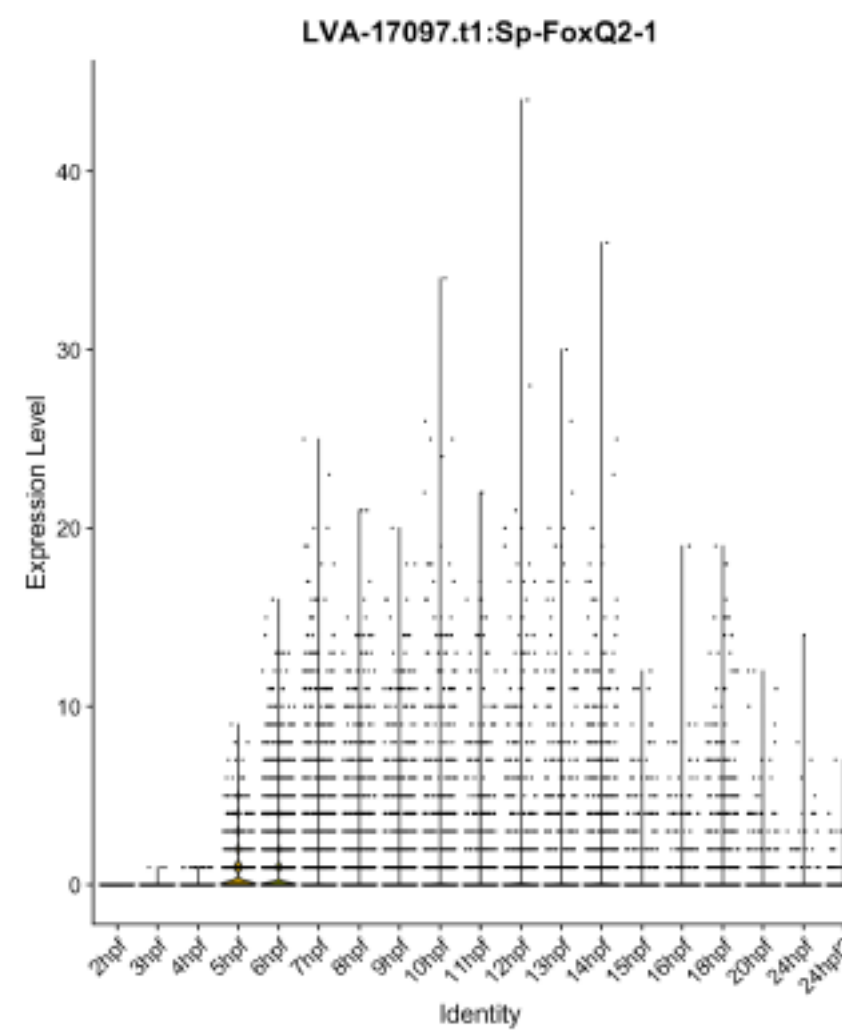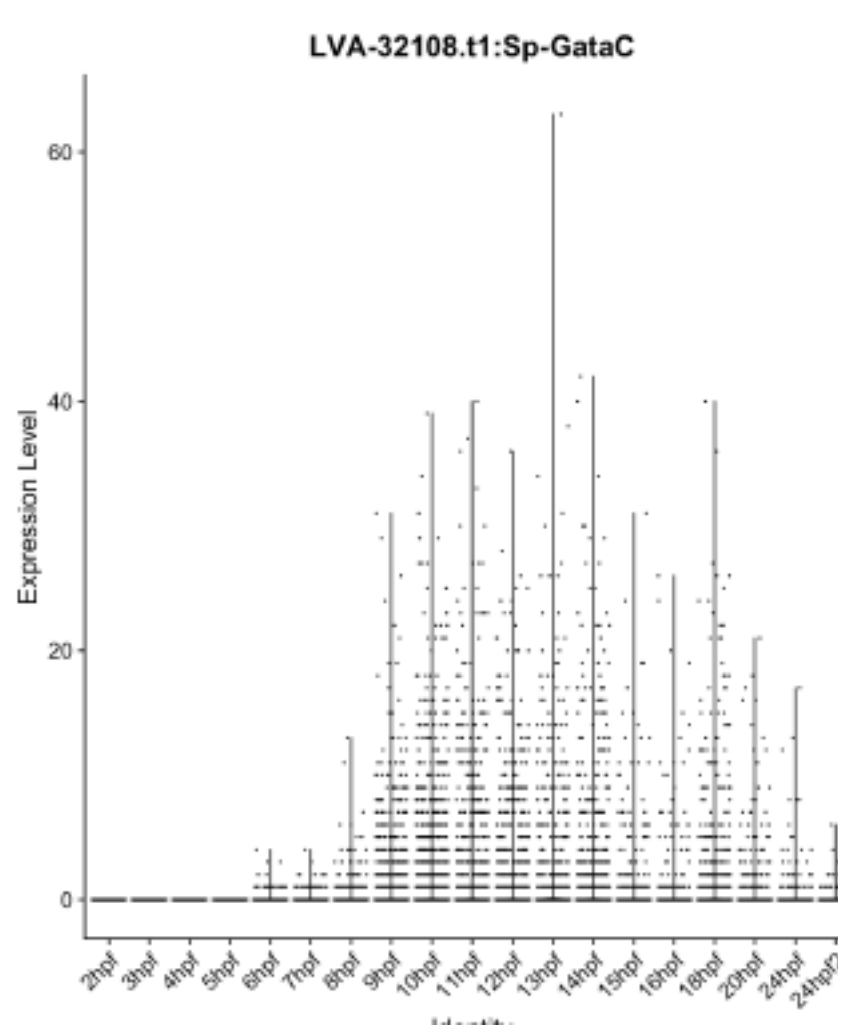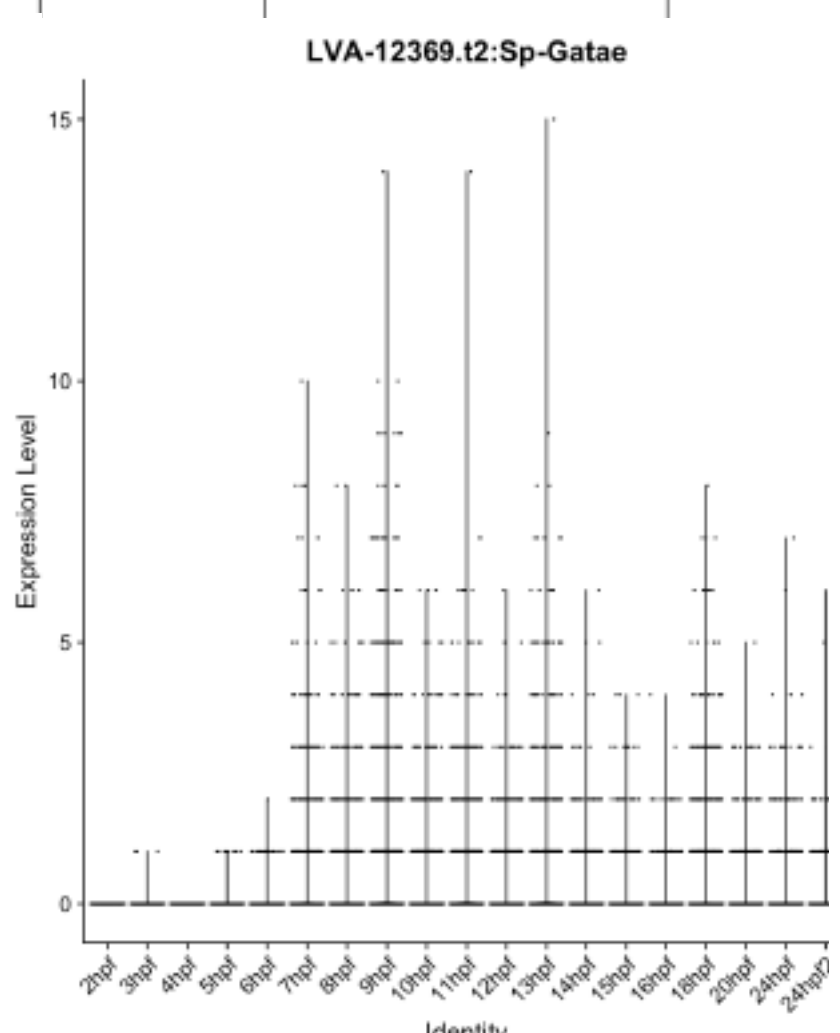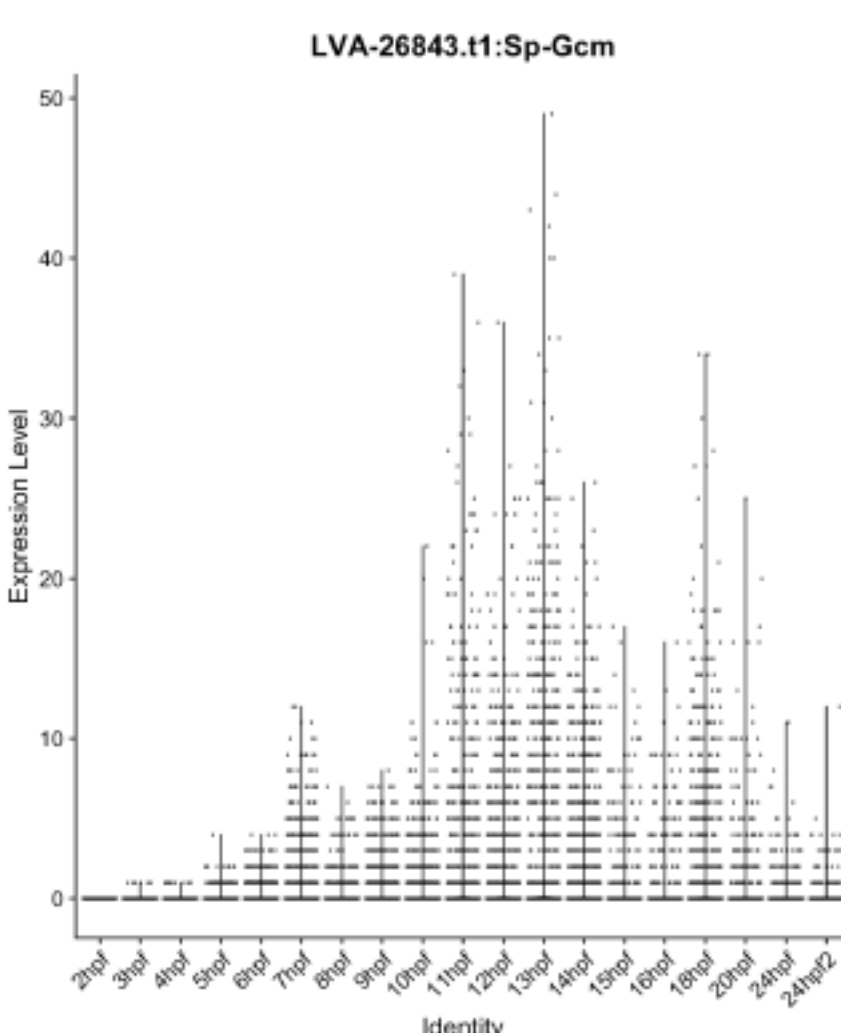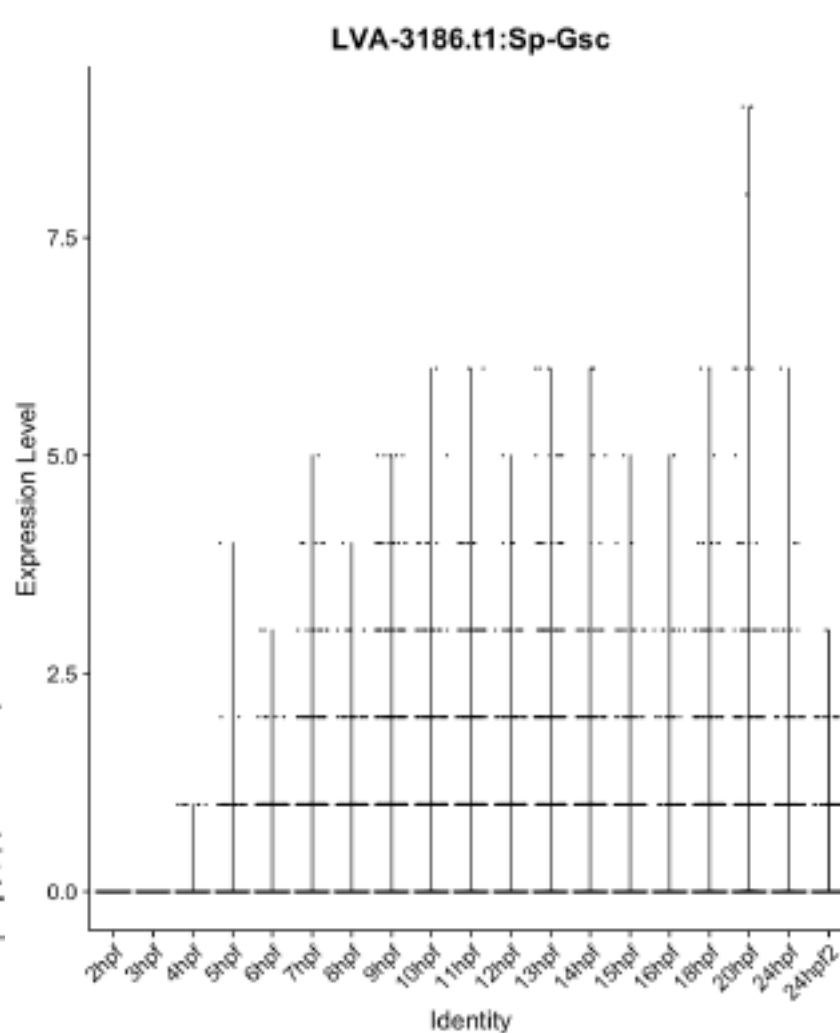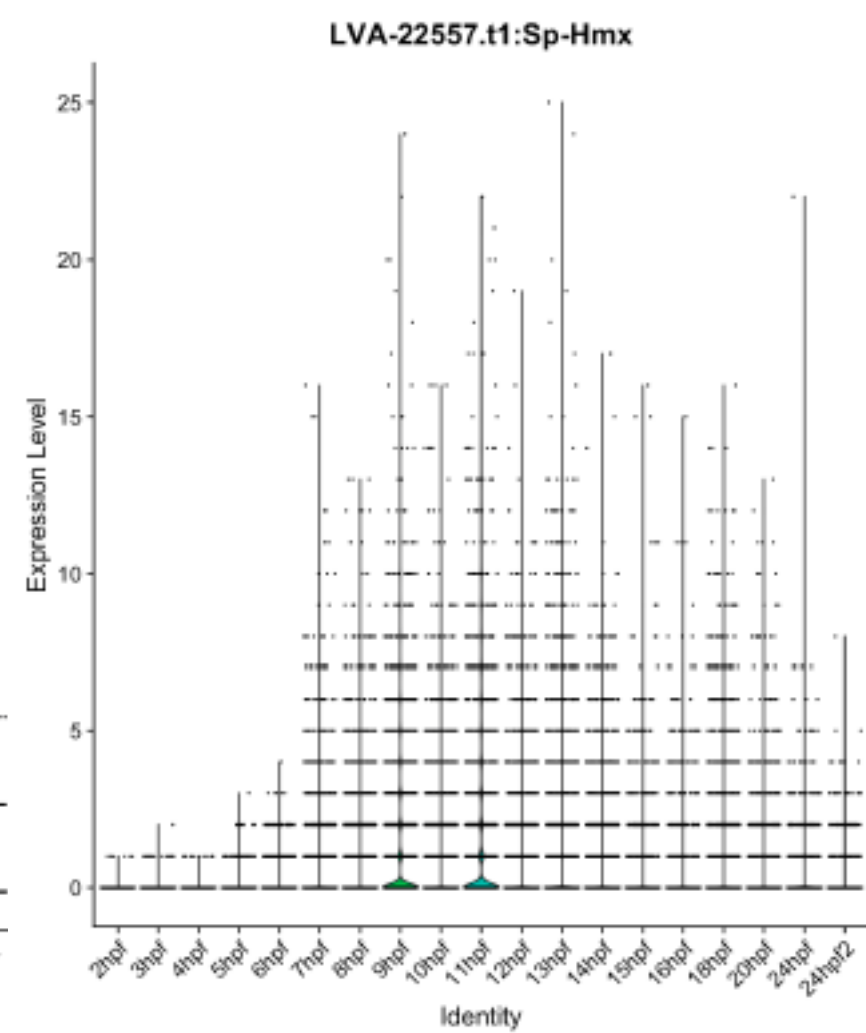

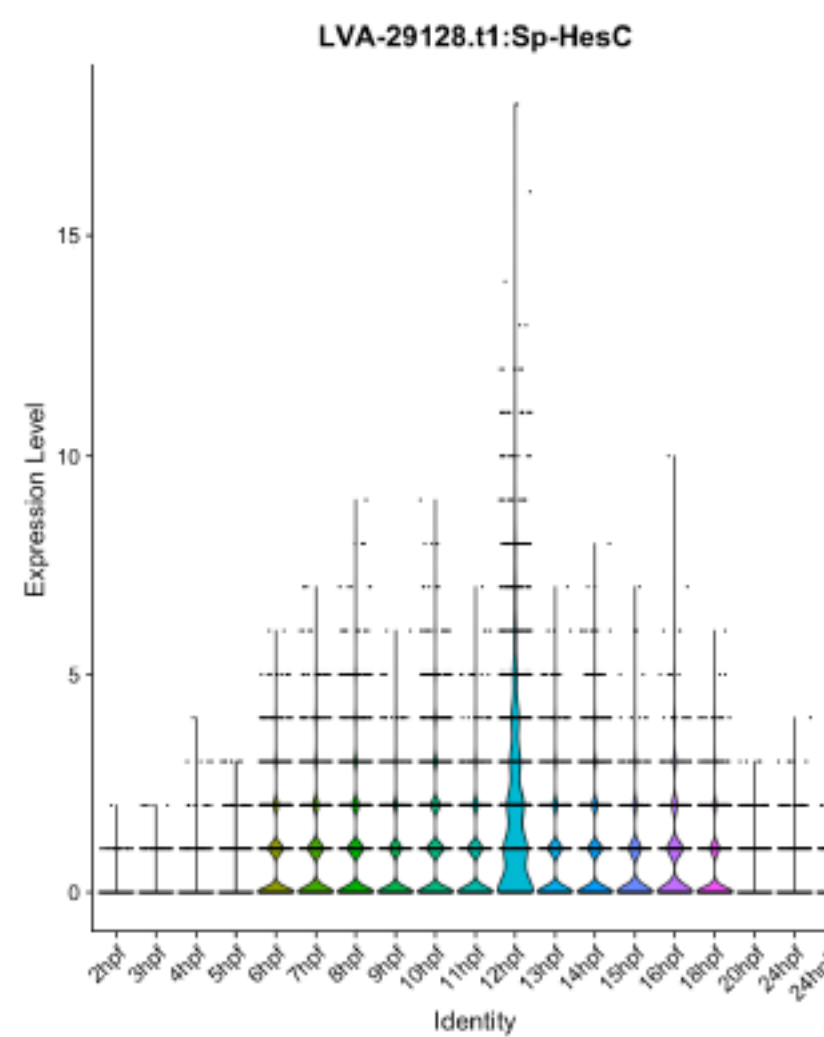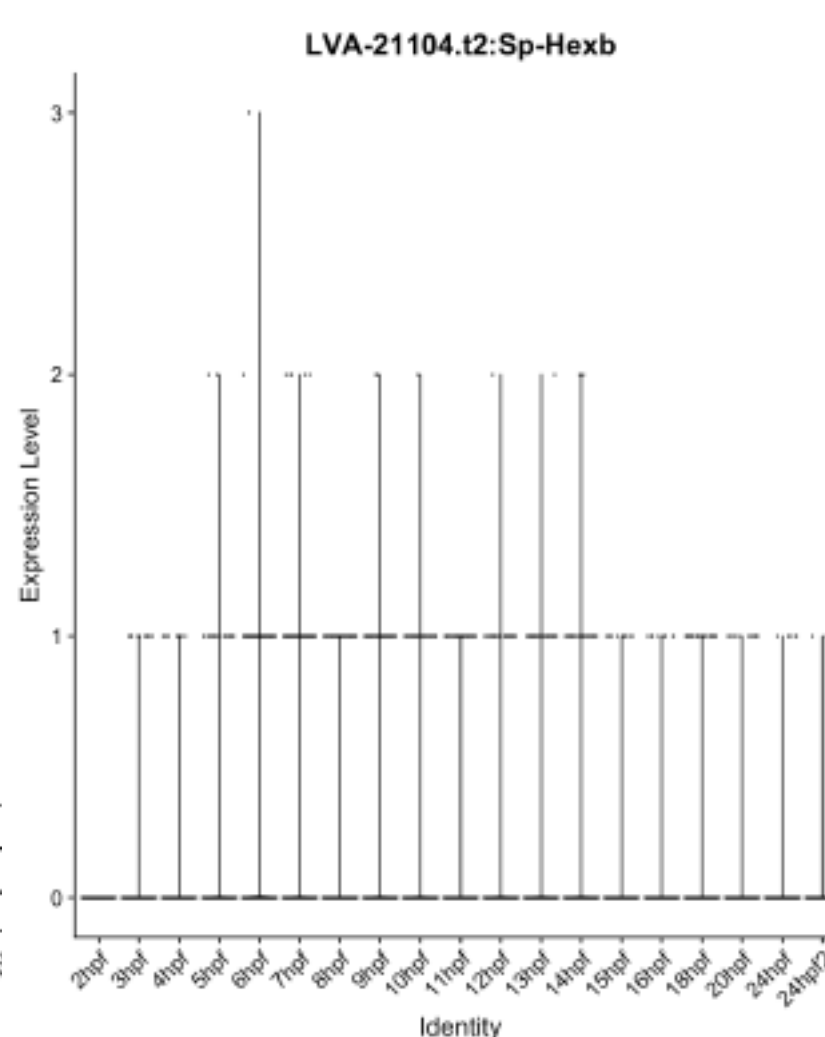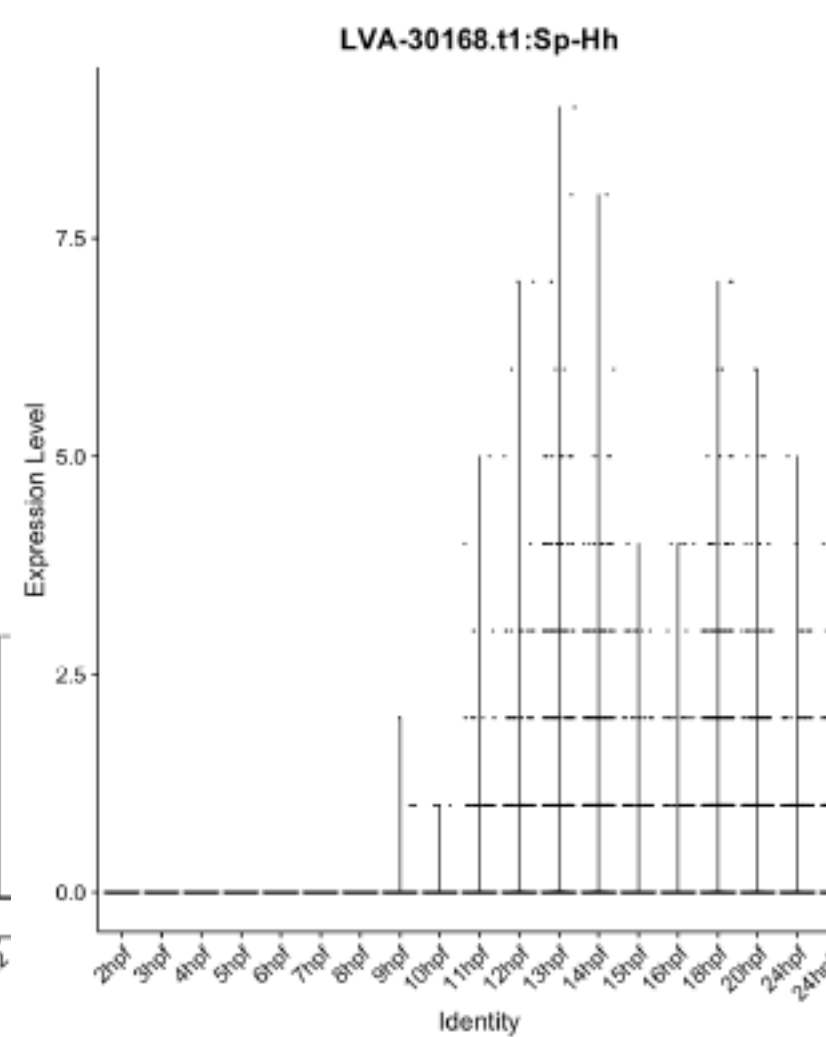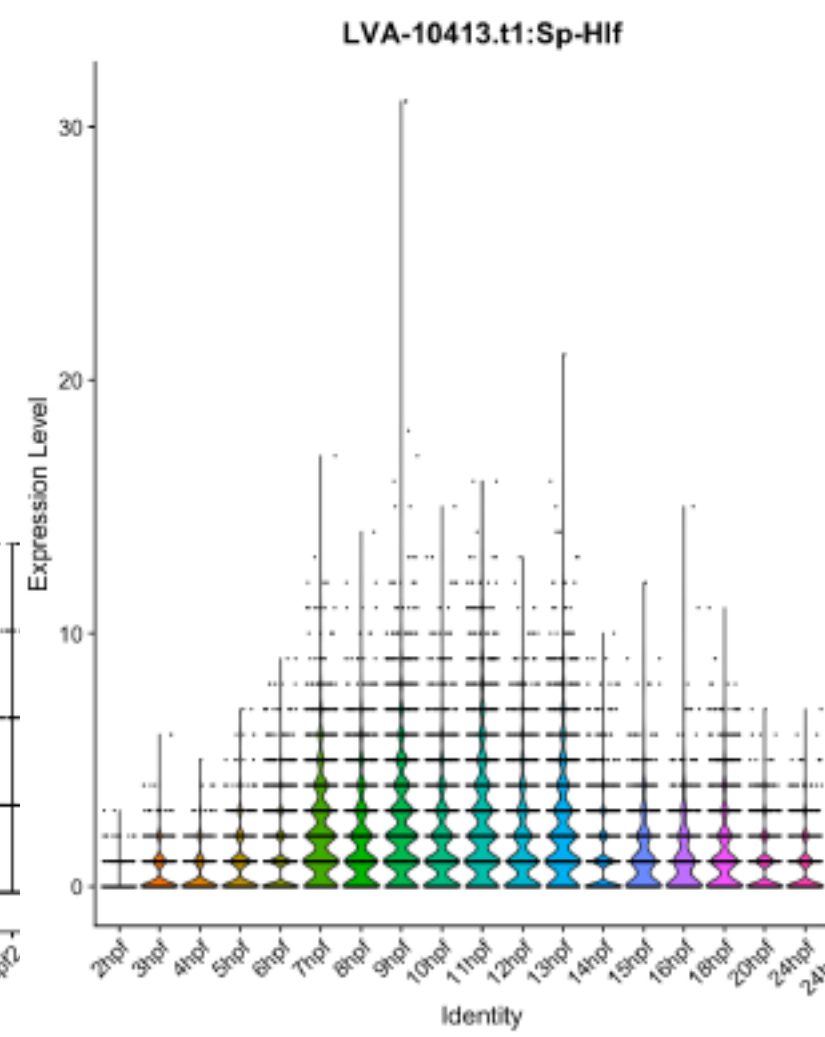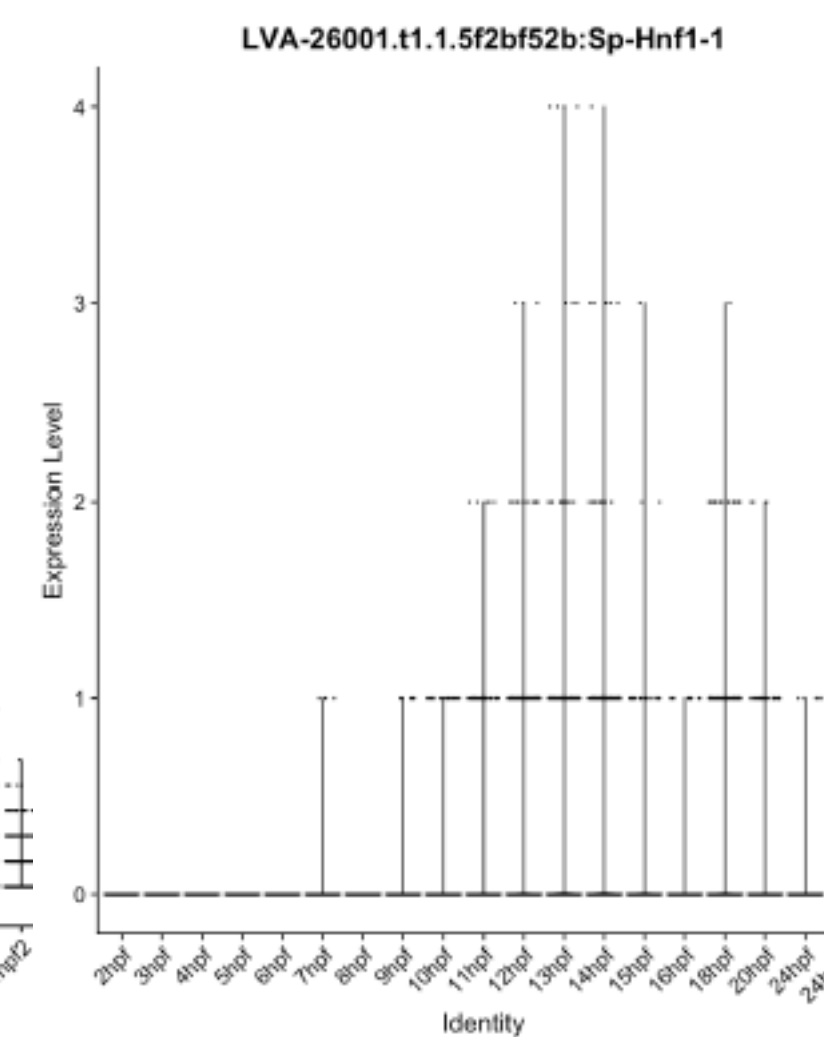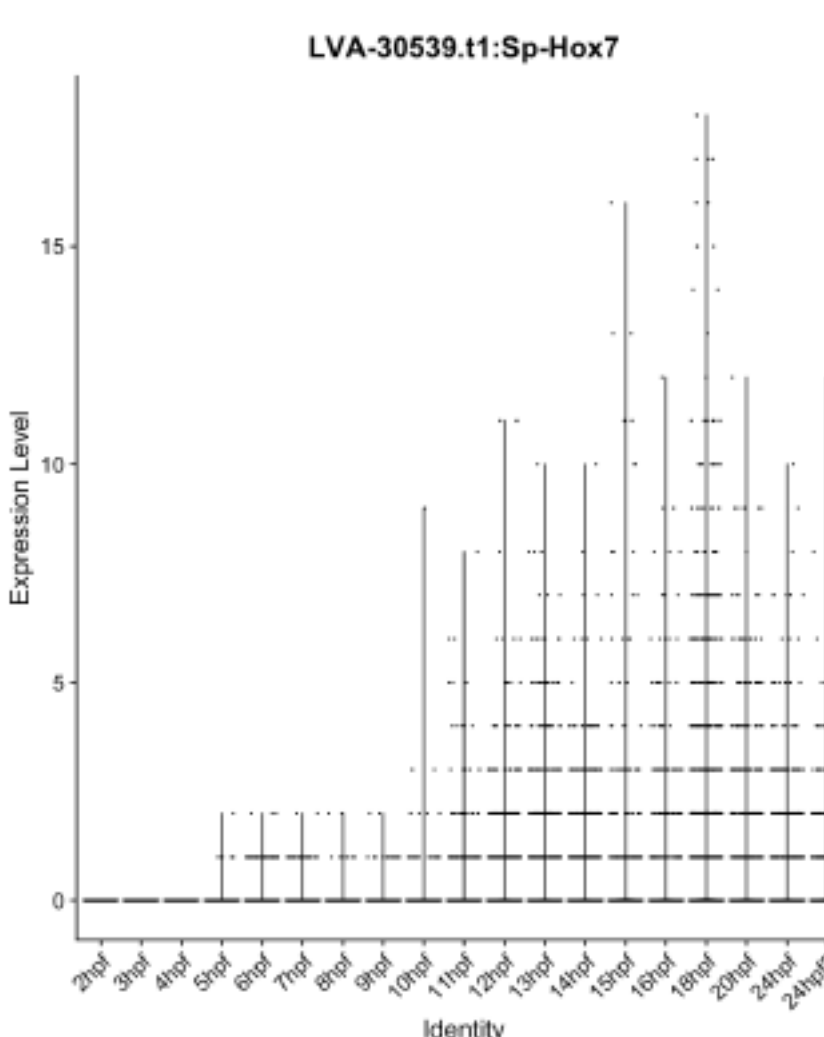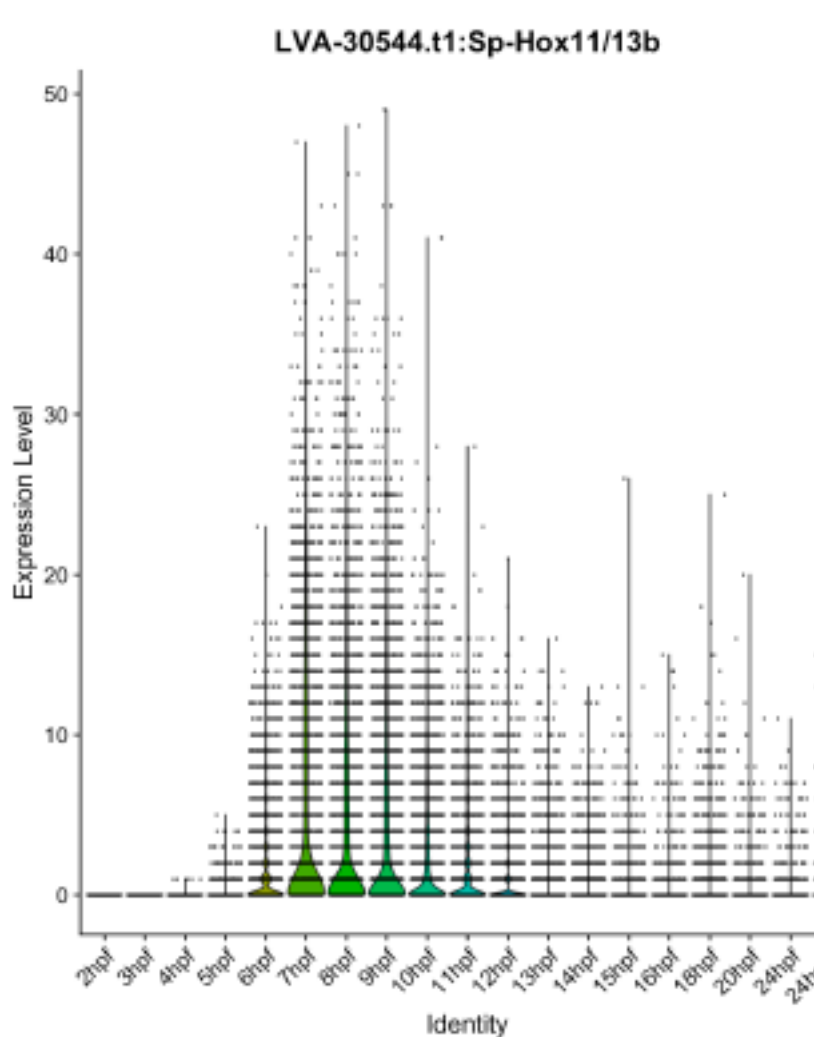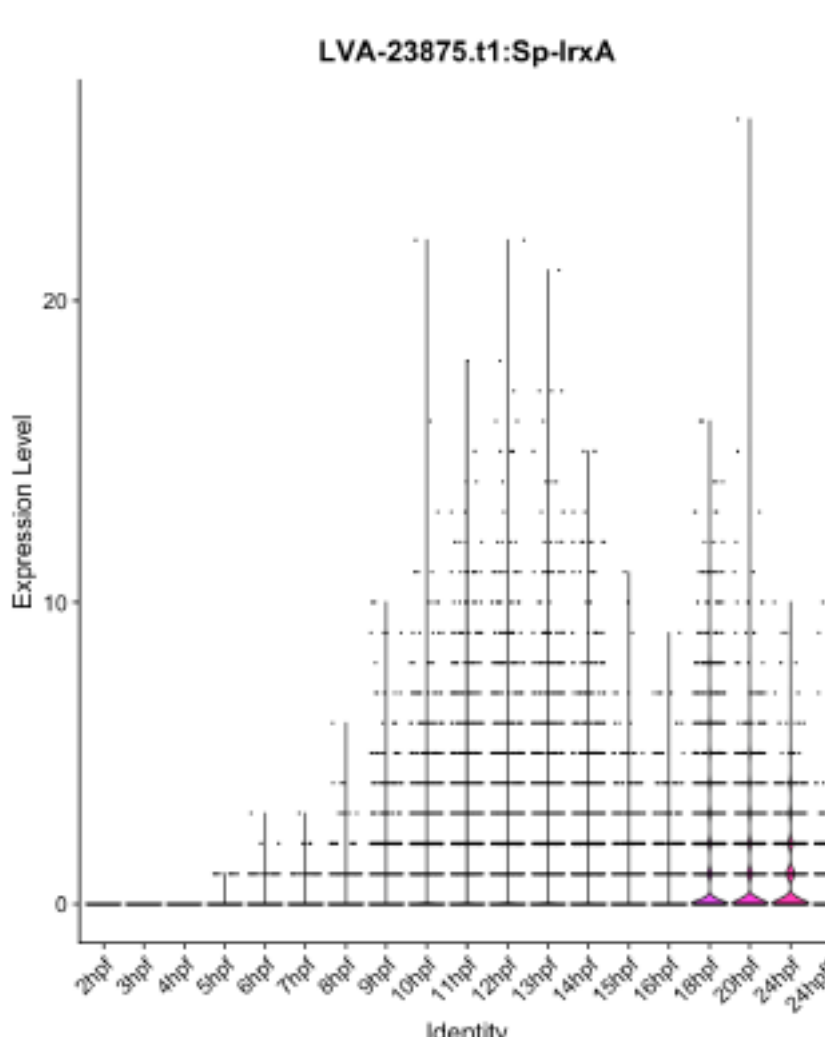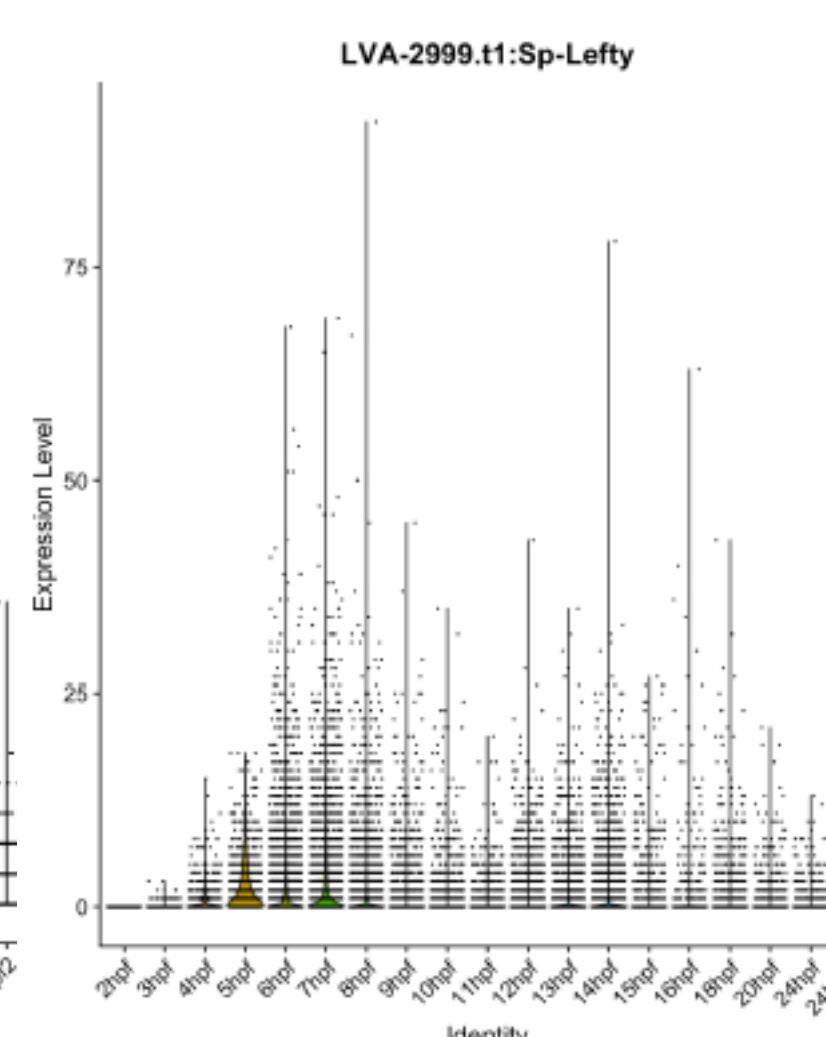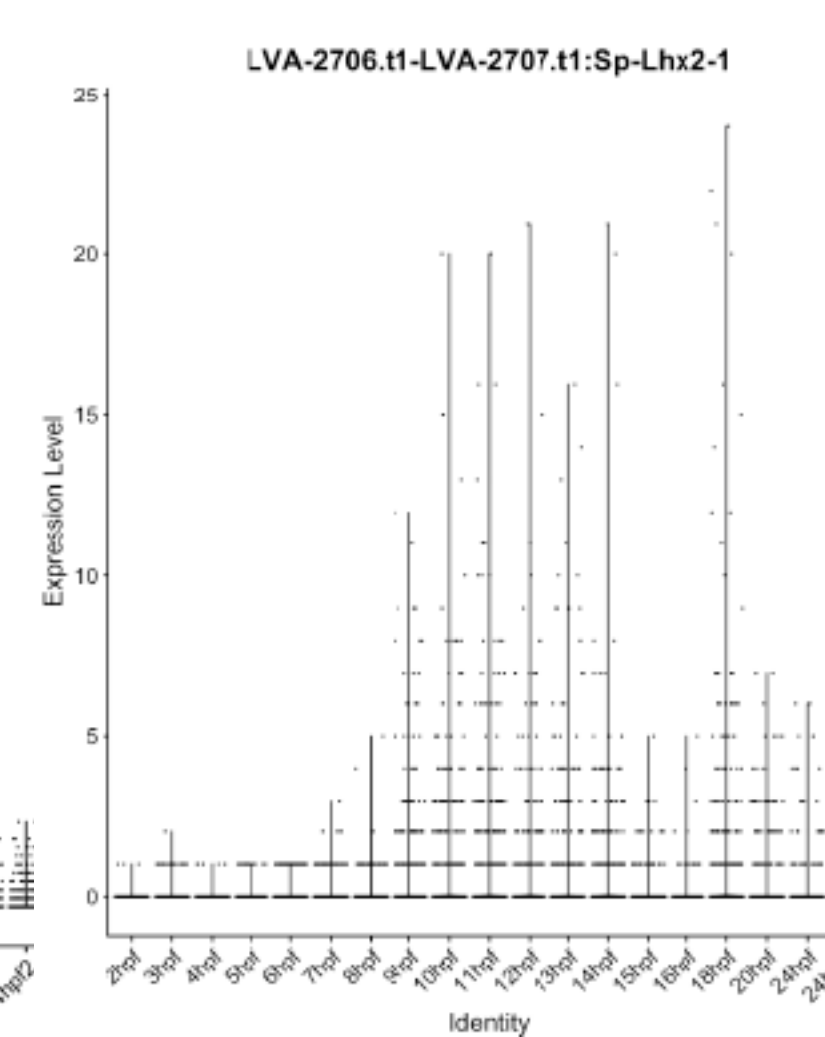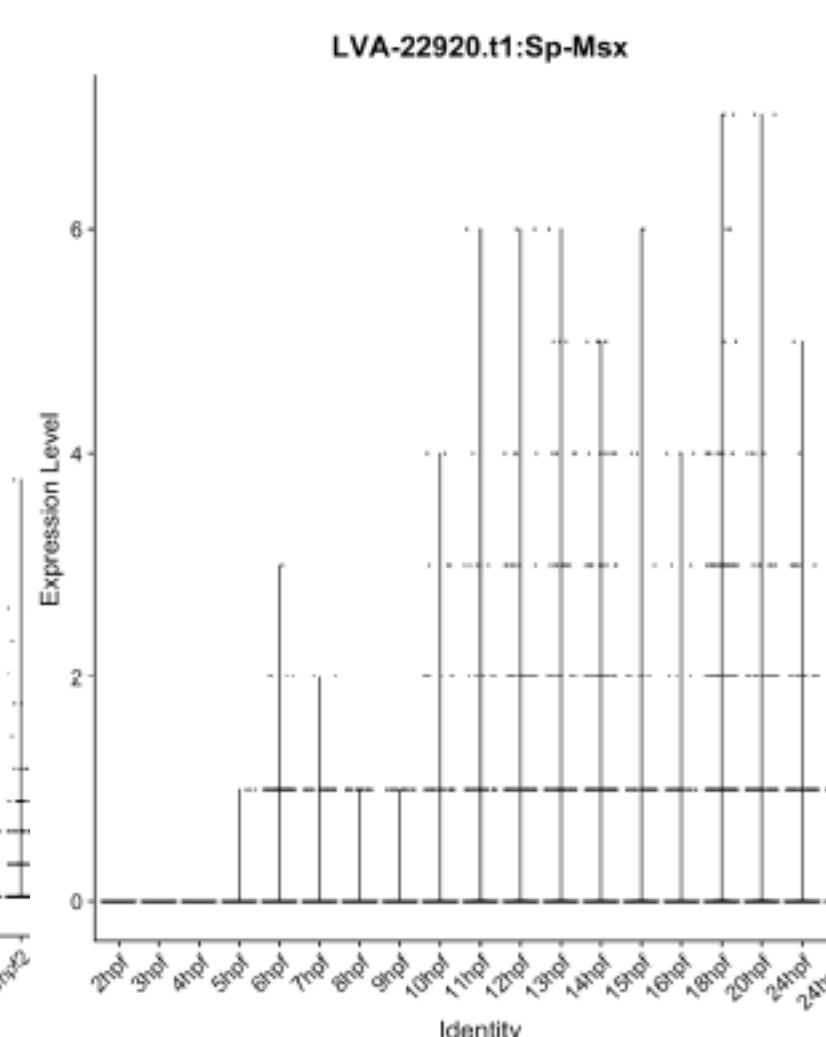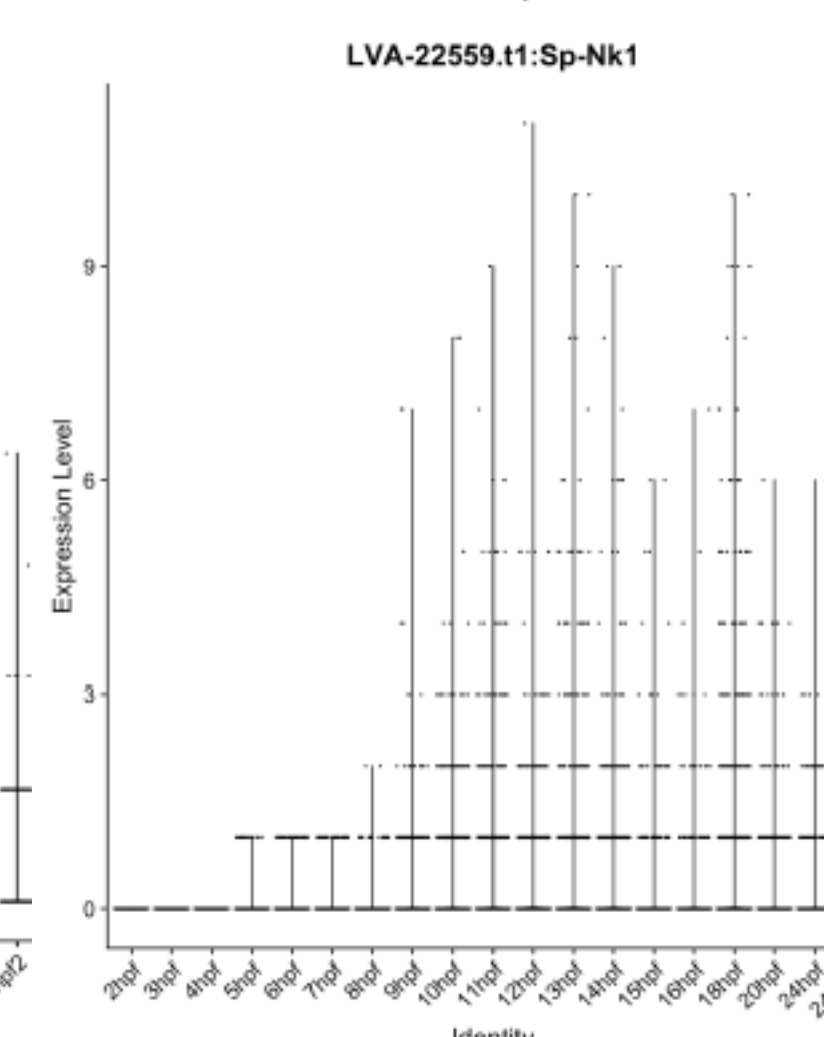

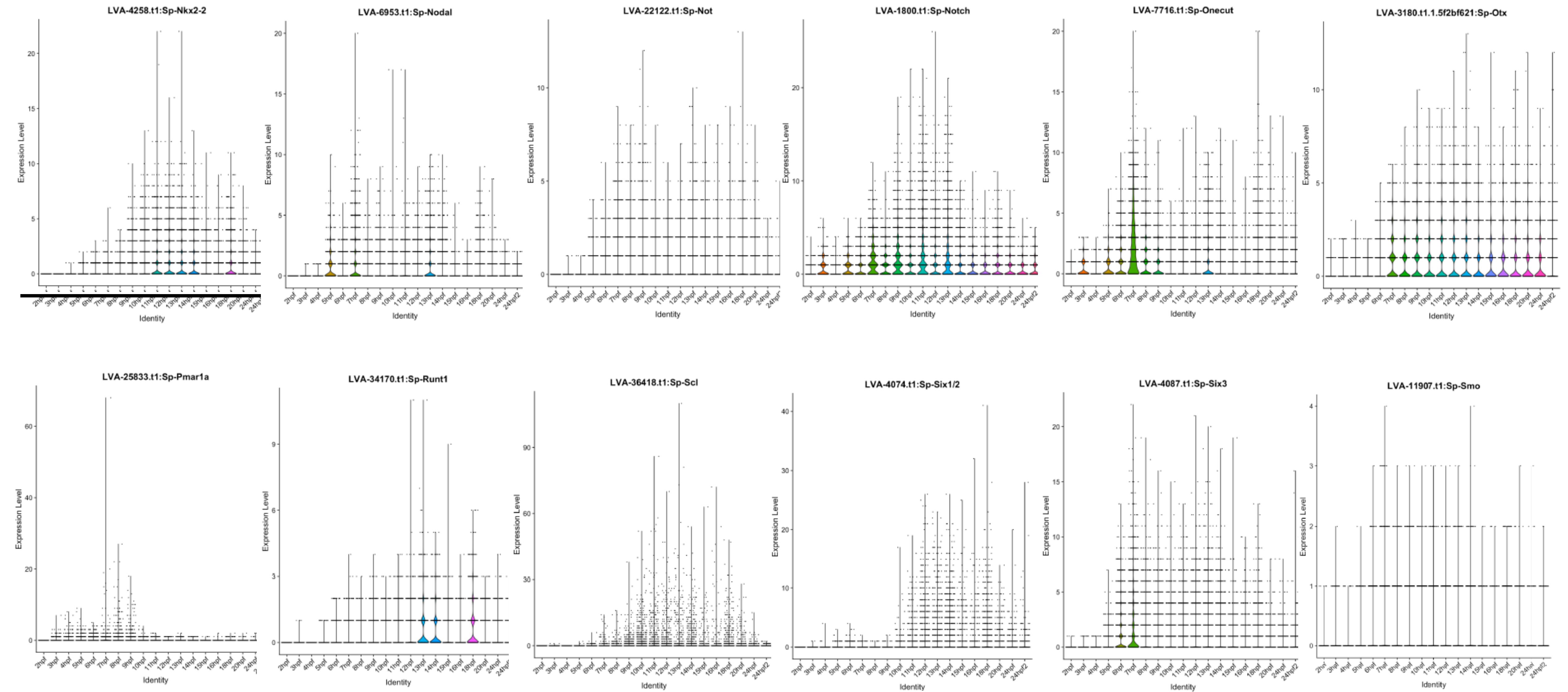

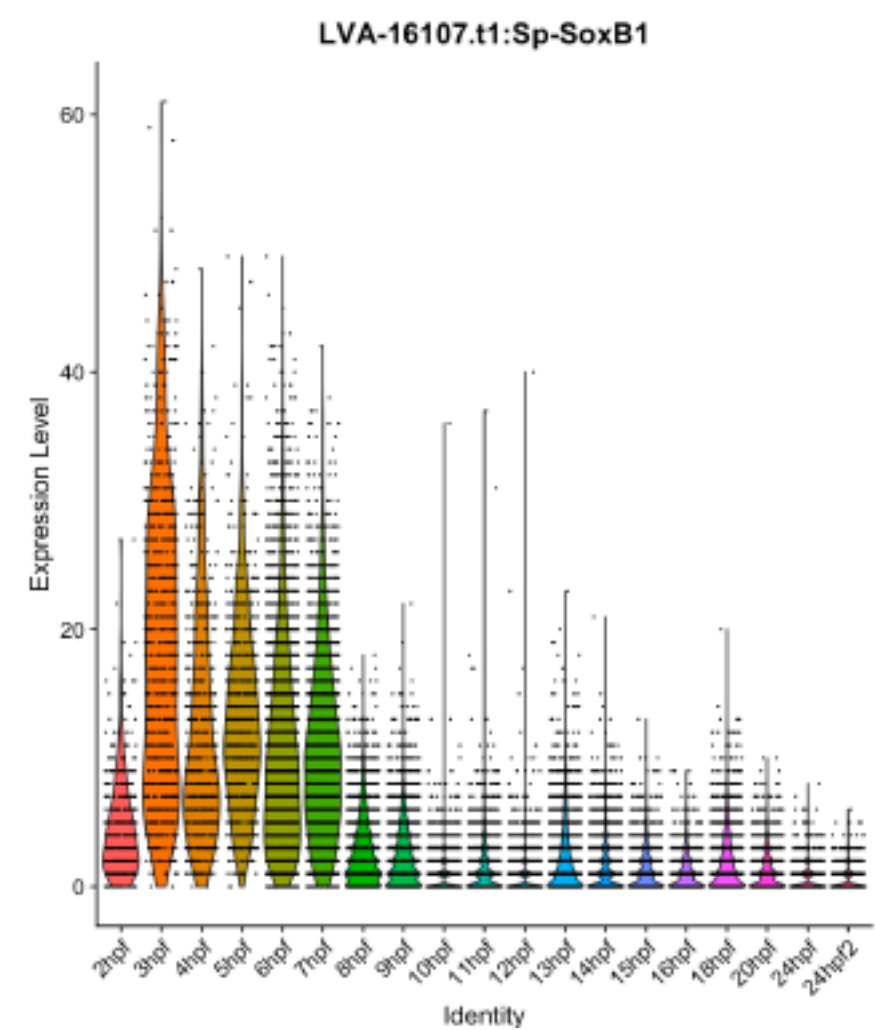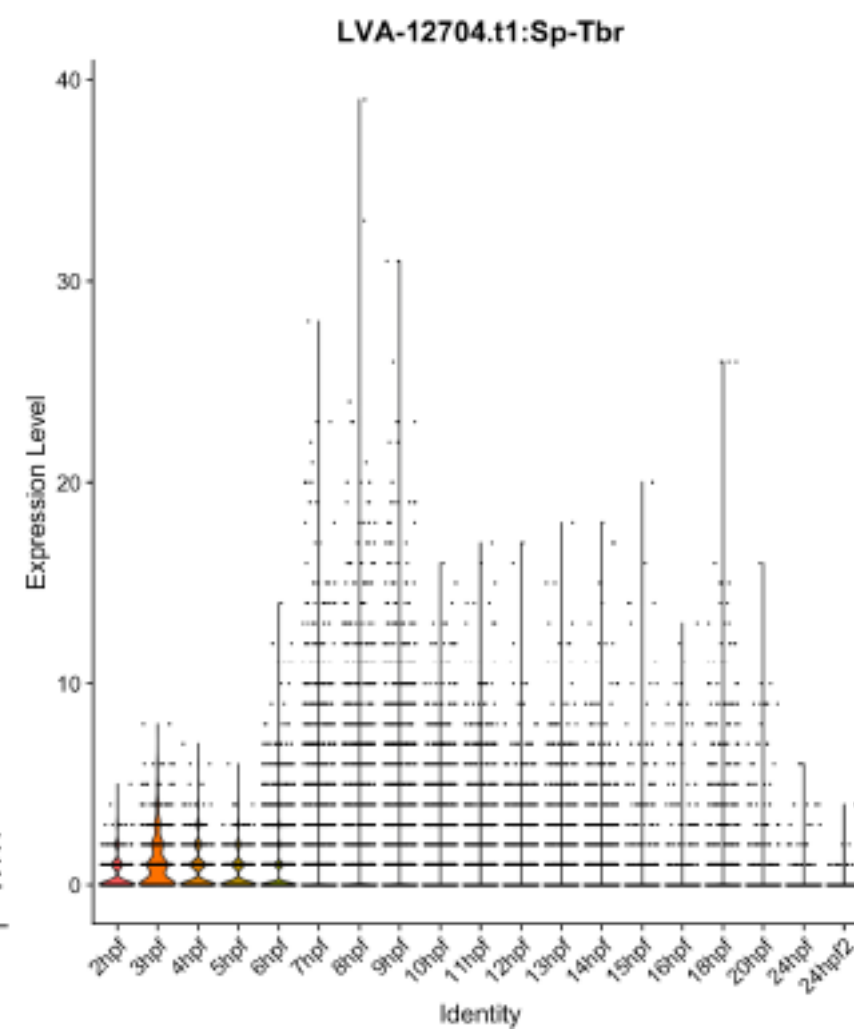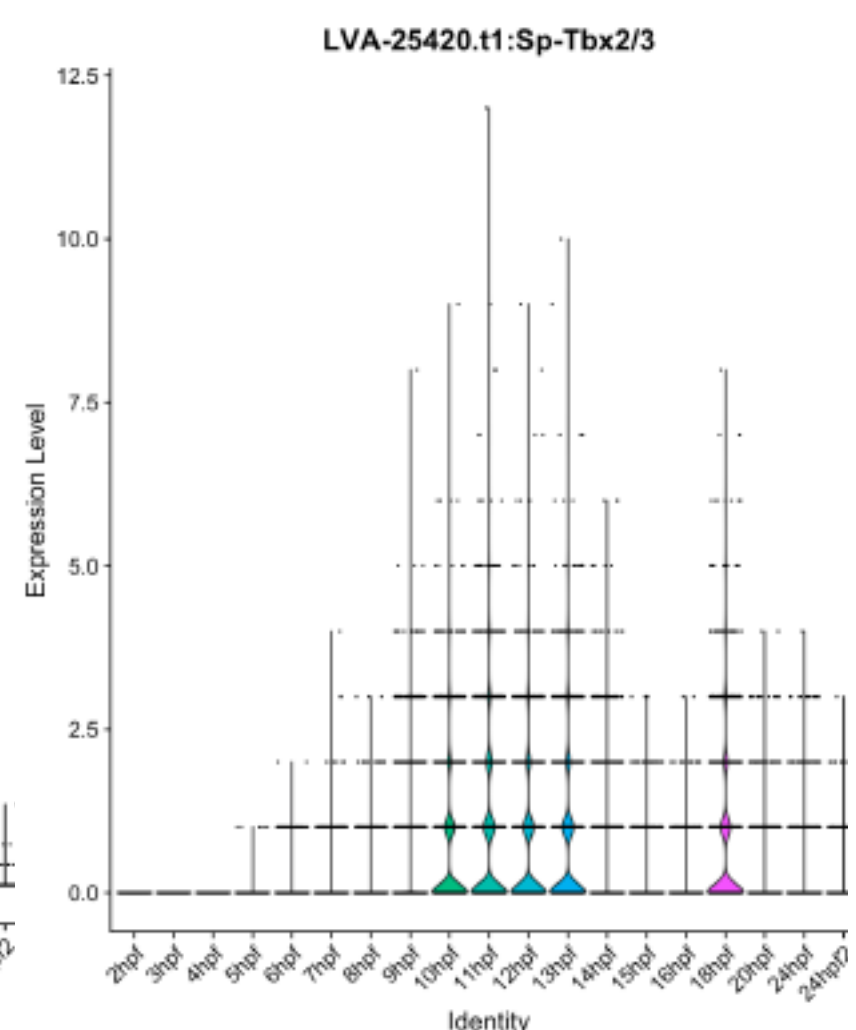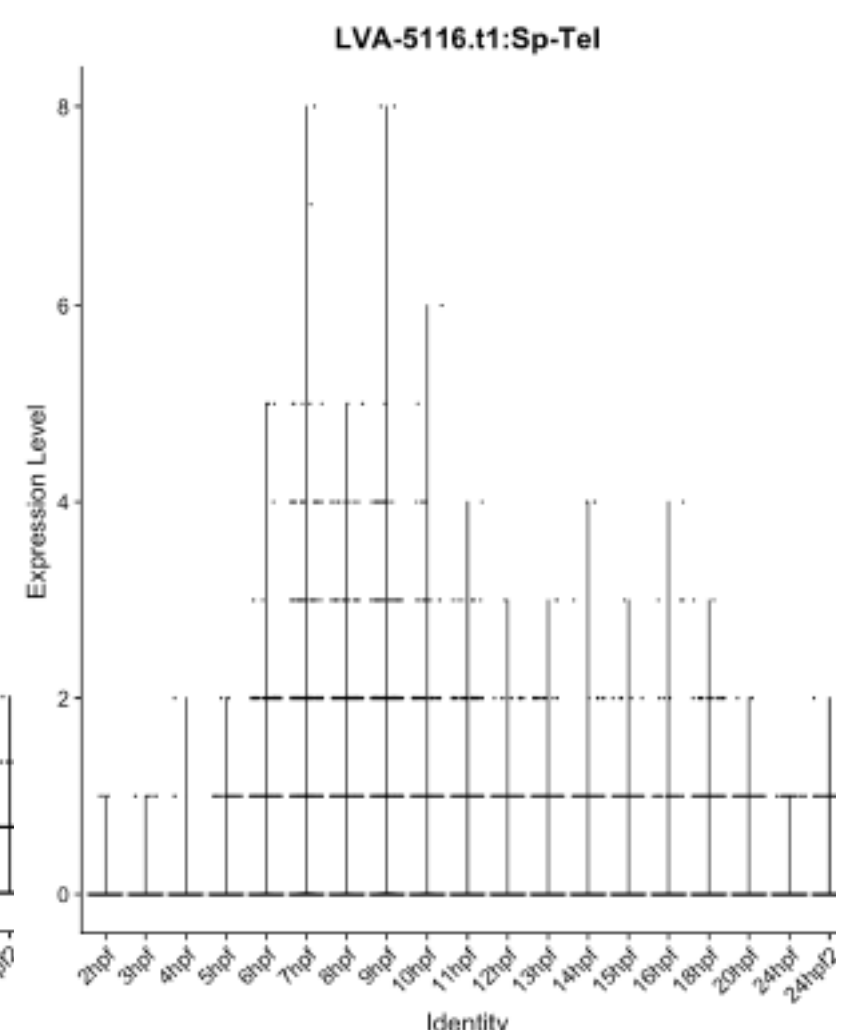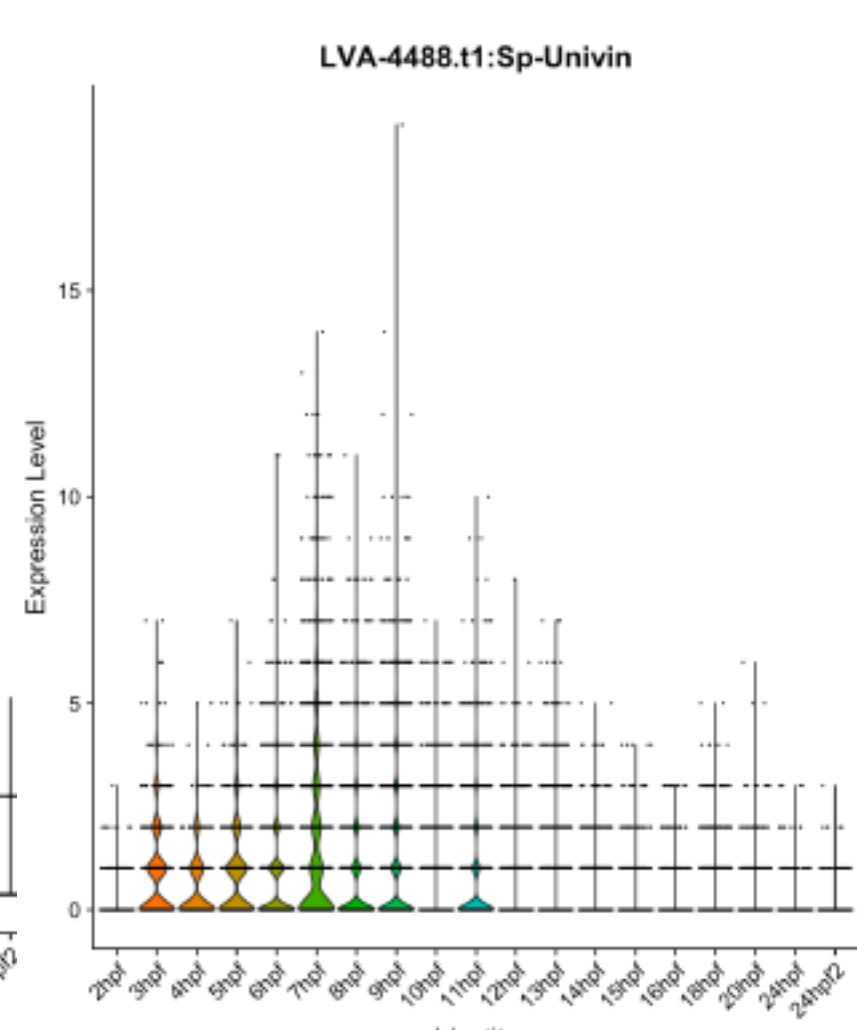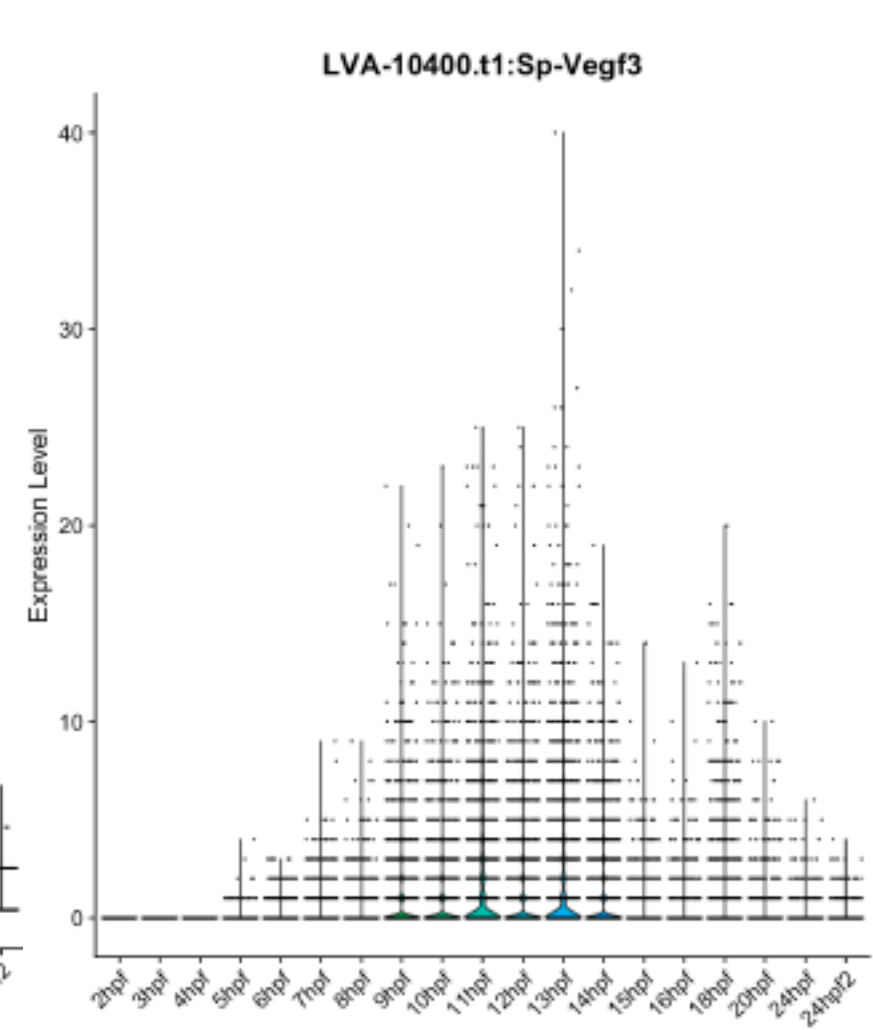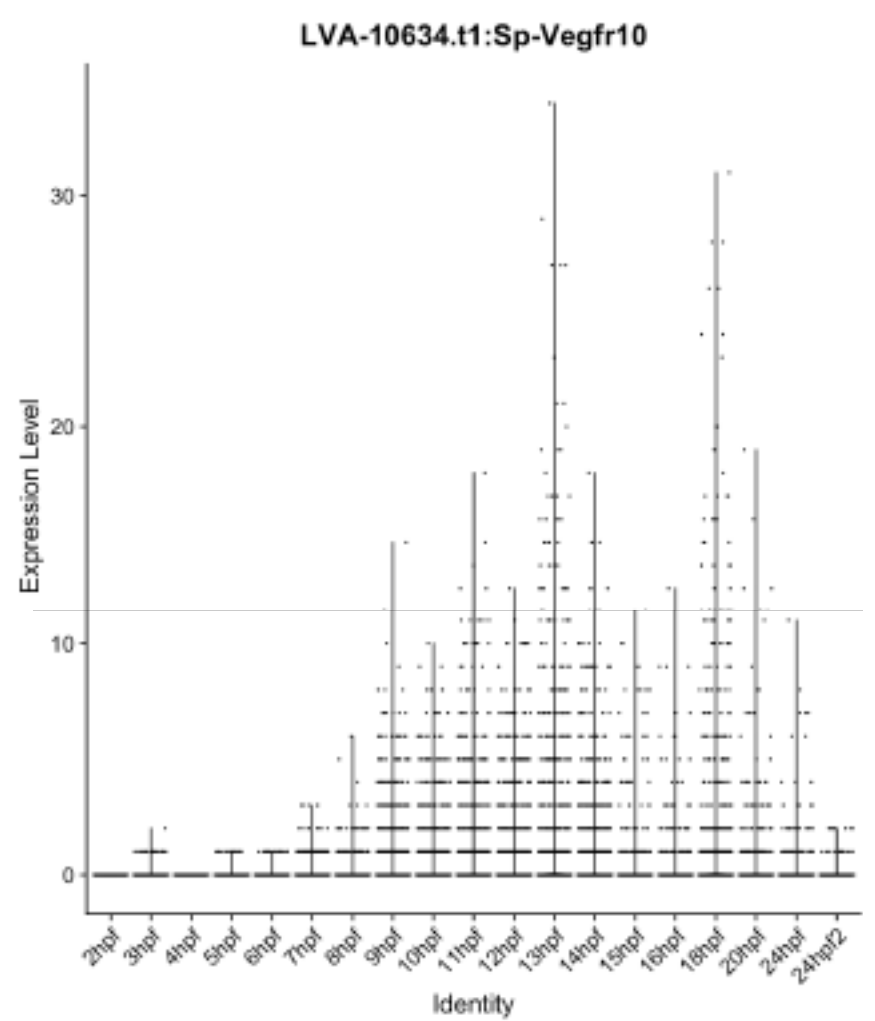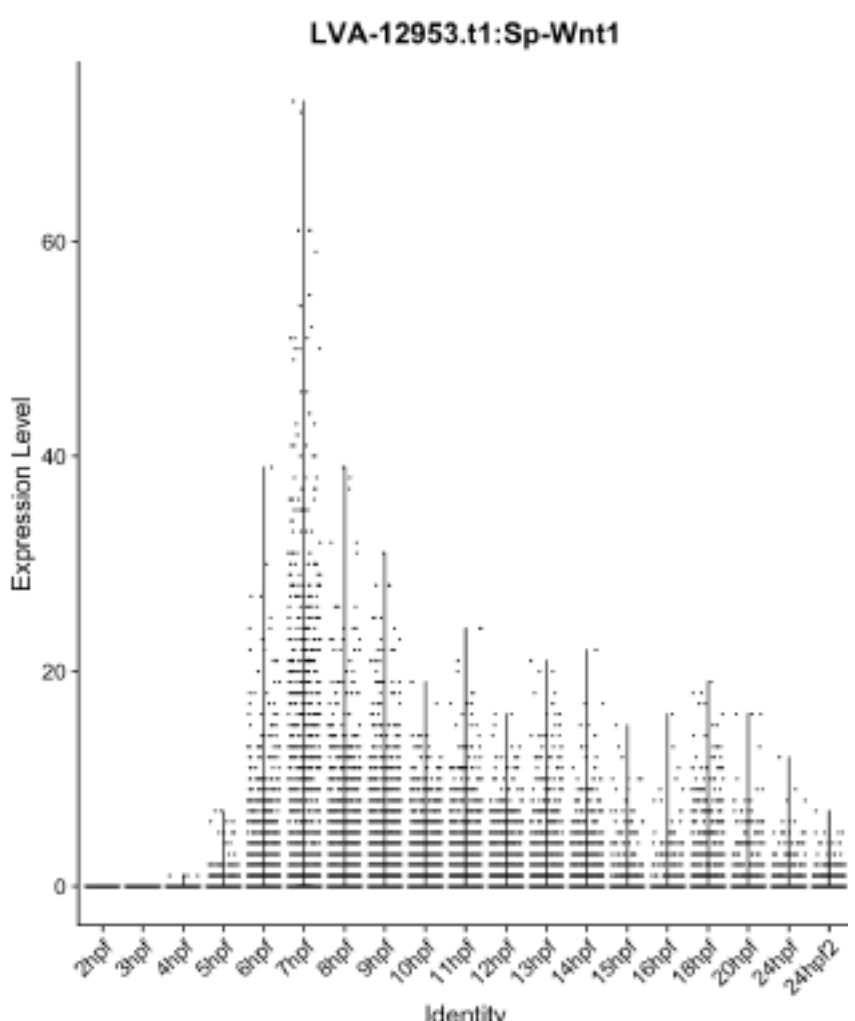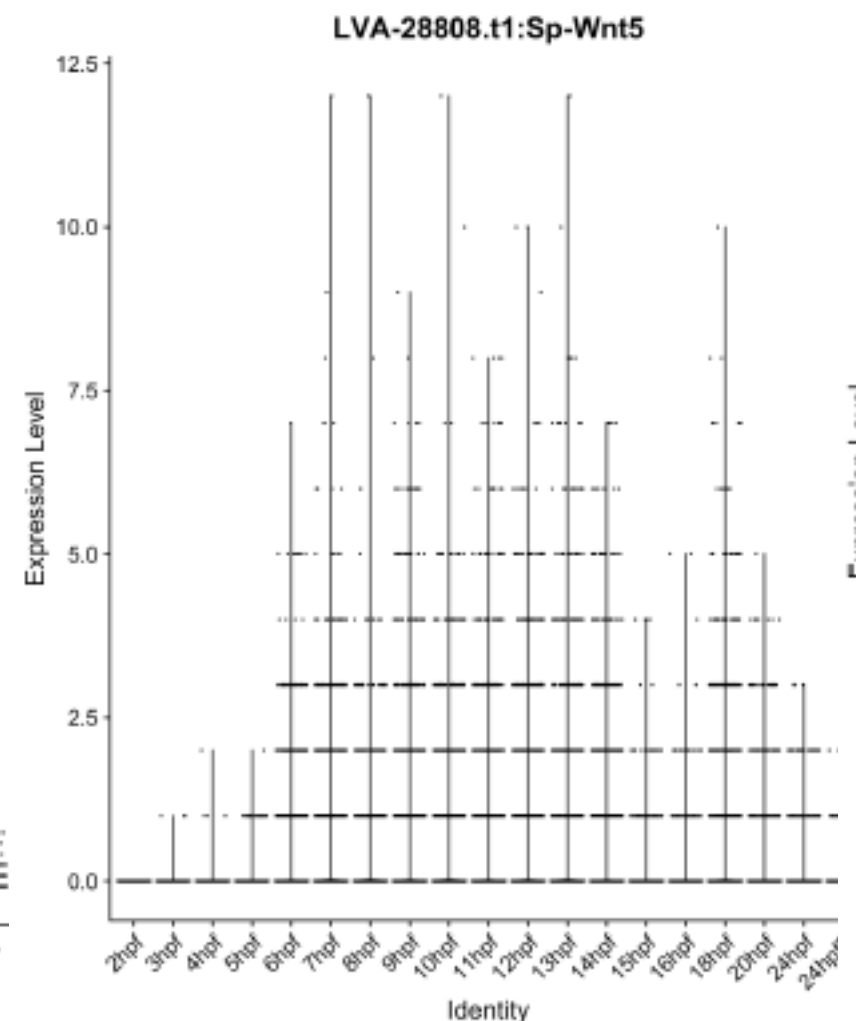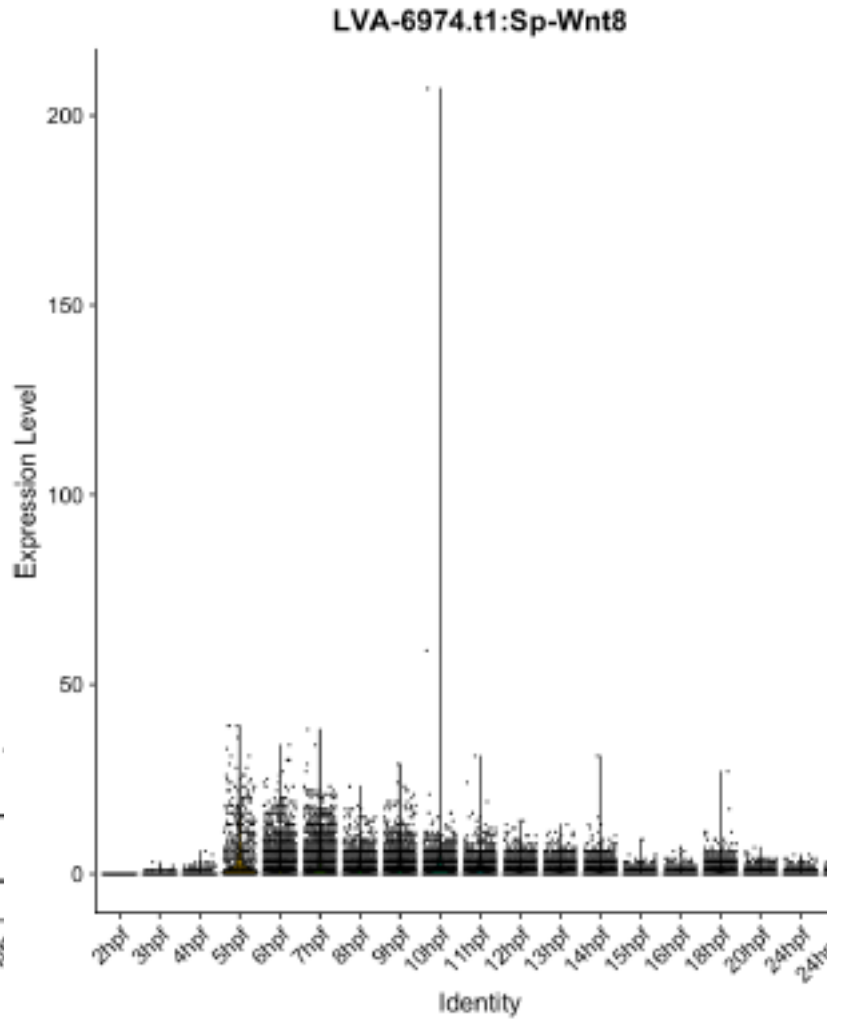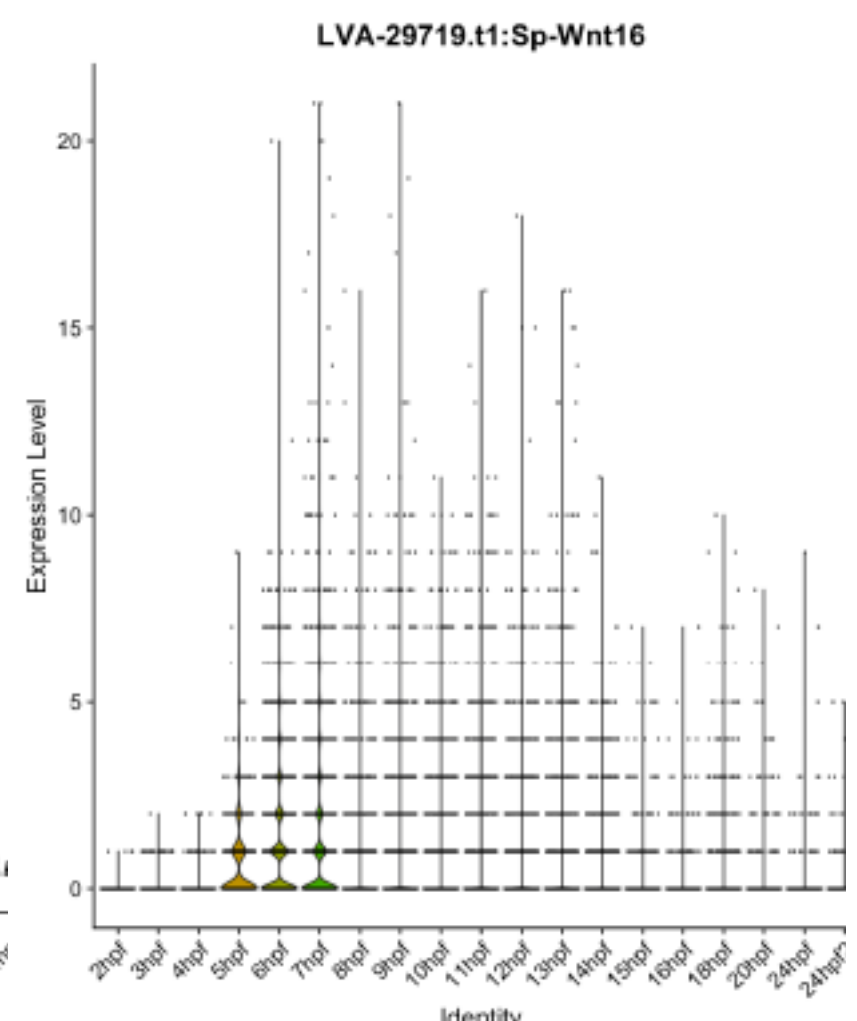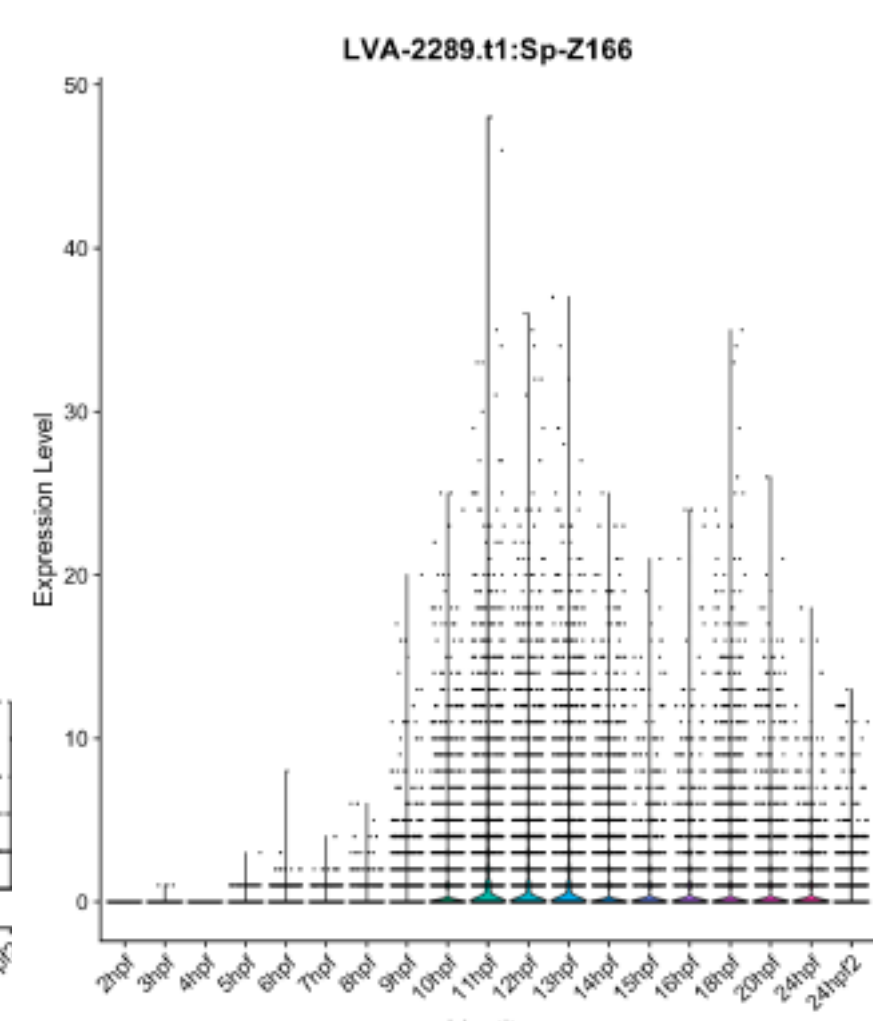

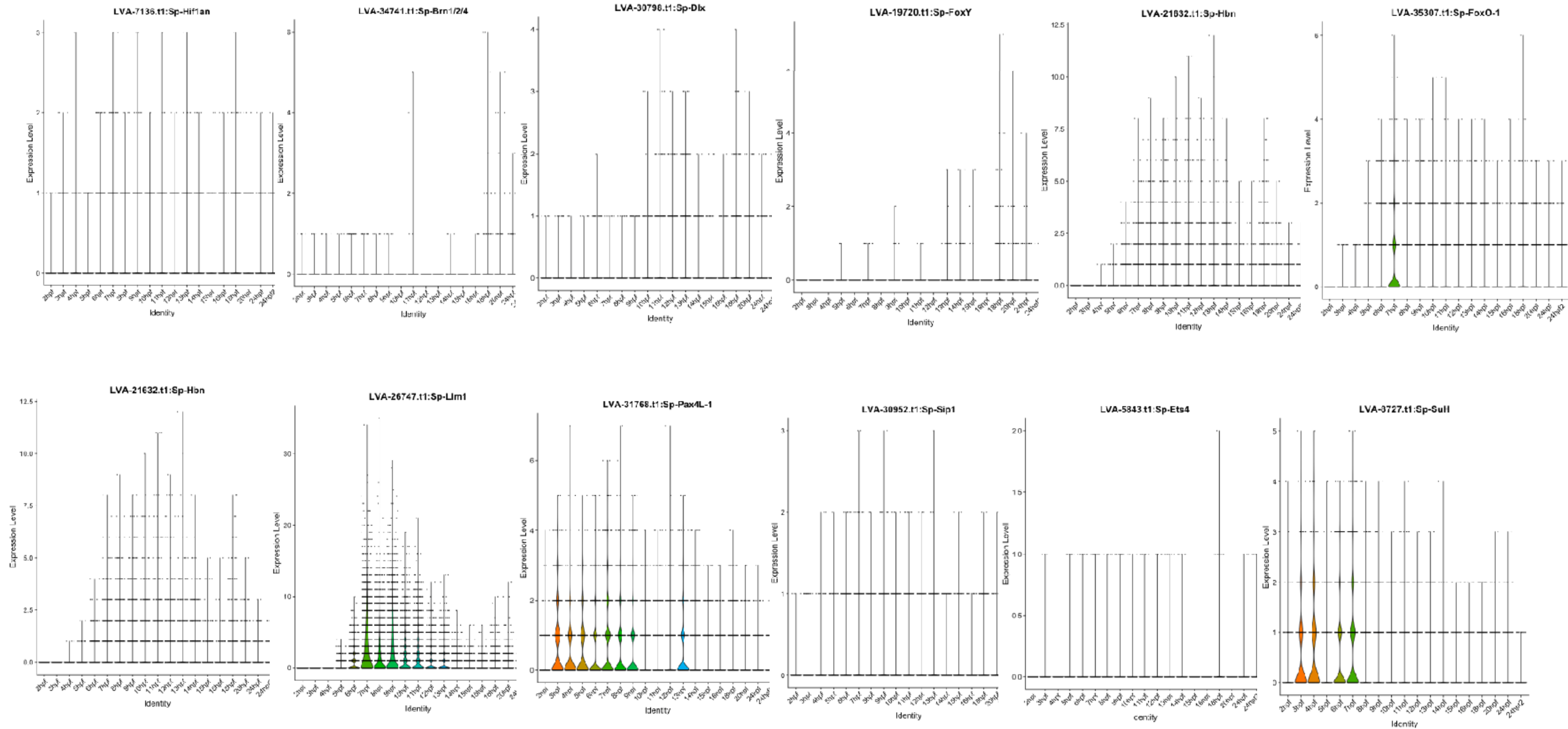

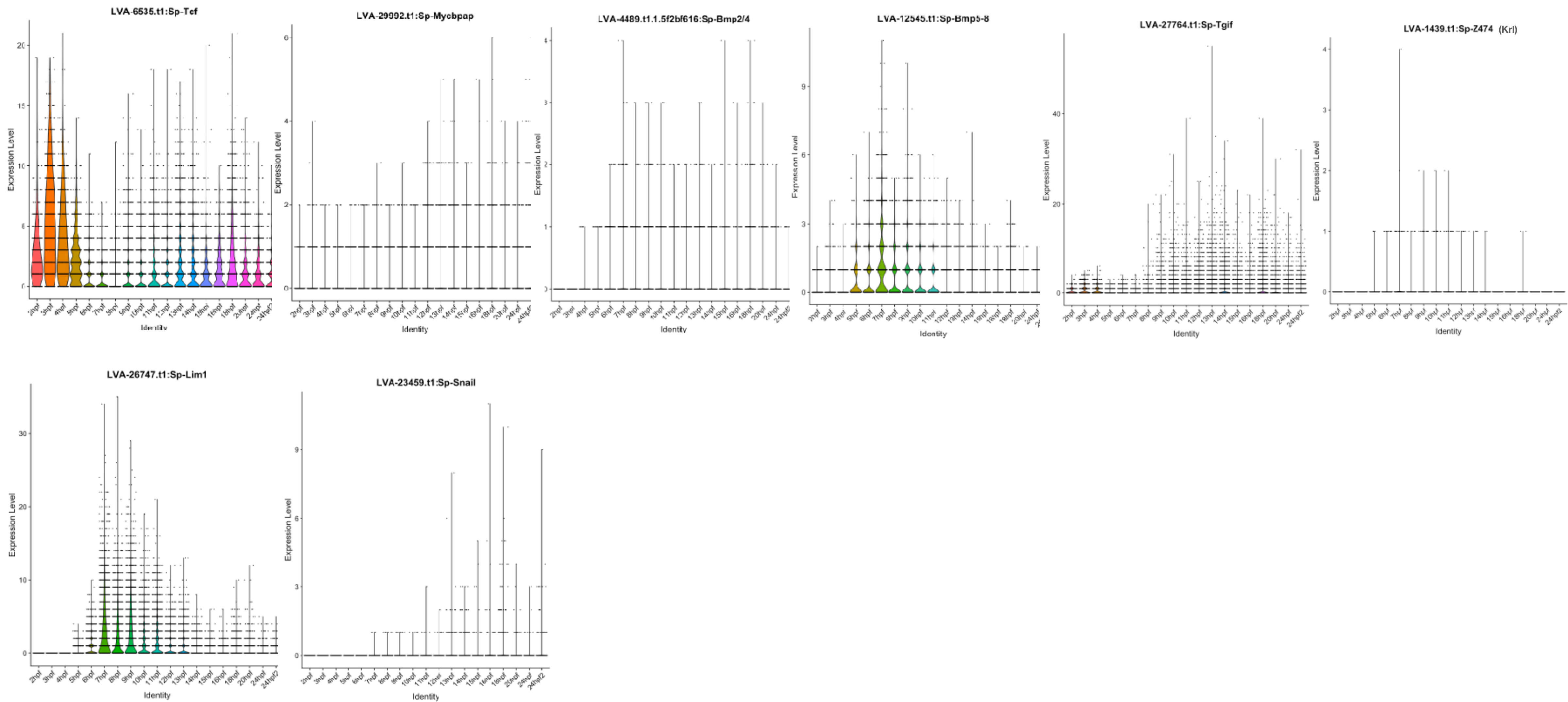

Supplement: Supplementary file 1 — Additional file 1: Figure S1. Data used for selecting time of first expression of Lv dGRN genes. Each of the 81 genes in the dGRN were graphed at each of the 19 time points of that analysis (X axis) Each graph provides the cells expressing that gene at each hour (each cell = dot), and the level of expression by each cell (Y axis). The table was constructed by measuring all cells at each time point since that was the nanostring approach used in the Sp analysis. Further refinement of this method is possible for each lineage but not reflected here. The times selected as earliest times of expression are given in Additional file 5: Table S1. [file 13227_2023_214_MOESM1_ESM.pdf]

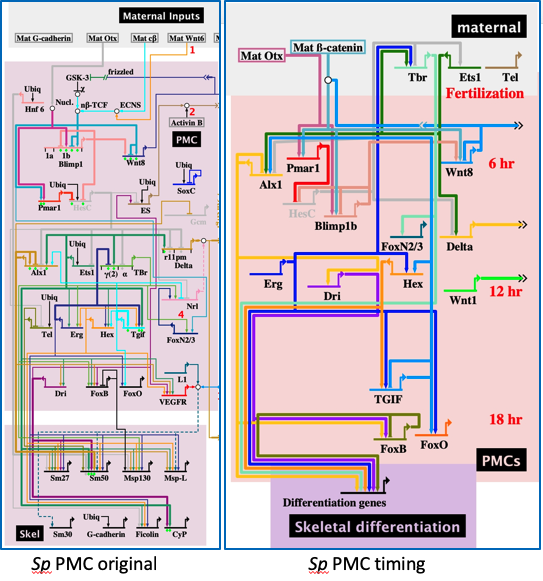

Supplement: Supplementary file 2 — Additional file 2: Fig. S2 A. The original Sp dGRN model of skeletogenic cells and an updated version reflecting time of first expression. The updated version on the right has been simplified by removing genes that have not been independently verified and the several differentiation genes are combined. B. The original Sp dGRN model of endomesoderm and an updated version reflecting time of first expression. The updated version is shown on the right. Mat = maternal, U = unknown activator, Oral NSM (non-skeletal mesoderm) is considered the same as larval ventral NSM and Aboral NSM is considered the same as larval dorsal NSM. C The original Sp dGRN model of ectoderm and an updated version reflecting timing of first expression. The original Sp dGRN model [63](left) reflected a number of ectodermal territories. The updated timing dGRN model (right) is simplified to reflect only the dorsal and ventral regions of ectoderm that are subdivided as a consequence of Nodal signaling [51] [file 13227_2023_214_MOESM2_ESM.zip › Fig. S2A.png]

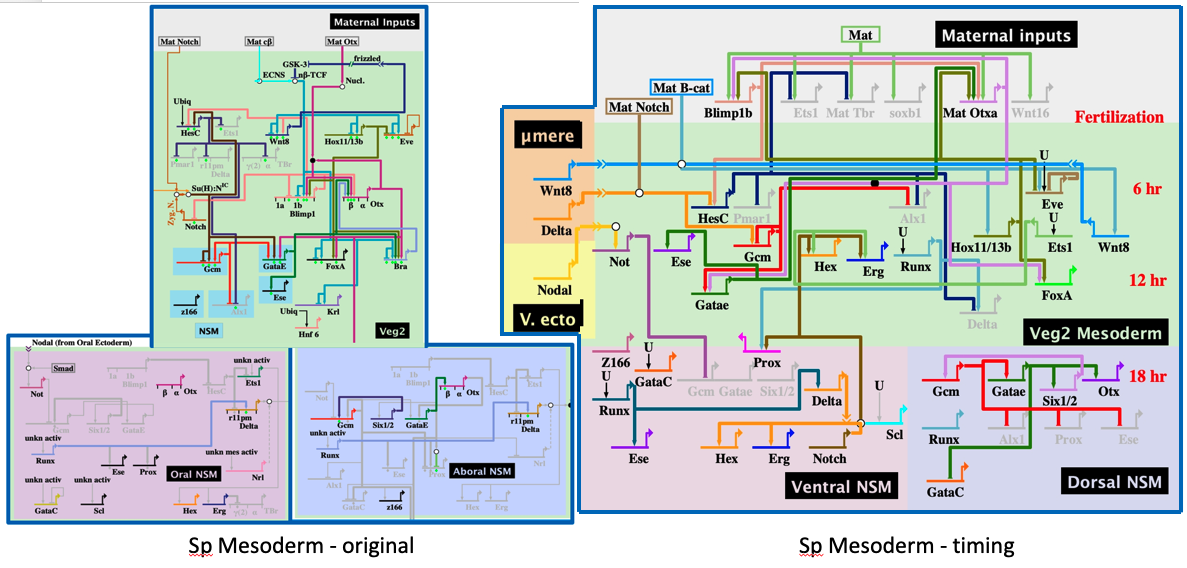

Supplement: Supplementary file 2 — Additional file 2: Fig. S2 A. The original Sp dGRN model of skeletogenic cells and an updated version reflecting time of first expression. The updated version on the right has been simplified by removing genes that have not been independently verified and the several differentiation genes are combined. B. The original Sp dGRN model of endomesoderm and an updated version reflecting time of first expression. The updated version is shown on the right. Mat = maternal, U = unknown activator, Oral NSM (non-skeletal mesoderm) is considered the same as larval ventral NSM and Aboral NSM is considered the same as larval dorsal NSM. C The original Sp dGRN model of ectoderm and an updated version reflecting timing of first expression. The original Sp dGRN model [63](left) reflected a number of ectodermal territories. The updated timing dGRN model (right) is simplified to reflect only the dorsal and ventral regions of ectoderm that are subdivided as a consequence of Nodal signaling [51] [file 13227_2023_214_MOESM2_ESM.zip › Fig. S2b.png]

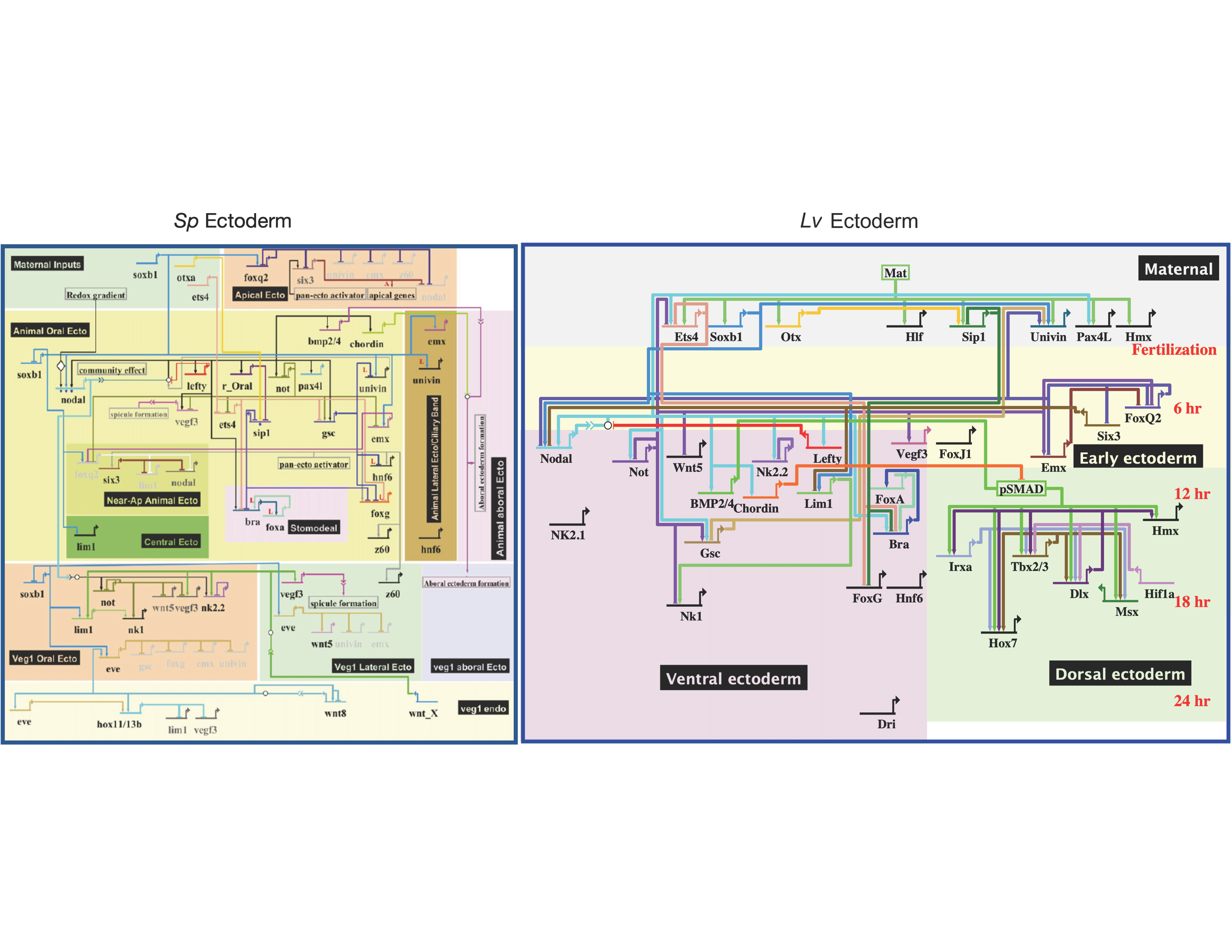

Supplement: Supplementary file 2 — Additional file 2: Fig. S2 A. The original Sp dGRN model of skeletogenic cells and an updated version reflecting time of first expression. The updated version on the right has been simplified by removing genes that have not been independently verified and the several differentiation genes are combined. B. The original Sp dGRN model of endomesoderm and an updated version reflecting time of first expression. The updated version is shown on the right. Mat = maternal, U = unknown activator, Oral NSM (non-skeletal mesoderm) is considered the same as larval ventral NSM and Aboral NSM is considered the same as larval dorsal NSM. C The original Sp dGRN model of ectoderm and an updated version reflecting timing of first expression. The original Sp dGRN model [63](left) reflected a number of ectodermal territories. The updated timing dGRN model (right) is simplified to reflect only the dorsal and ventral regions of ectoderm that are subdivided as a consequence of Nodal signaling [51] [file 13227_2023_214_MOESM2_ESM.zip › Fig. S2C .png]

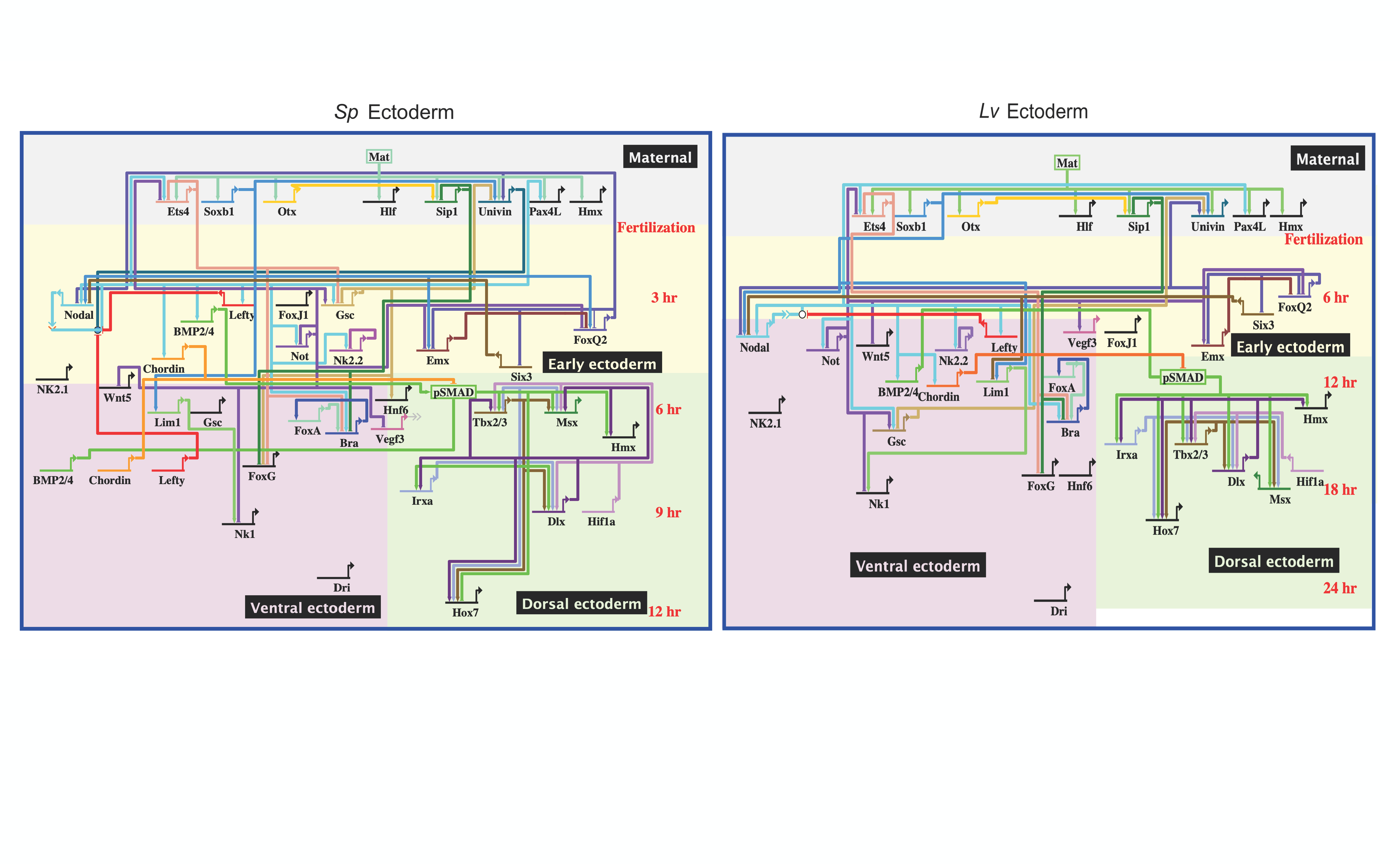

Supplement: Supplementary file 3 — Additional file 3: Fig. S3 A. Ectodermal dGRNs of Sp and Lv redrawn to reflect timing of first expression. Earliest time points are at the top and normalized hours post-fertilization are indicated on the right side of the dGRN models. Maternal genes are in the gray area at the top of each dGRN. The light yellow area shows the generalized early expression of all ectoderm. The purple and light green regions show the subregions that are further specified following Nodal signaling that initiates those regional separations [51]. B. Endodermal dGRNs of Sp and Lv redrawn to reflect timing of first expression. Earliest time points are at the top and normalized hours post-fertilization is indicated on the right side of the GRN models. Maternal genes are in the gray areas. At sixth cleavage an equatorial division separates the veg1 endoderm (light orange) from the veg2 endoderm cells (light yellow). These two regions are then specified somewhat differently. Time of development for both species is in red to the right of each GRN model. C. Skeletogenic mesenchyme dGRNs of Sp and Lv redrawn to reflect timing of first expression. Earliest time points are at the top and normalized hours post-fertilization is indicated on the right side of the GRN models. Maternally expressed genes are in the gray area. The dGRN models are simplified to show inputs into multiple differentiation genes. The Lv skeletogenic mesenchyme model includes snail and twist which were identified and through perturbation studies included in that GRN model [64, 65]. Connections in the Sp GRN model are identical to the Lv model except snail and twist were not tested in Sp [file 13227_2023_214_MOESM3_ESM.zip › Fig. S3A.png]

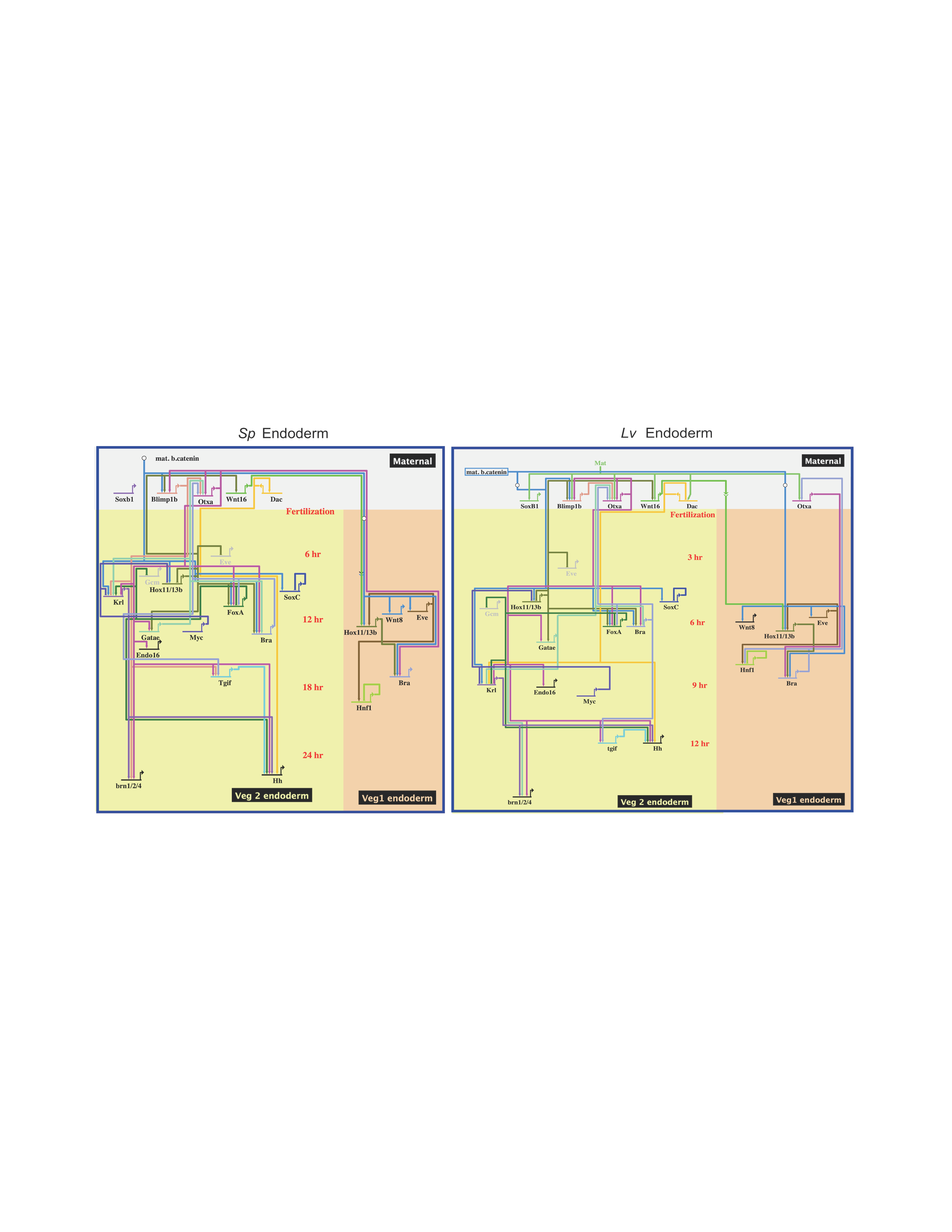

Supplement: Supplementary file 3 — Additional file 3: Fig. S3 A. Ectodermal dGRNs of Sp and Lv redrawn to reflect timing of first expression. Earliest time points are at the top and normalized hours post-fertilization are indicated on the right side of the dGRN models. Maternal genes are in the gray area at the top of each dGRN. The light yellow area shows the generalized early expression of all ectoderm. The purple and light green regions show the subregions that are further specified following Nodal signaling that initiates those regional separations [51]. B. Endodermal dGRNs of Sp and Lv redrawn to reflect timing of first expression. Earliest time points are at the top and normalized hours post-fertilization is indicated on the right side of the GRN models. Maternal genes are in the gray areas. At sixth cleavage an equatorial division separates the veg1 endoderm (light orange) from the veg2 endoderm cells (light yellow). These two regions are then specified somewhat differently. Time of development for both species is in red to the right of each GRN model. C. Skeletogenic mesenchyme dGRNs of Sp and Lv redrawn to reflect timing of first expression. Earliest time points are at the top and normalized hours post-fertilization is indicated on the right side of the GRN models. Maternally expressed genes are in the gray area. The dGRN models are simplified to show inputs into multiple differentiation genes. The Lv skeletogenic mesenchyme model includes snail and twist which were identified and through perturbation studies included in that GRN model [64, 65]. Connections in the Sp GRN model are identical to the Lv model except snail and twist were not tested in Sp [file 13227_2023_214_MOESM3_ESM.zip › Fig. S3B.png]

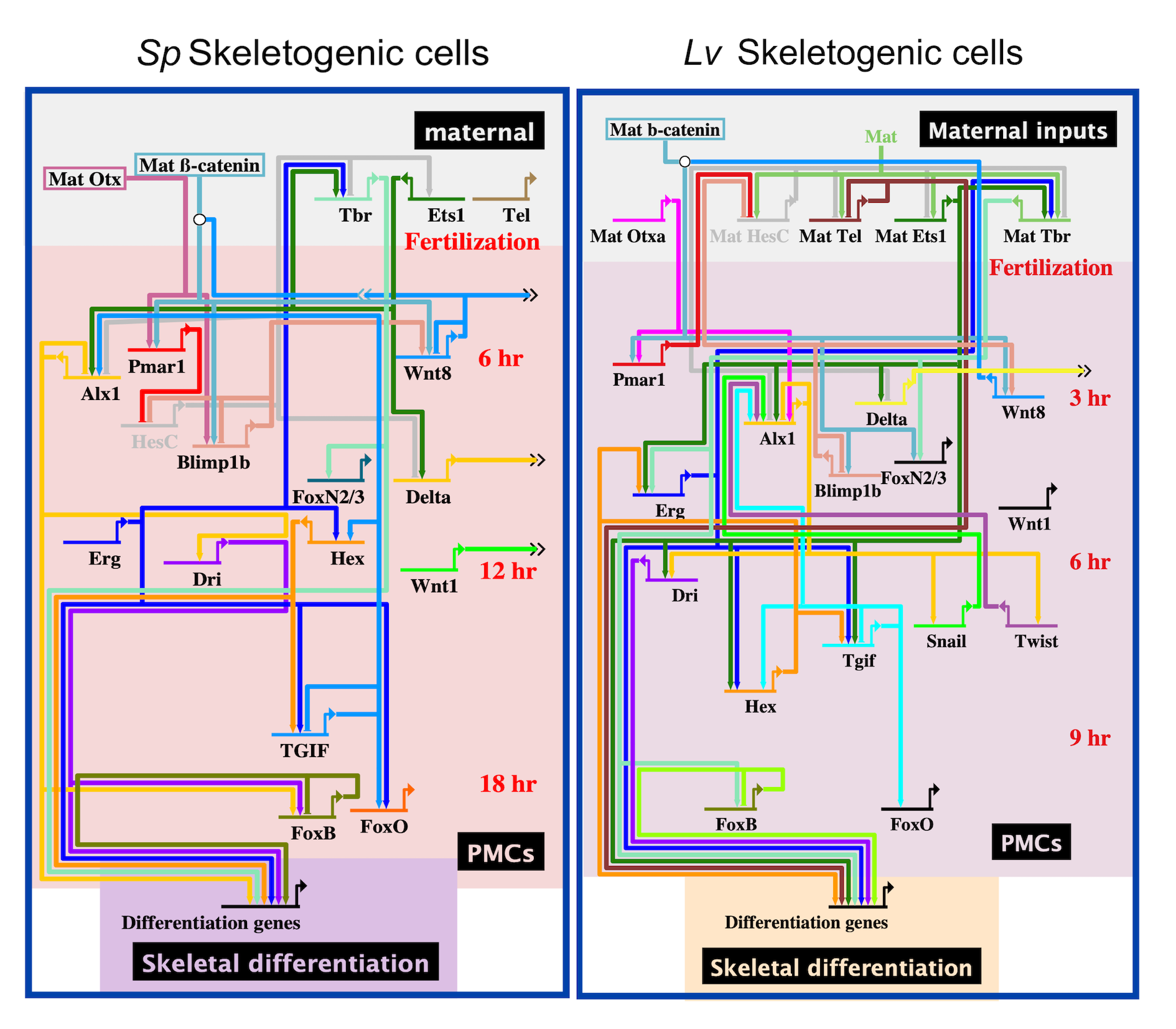

Supplement: Supplementary file 3 — Additional file 3: Fig. S3 A. Ectodermal dGRNs of Sp and Lv redrawn to reflect timing of first expression. Earliest time points are at the top and normalized hours post-fertilization are indicated on the right side of the dGRN models. Maternal genes are in the gray area at the top of each dGRN. The light yellow area shows the generalized early expression of all ectoderm. The purple and light green regions show the subregions that are further specified following Nodal signaling that initiates those regional separations [51]. B. Endodermal dGRNs of Sp and Lv redrawn to reflect timing of first expression. Earliest time points are at the top and normalized hours post-fertilization is indicated on the right side of the GRN models. Maternal genes are in the gray areas. At sixth cleavage an equatorial division separates the veg1 endoderm (light orange) from the veg2 endoderm cells (light yellow). These two regions are then specified somewhat differently. Time of development for both species is in red to the right of each GRN model. C. Skeletogenic mesenchyme dGRNs of Sp and Lv redrawn to reflect timing of first expression. Earliest time points are at the top and normalized hours post-fertilization is indicated on the right side of the GRN models. Maternally expressed genes are in the gray area. The dGRN models are simplified to show inputs into multiple differentiation genes. The Lv skeletogenic mesenchyme model includes snail and twist which were identified and through perturbation studies included in that GRN model [64, 65]. Connections in the Sp GRN model are identical to the Lv model except snail and twist were not tested in Sp [file 13227_2023_214_MOESM3_ESM.zip › Fig. S3C.png]

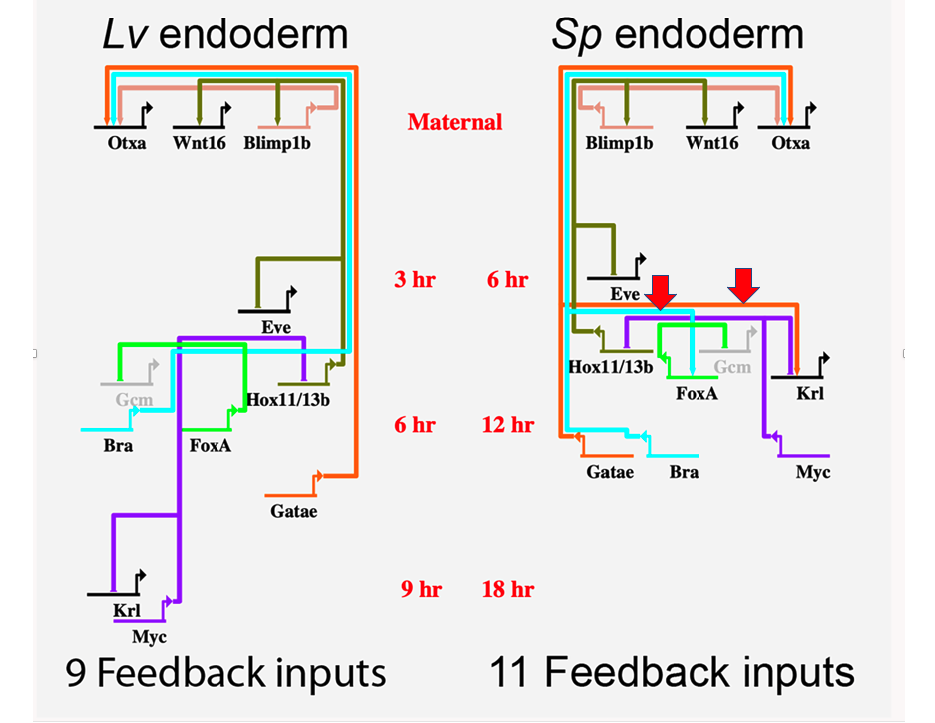

Supplement: Supplementary file 4 — Additional file 4: Fig. S4 A Feedback inputs in the updated Lv and Sp endoderm dGRNs. The diagrams show only the feedback circuits. The red arrows indicate feedbacks that are unique to one of the two species. B. Feedback inputs in the updated Lv and Sp mesoderm. The diagrams show the feedback inputs only in the two dGRNs. The red arrows indicate feedbacks unique to one of the two species. C Feedbacks in the updated Lv and Sp skeletogenic cells. The diagrams show the 10 and 7 feedbacks in the two species. The red arrow indicates a feedback input that is unique to Lv. Two other feedbacks in Lv are snail and twist inputs into alx1. These genes are not incorporated into the Sp dGRN models [file 13227_2023_214_MOESM4_ESM.zip › Fig. S4A.png]

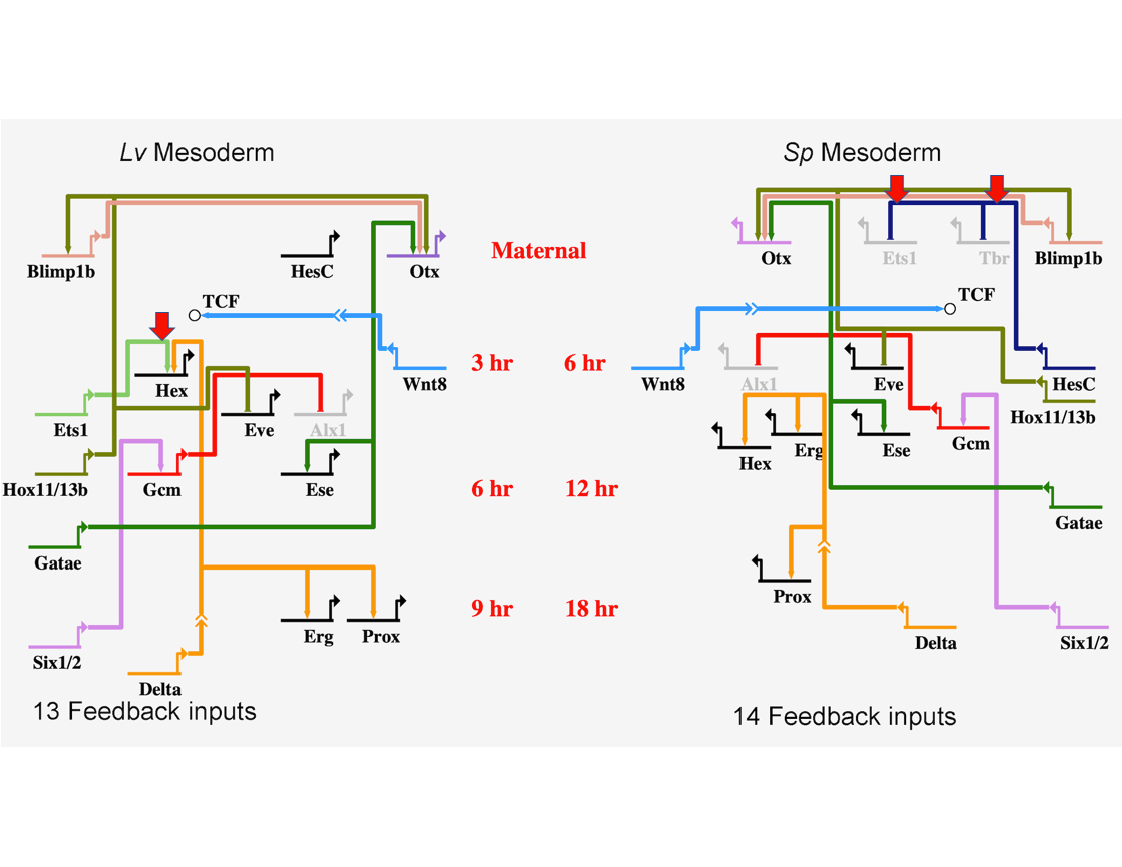

Supplement: Supplementary file 4 — Additional file 4: Fig. S4 A Feedback inputs in the updated Lv and Sp endoderm dGRNs. The diagrams show only the feedback circuits. The red arrows indicate feedbacks that are unique to one of the two species. B. Feedback inputs in the updated Lv and Sp mesoderm. The diagrams show the feedback inputs only in the two dGRNs. The red arrows indicate feedbacks unique to one of the two species. C Feedbacks in the updated Lv and Sp skeletogenic cells. The diagrams show the 10 and 7 feedbacks in the two species. The red arrow indicates a feedback input that is unique to Lv. Two other feedbacks in Lv are snail and twist inputs into alx1. These genes are not incorporated into the Sp dGRN models [file 13227_2023_214_MOESM4_ESM.zip › Fig. S4B.png]

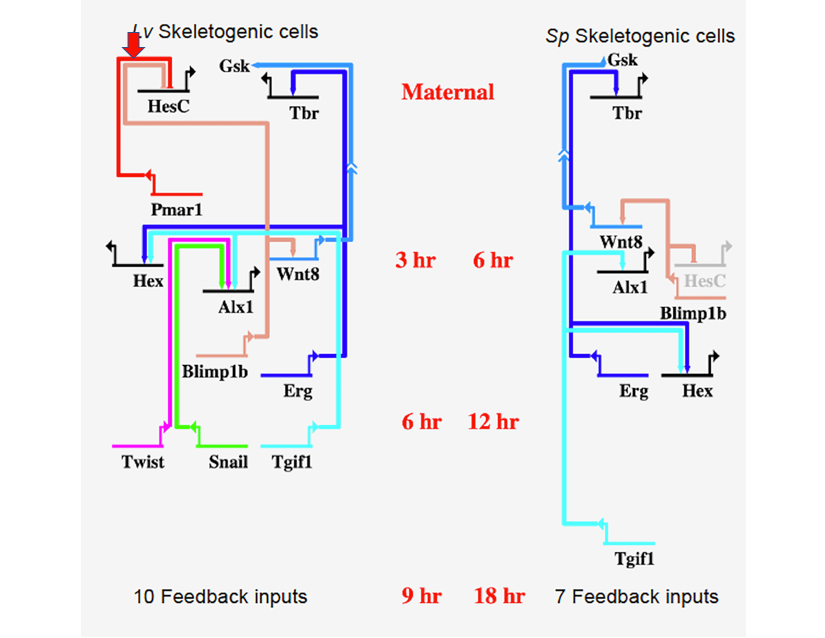

Supplement: Supplementary file 4 — Additional file 4: Fig. S4 A Feedback inputs in the updated Lv and Sp endoderm dGRNs. The diagrams show only the feedback circuits. The red arrows indicate feedbacks that are unique to one of the two species. B. Feedback inputs in the updated Lv and Sp mesoderm. The diagrams show the feedback inputs only in the two dGRNs. The red arrows indicate feedbacks unique to one of the two species. C Feedbacks in the updated Lv and Sp skeletogenic cells. The diagrams show the 10 and 7 feedbacks in the two species. The red arrow indicates a feedback input that is unique to Lv. Two other feedbacks in Lv are snail and twist inputs into alx1. These genes are not incorporated into the Sp dGRN models [file 13227_2023_214_MOESM4_ESM.zip › Fig. S4C.png]
