# Supplementary material for: Feedback circuits are numerous in embryonic gene regulatory networks and offer a stabilizing influence on evolution of those networks
Source: EvoDevo. 2023 Jun 16;14:10. doi: 10.1186/s13227-023-00214-y (PMC10273620; doi:10.1186/s13227-023-00214-y)
Supplement: Supplementary file 5 — Additional file 5: Table S1. Time of first expression of 81 dGRN genes in Lv. Shown are two time points for each gene, the first time point is the last hour of expression of background, and the second time point is the first upward inflection. Fig. S1 illustrates graphs showing cells and level of genes expressed by those cells. All 81 genes were subjected to this analysis for time point selection. [file 13227_2023_214_MOESM5_ESM.pdf]

List	Name	ID	Time hr
ectoderm	Sp-Hif1an	L_var_26265	4 to 5
ectoderm	Sp-Tbx2/3	L_var_04601	5 to 6
ectoderm	Sp-Hmx	L_var_19923	4 to 5
ectoderm	Sp-Hox7	L_var_06457	10 to 11
ectoderm	Sp-Msx	L_var_20199	5 to 6
ectoderm	Sp-IrxA	L_var_03396	7 to 8
ectoderm	Sp-Dlx	L_var_06655	8 to 9
ectoderm	Sp-Bmp2/4	L_var_14096	2 to 3
ectoderm	Sp-Chordin	L_var_21972	4 to 5
ectoderm	Sp-Dr_1	L_var_11305	11 to 12
ectoderm	Sp-E2f3	L_var_02778	4 to 5
ectoderm	Sp-FoxG	L_var_14141	7 to 8
ectoderm	Sp-FoxJ1	L_var_17068	2 to 3
ectoderm	Sp-Gsc	L_var_13092	3 to 4
ectoderm	Sp-HesC	L_var_22399	2 to 3
ectoderm	Sp-Hlf	L_var_00415	2 to 3
ectoderm	Sp-Lhx2	L_var_23544	8 to 9
ectoderm	Sp-Lim1	L_var_05622	5 to 6
ectoderm	Sp-Nk1	L_var_19924	8 to 9
ectoderm	Sp-Nkx2-2	L_var_13912	4 to 5
ectoderm	Sp-Nodal	L_var_26144	4 to 5
ectoderm	Sp-Sip1	L_var_06794	2 to 3
ectoderm	Sp-Klf7	L_var_02281	5 to 6
ectoderm	Sp-Ets4	L_var_16442	2 to 3
ectoderm	Sp-FoxQ2_1	L_var_17031	4 to 5
ectoderm	Sp-Six3	L_var_13793	4 to 5
ectoderm	Sp-Univin	L_var_14095	4 to 5
ectoderm	Sp-Emx	L_var_19379	4 to 5
ectoderm	Sp-Pax4L_1	L_var_07423	2 to 3
ectoderm	Sp-Lefty	L_var_12953	2 to 3
ectoderm	Sp-Vegf3	L_var_00379	5 to 6
ectoderm	Sp-Wnt5	L_var_07687	4 to 5
ectoderm	Sp-Eve	L_var_06470	3 to 4
ectoderm	Sp-Onecut	L_var_11694	4 to 5
ectoderm	Sp-Fgfr	L_var_19622	2 to 3
endomes	Sp-SoxB1	L_var_24060	2 to 3
endomes	Sp-Hox11/13b	L_var_06463	4 to 5
endomes	Sp-Notch	L_var_22864	2 to 3
endomes	Sp-Gatae	L_var_01834	5 to 6
endomes	Sp-FoxA	L_var_14241	6 to 7
endomes	Sp-Bra	L_var_08961	6 to 7
endomes	Sp-Mycbpap	L_var_06005	2 to 3
endomes	Sp-Hh	L_var_06155	10 to 11
endomes	Sp-Hnf1_1	L_var_05010	8 to 9
endomes	Sp-Not	L_var_19596	4 to 5
endomes	Sp-Ese	L_var_15967	4 to 5
endomes	Sp-Prox1	L_var_13096	6 to 7
endomes	Sp-Six1/2	L_var_13791	9 to 10
endomes	Sp-Gcm	L_var_24790	4 to 5
endomes	Sp-Runt1	L_var_08861	4 to 5
endomes	Sp-GataC	L_var_07655	7 to 8
endomes	Sp-Scl	L_var_18807	7 to 8
endomes	Sp-FoxY	L_var_09987	7 to 8
endomes	Sp-Smo	L_var_01467	4 to 5
endomes	Sp-Ptc	L_var_19204	4 to 5
endomes	Sp-Endo16	L_var_26583	9 to 10
endomes	Sp-Z166	L_var_23249	7 to 8
endomes	Sp-SuH	L_var_12277	2 to 3
endomes	Sp-Brn1/2/4	L_var_09396	2 to 3
endomes	Sp-Pks1	L_var_23510	6 to 7
micromere	Sp-Otx	L_var_13089	2 to 3
micromere	Sp-Tcf	L_var_25806	2 to 3
micromere	Sp-Pmar1a	L_var_04869	2 to 3
micromere	Sp-HesC	L_var_22399	2 to 3
micromere	Sp-Blimp1	L_var_08699	4 to 5
micromere	Sp-Ets1/2	L_var_14420	2 to 3
micromere	Sp-Tbr	L_var_02106	2 to 3
micromere	Sp-Wnt8	L_var_26156	3 to 4
micromere	Sp-Alx1	L_var_16422	4 to 5
micromere	Sp-Delta	L_var_13663	3 to 4
micromere	Sp-Tel	L_var_15978	5 to 6
micromere	Sp-Erg	L_var_14555	4 to 5
micromere	Sp-Hexb	L_var_10982	2 to 3
micromere	Sp-Tgif	L_var_25470	7 to 8
micromere	Sp-FoxN2/3	L_var_13332	4 to 5
micromere	Sp-Dr_1	L_var_11305	5 to 6
micromere	Sp-FoxB	L_var_10986	9 to 10
micromere	Sp-FoxO_1	L_var_09670	10 to 11
micromere	Sp-Snail	L_var_03074	6 to 7
