# Supplementary material for: Feedback circuits are numerous in embryonic gene regulatory networks and offer a stabilizing influence on evolution of those networks
Source: EvoDevo. 2023 Jun 16;14:10. doi: 10.1186/s13227-023-00214-y (PMC10273620; doi:10.1186/s13227-023-00214-y)
Supplement: Supplementary file 6 — Additional file 6: Table S2. qPCR of a selection of the dGRN genes. This table shows the 20 genes selected for qPCR and the Cq results at each hour. Highlighted in yellow are the times identified as approximating the inflection point of expression. These times are included in the comparison of Table S3 and all are within an hour of the identified scRNA-seq inflection time points. [file 13227_2023_214_MOESM6_ESM.pdf]

Mean qPCR Cq Values (3 Replicates) for Lv Gene Expression Timecourse												
Gene	0 hr	2 hr	3 hr	4 hr	5 hr	6 hr	7 hr	8 hr	9 hr	10 hr	11 hr	12 hr
Ets	18.57	19.39	19.61	20.27	19.82	19.83	20.43	22.04	21.36	21.93	23.13	21.95
Eve	34.71	32.91	28.28	24.65	22.63	21.65	22.1	22.65	21.1	21.46	21.79	20.93
FoxA	30.04	30.39	30.19	29.94	28.57	27.7	26.54	24.35	21.66	21.84	21.85	20.34
FoxB	33.28	32.38	33.37	33.7	32.65	31.1	31.69	31.93	32.3	32.35	30.67	26.69
GATAc	32.72	32.9	32.76	33.16	33.26	32.66	28.81	27.84	26.32	24.67	24.14	22.62
Gcm	29.76	30.16	30.55	28.31	24.95	23.31	22.36	21.59	20.59	21.06	21.74	21.7
Gsc	31.12	31.68	31.51	31.88	28.75	27.43	28.57	27.06	26.25	25.61	25.29	24.11
Hnf6	21.45	21.98	22.17	22.75	22.33	22.05	21.34	21.75	20.44	21.64	23.82	22.5
Msp130	41.25	45	42.57	39.15	34.28	32.2	25.26	22.77	19.51	19.18	19.48	17.11
Nkx2.1	32.4	33.58	33.43	32.65	28.07	24.52	23.46	24.04	22.84	23.69	25.47	23.67
Nodal	35.48	30.19	28.88	27.26	23.94	23.54	22.19	22.51	22.73	22.86	23.17	22.96
Not	34.21	34.33	34.7	33.27	30.36	28.22	27.42	27.46	27.09	26.46	27.08	25.85
Pks1	25.16	25.87	26	27.04	26.47	26.97	27.77	29.81	23.73	20.61	20.73	17.87
Pmar1	28.88	28.41	25.26	24.12	24.13	25.33	26.33	27.04	26.49	27.51	29.07	28.73
SoxB1	17.82	17.78	18.55	18.96	18.65	18.23	18.31	19.1	18.75	20.18	21.95	20.64
SoxE	30.99	32.04	32.01	31.74	28.85	27.4	26.89	27.71	27.23	28.34	29.4	28.12
Tbr	20.1	20.57	21.04	21.92	21.14	21.64	22.48	22.94	21.44	21.69	22.72	21.25
Wnt8	34.66	34.04	29.4	24.71	22.27	20.81	20.51	21.16	20.45	20.78	21.56	20.67
