# Supplementary material for: Feedback circuits are numerous in embryonic gene regulatory networks and offer a stabilizing influence on evolution of those networks
Source: EvoDevo. 2023 Jun 16;14:10. doi: 10.1186/s13227-023-00214-y (PMC10273620; doi:10.1186/s13227-023-00214-y)
Supplement: Supplementary file 7 — Additional file 7: Table S3. Comparison of Times of first expression of 81 dGRN genes in Sp and Lv. Data from [37] were used to identify time of first expression of Sp genes. Data from [36] were used to approximate time of first expression of Lv genes. A qPCR analysis provided an independent assessment for 19 of the 81 Lv genes. To normalize the times of first expression due to difference in temperature of culture, the time of first expression of Lv is 2X since Lv reaches each stage of development up to gastrulation in half the time needed for Sp to reach that same stage (see Methods). [file 13227_2023_214_MOESM7_ESM.pdf]

Gene	Tissue	Sp time	Lv time(	Relative	qPCR
tel	pmc	mat	Mat	same	
tbr	Pmc	mat	mat	same	mat
ets1/2	pmc	mat	mat	same	mat
otxa	pmc	mat	mat	same	
wnt8	pmc	5	6	same	6
Wnt1	pmc	10	10	same	
Alx1	pmc	6	8	same	
FoxN2/3	pmc	9	10	same	
delta	pmc	9	6	Lv early	
erg	pmc	10	10	same	
dri	pmc	10	12	same	
tgif	pmc	16	14	same	
snail	pmc	19	14	Lv early	
FoxO	pmc	18	20	same	
FoxB	pmc	18	20	same	22
Pmar1	pmc	6	6	same	
otxa	endomes	mat	mat	same	
soxB1	endomes	mat	mat	same	mat
beta catenin	endomes	mat	mat	same	
Eve	endomes	6	8	same	6
blimp1b	endomes	mat-8	mat-10	same	
hox11/13b	endomes	9	10	same	
Notch	endomes	mat	mat	same	
Tcf	endomes	mat	mat	same	
gatae	endomes	13	14	same	
FoxA	endomes	10	12	same	10
Wnt16	endomes	mat	mat	same	
hesC	endmes	7	mat	Lv early	
FoxY	endomes	10	16	Lv late	
not	ecto mes	9	10	same	10
ese	mes	9	10	same	
prox	mes	mat-16	mat-14	same	
six1/2	mes	19	20	same	
runx	mes	10	10	same	
gatac	mes	18	16	same	14
scl	mes	20	16	Lv early	
hex	mes	10	6	Lv early	
erg	mes	9	10	same	
gcm	mes	9	10	same	10
endo16	endo	14	20	Lv late	
brn1/2/4	endo	mat	mat	same	
Bra	endo	13	12	same	
myc	endo	13	20	Lv late	
Hh	endo	22	22	same	
hnf1	endo	17	16	same	
Krl	endo	6	18	Lv late	
hif1a	ectoderm	17	18	same	
tbx2/3	ectoderm	16	12	Lv early	
hmx	ectoderm	12	10	same	
hox7	ectoderm	19	22	Lv late	
msx	ectoderm	18	12	Lv early	
irxa	ectoderm	16	16	same	
dlx	ectoderm	17	18	same	
BMP2/4	ectoderm	12	6	lv early	
Chordin	ectoderm	12	10	same	
Dri	ectoderm	26	22	Lv early	
FoxA	ectoderm	11	12	same	
FoxG	ectoderm	17	16	same	
FoxJ1	ectoderm	9	6	Lv early	
Gsc	ectoderm	15	8	lv early	10
Hlf	ectoderm	mat	mat	same	
Lim1	ectoderm	12	12	same	
Nk1	ectoderm	18	18	same	
NK2.2	ectoderm	10	10	same	
Nodal	ectoderm	9	6	Lv early	6
Sip1	ectoderm	mat	mat	Same	
ets4	ectoderm	mat	mat	same	
FoxQ2	ectoderm	6	8	same	
Six3	ectoderm	8	8	same	
univin	ectoderm	mat	mat	same	
emx	ectoderm	10	10	same	
pax4l	ectoderm	mat	mat	same	
not	ectoderm	10	10	same	
lefty	ectoderm	9	6	Lv early	
vegf3	ectoderm	9	12	Lv late	
soxb1	ectoderm	mat	mat	same	
wnt5	ectoderm	10	10	same	
hnf6 -onecut	ectoderm	mat	mat	same	mat
bra	ectoderm	14	14	same	
nk2.1	ectoderm	14	10	Lv early	
				14 early	
				6 late	
