# Supplementary material for: Feedback circuits are numerous in embryonic gene regulatory networks and offer a stabilizing influence on evolution of those networks
Source: EvoDevo. 2023 Jun 16;14:10. doi: 10.1186/s13227-023-00214-y (PMC10273620; doi:10.1186/s13227-023-00214-y)
Supplement: Supplementary file 8 — Additional file 8: Table S4. Timing of first expression of three species reveals the likely origin of a heterochrony. Data from [38] for 21 genes with timing of first expression normalized to approximate Sp rate of development. Genes in red are expressed at times that differ in Sp relative to the other two species; yellow are genes uniquely different in Pl; genes uniquely different in Lv. Each colored is most likely the species in which the heterochrony originated [file 13227_2023_214_MOESM8_ESM.pdf]

Gene	Sp first on	Pl x.1.3	Lv x 2	Comparison
Wnt8	5	5.2	6	same
FoxQ2	6	5.2	8	same
Hox11/13b	9	8.5	10	same
Blimp1b	9*	8.5	10*	*mat +
Hex	11	7.8	6	Sp late
Gcm	9.8	8.5	10	same
Not1	10	8.5	10	same
Nodal	9	6.5	6	*Sp late*
Alx1	6	8.5	8	same
delta	9	9.75	6	Lv early
FoxA	11	11	12	same
BMP2/4	12	11.7	8	Lv early
Dri	10	12.6	12	same
VegF3	10	13.6	14	Sp early
Bra	13	12.6	12	same
pks1	13	14.3	16	Lv late
gataE	13	14.3	14	same
tbx2/3	17	16.9	12	Lv early
irxa	16	18.2	16	Pl late
dlx	17	18.8	18	same
msx	18	20.1	12	Lv early
six1/2	20	24.7	20	Pl late
