# Supplementary material for: Feedback circuits are numerous in embryonic gene regulatory networks and offer a stabilizing influence on evolution of those networks
Source: EvoDevo. 2023 Jun 16;14:10. doi: 10.1186/s13227-023-00214-y (PMC10273620; doi:10.1186/s13227-023-00214-y)
Supplement: Supplementary file 9 — Additional file 9: Table S5. Summary of feedback circuits in the Sp and Lv dGRNs. The feedbacks recorded in the original Sp dGRNs are summarized as the “classic” models. After consideration of timing of first expression the dGRN models for Sp and Lv show significant increases the presence of positive and negative feedback circuits. [file 13227_2023_214_MOESM9_ESM.pdf]

	Lineage	#	Total	Positive	Negative
Sp classic	Endoderm	7		4	3
	Mesoderm	7		6	1
	PMC	5		4	1
	Ectoderm	5		3	2
		24		71%	29%
Sp timing	Endoderm	10		6	4
	Mesoderm	14		10	4
	PMC	7		6	1
	Ectoderm	13		9	4
		44		70%	30%
Lv timing	Endoderm	9		5	4
	Mesoderm	13		11	2
	PMC	10		8	2
	Ectoderm	18		12	6
		50		72%	28%
